# The Genetic and Environmental Bases of Complex Human-Disease: Extending the Utility of Twin-Studies

**DOI:** 10.1371/journal.pone.0047875

**Published:** 2012-12-18

**Authors:** Douglas S. Goodin

**Affiliations:** Department of Neurology, University of California San Francisco, San Francisco, California, United States of America; Queen's University Belfast, United Kingdom

## Abstract

Making only the assumption that twins are representative of the population from which they are drawn, we here develop a simple mathematical model (using widely available epidemiological information) that sheds considerable light on the pathogenesis of complex human diseases. Specifically, for the case of multiple sclerosis (MS), we demonstrate that the vast majority of patients (≥94%), possibly all, require genetic susceptibility in order to get MS. Nevertheless, only a tiny fraction of the population (≤2.2%) is actually susceptible to getting this disease; a finding which is highly consistent in all of the studied populations across both North America and Europe. Men are more likely to be susceptible than women although susceptible women are more than twice as likely to actually develop MS compared to susceptible men (i.e., they have a greater disease penetrance). This is because women are more responsive to the environmental factors involved in MS pathogenesis than men. These differences account for the current gender-ratio (3∶1, favoring women) and also for the increasing incidence of MS in women around the world. By contrast, the most important genetic marker for MS susceptibility (DRB1*1501) influences the likelihood of susceptibility but not the penetrance of the disease. Nevertheless, even for this major susceptibility allele, only a very small fraction of DRB1*1501carriers (<5%) are susceptible to getting MS and for only a minority of MS patients (∼41%) does this allele contribute to their susceptibility. Moreover, each copy of this allele seems to make an independent contribution to susceptibility. Finally, at least three environmental events are necessary for MS pathogenesis and, during the course of their lives, the large majority of the population (≥69%) experiences an environmental exposure, which is sufficient to produce MS in, at least, some susceptible genotypes. Also, susceptible men (compared to susceptible women) have a lower threshold, a greater hazard-rate, or both in response to the environmental factors involved in MS pathogenesis.

## Introduction

The etiologies of many chronic human-diseases are complex and their basis often includes both the individual's genotype and their environmental experiences [1]. Recurrence-risk data for disease in monozygotic (MZ)-twins, dizygotic (DZ)-twins, and siblings (S) of an affected-proband, provides insight to the nature of disease-susceptibility [2]. For example, if the disease-risk in MZ-twins of the affected-proband is substantially greater than the risk in DZ-twins, this suggests the importance of genetics to disease pathogenesis. Similarly, if this disease-risk is considerably less than 100% in MZ-twins, this suggests of the importance of environmental-factors. In fact, by assuming that both MZ- and DZ-twins have similarly “shared environments” and that twins are representative (genetically) of the general population, the difference in disease-risk between MZ-twins and DZ-twins can estimate the proportion of the variance in disease-occurrence that can be attributed to heritable-factors, shared environmental-factors, or non-shared environmental-factors [2].

While these approaches offer a broad outline of disease pathogenesis, epidemiological data could potentially provide more quantitative information. Here we develop a simple mathematical model, using concordance (recurrence-risk) data from twin and familial studies, to elucidate the nature and frequency of genetic-susceptibility to complex human-diseases. This is not to downplay the importance of environmental factors in disease pathogenesis, which, as noted both here and elsewhere [1]–[4], is considerable. Neither is this an exploration of the genes themselves. Rather, it is an attempt to understand the nature of genetic-susceptibility, the importance of environmental-risk, and to delineate the constraints on the genetic and environmental bases of these complex diseases, which are imposed by certain epidemiological observations or facts.

Although broadly applicable to many chronic complex diseases, these principals are here applied specifically to the example of multiple sclerosis (MS), because of the ready-availability of both familial-recurrence data and world-wide epidemiological information [3]–[21]. For example, it is well-established that the prevalence of MS in the northern regions of either Europe or North America is approximately 0.1–0.2% [3]. For individuals with an affected family member, the MS-risk increases roughly in proportion to the amount of shared genetic-information between the affected-relative and the individual [3], [6], [9], [13], [17], [20]. Although at least three environmental-factors, each acting at specific periods during a person's life, seem critical to disease pathogenesis [4], genetic-factors are also, unquestionably, part of a causal pathway leading to MS [3]–[21].

The earliest and the best-established genetic-association with MS-susceptibility is the HLA-DRB1 locus on the short-arm of chromosome six [21]–[26]. Within this locus, the DRB1*1501 allele has the strongest and most consistent association with MS in both northern European and North American populations [21]–[26]. Nevertheless, despite its importance, only about half of MS patients are DRB1*1501 carriers and only a small percentage of carriers (<1%) will ever develop the disease [21]–[26]. These observations indicate that other genes, at different locations, are necessary and/or sufficient to produce MS-susceptibility [26], [27].

## The Genetic Model

The definitions for the principle model terms are presented in Table 1. Further model definitions, assumptions, and explanatory tables are presented in Appendix S1; Section A. In addition, Appendix S1 presents both the conceptualizations of genetic-susceptibility and environmental-risk used for the model (Section B) as well as a rigorous presentation of model development (Sections C–F). The basic epidemiological and familial-concordance data used for quantitative analysis of model implications are presented in Tables 2,3,4, and the detailed MZ-twin data regarding DRB1*1501 and gender are presented in Tables 5&6. The principle conclusions and range-estimates derived for the model are summarized in Table 7.

**Table 1:** Model definitions*

*P(MS)*	=	The life-time probability of developing MS in the general population.[equated to the prevalence of the disease]
*(G) , (G–)*	=	Sets of persons who either are *(G)* or are not *(G–)* genetically susceptible to MS
*(G1) , (G2)*	=	Two mutually exclusive subsets of *(G)*; one consisting of high-penetrance genotypes *(G1)* and the other consisting of low-penetrance genotypes *(G2). (G1) + (G2) = (G)*
*(G0) , (G3)*	=	Mutually exclusive sets of genetically susceptible individuals who depend upon *(G0)* or don’t depend upon *(G3)* environmental events to get MS. *(G0) + (G3) = (G)*
*P(MS|G–)*	=	Penetrance of the least penetrant genotype in the population
*P(MS|G_i_)*	=	Penetrance of the i^th^ genotype in the set *(G)*
*P(MS|G)*	=	Expected penetrance of the set *(G) ; P(MS|G) = E{P(MS|G_i_)}*
σ_zi_^2^	=	Penetrance Variance within the set *(G) ; σ_zi_^2^ = Var(G_i_)*
*P(MS|IG_MS_)*	=	**b** = the conditional life-time probability of developing a MS, given that the person’s MZ-twin has MS; adjusted to exclude the impact of twins sharing intra-uterine *(IU)* and childhood *(CH)* environments.
*(MZ_MS_) , (DZ_MS_) , (S_MS_)*	=	Sets of persons with a monozygotic *(MZ)*-twin, a dizygotic *(DZ)*-twin, or a sibling (S) who either has or will develop MS.
*(IU) , (CH)*	=	Sets of persons who share, with an MS-proband, either the same intra-uterine *(IU)* or a similar childhood *(CH)* environment
*(E) , (E–)*	=	Sets of persons who either do *(E)* or do not *(E–)* experience a sufficient environmental exposure to produce MS (see Section B)
*(FT) , (ST)*	=	The sets of first *(FT)* or second *(ST)* twins of an MZ-twin pair
*(Gx+) , (Gx–)*	=	The set of persons who either possess *(Gx+)* or don’t possess *(Gx–)* the particular genetic characteristic *(Gx)*.
*(HLA+) , (HLA–)*	=	The set of persons who either carry *(HLA+)* or don’t carry *(HLA–) *at least one HLA DRB1*1501 allele. *(HLA+) = (2HB+) + (1HB+)*
*(1HB+) , (2HB+)*	=	The sets of persons who carry one *(1HB+)* or two *(2HB+)* copies of the DRB1*1501 allele.
*(1HB–)*	=	The set of persons who carry one copy of a non-DRB1*1501 allele 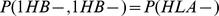 ; 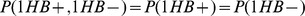
*(F) , (M)*	=	Sets consisting of either women *(F)* or men *(M)*
**a** , **a’**	=	*P(MS, G) / P(G1)* = **a** ; and: *P(MS, G) / P(G2)* = **a’**
**b** , **b’**	=	*P(MS|IG_MS_)* = **b** ; and: *P(MS|G, IG_MS_) * = **b’**
**x**	=	*P(MS|G1)* = Expected Penetrance of the high-penetrance subset
**y**	=	*P(MS|G2)* = Expected Penetrance of the low-penetrance subset
**z**	=	*P(MS|G) * = Expected Penetrance for the entire set *(G)*
**z_t_** , **z_s_**	=	*P(MS|G, Gx+)* = **z_t_** ; and: *P(MS|G, Gx–)* = **z_s_**
**t**	=	*P(MS|Gx+, IG_MS_) = P(MS, G|Gx+, IG_MS_)*
**t’**	=	*P(MS|G, Gx+, IG_MS_)*
**s**	=	*P(MS|Gx–, IG_MS_) = P(MS, G|Gx–, IG_MS_)*
**s’**	=	*P(MS|G, Gx–, IG_MS_)*
p	=	*P(G1|G) = P(G1, G) / P(G) = P(G1) / P(G) ; (G1) ⊂ (G)*
g	=	*P(G|MS) = P(G|IG_MS_)*
g_1_	=	*P(G|Gx+, MS) = P(G|Gx+, IG_MS_)*
g_2_	=	*P(G|Gx–, MS) = P(G|Gx–, IG_MS_)*
A_0_	=	*P(Gx+)*
A	=	*P(Gx+|MS) = P(Gx+|IG_MS_)*
MAF	=	Mean allelic frequency – defined as the frequency of an “allelic state”{e.g., the “*(HLA–)* allele” at the DRB1 gene = one “non-1501” allele}
HWE	=	Hardy-Weinberg Equilibrium

* See Appendix S1 (Section A) for additional model definitions.

**Table 2:** Epidemiological data used in the model^#^

	Population	Women	Men
*P(MS)* = *P(IG_MS_)* *	0.0015	0.00204	0.00096
*P(F|MS)* = *P(F|IG_MS_)* *	0.68		
*P(F|MS, MZ_MS_)* = *P(F|MS, IG_MS_)* *	0.92		
*P(F|HLA+, MZ_MS_)* = *P(F|HLA+, IG_MS_)* *	0.74		
*P(F|MS, HLA+, MZ_MS_)* = *P(F|MS, HLA+, IG_MS_)* *	> 0.82†		
Raw MZ-twin Concordance = *P(MS|MZ_MS_)* *	0.25	0.34	0.067
Adjusted MZ-twin Concordance = *P(MS|IG_MS_)* = b **	0.134	0.183	0.036
Raw DZ-twin Concordance = *P(MS|DZ_MS_)* *	0.054	0.051	0.057
Raw Sibling Concordance = *P(MS|S_MS_)* *	0.029	0.039	0.019
UCSF (#1) – *P(HLA+|MS)* = *P(HLA+|IG_MS_)* – Cases ††	0.56	0.57	0.52
Canadian – *P(HLA+|MS)* = *P(HLA+|IG_MS_)* – Cases ##	0.55	0.60	0.52
Canadian – *P(HLA+)* – Controls ##	0.24	∼ 0.24	∼ 0.24
UCSF (#2) – *P(HLA+|MS)* = *P(HLA+|IG_MS_)* – Cases ††	0.46	0.49	0.39
UCSF (#2) – *P(HLA+)* – Controls ††	0.20	0.18	0.22

# HLA+  =  carrier of ≥ 1 copy of the DRB1*1501 allele

* From Canadian Data [21], based on a prevalence of 150 per 10^5^ population and split into men and women according to [15]. Concordance rates presented as “proband-wise” rates [30].

† Data unavailable on the 2 male patients [21]. The worst case is: 9/11  =  0.82

** See: Prop. (1.4) of Appendix S1 (Section C)

## Canadian HLA data: D Sadovnick (personal communication). Based on ∼ 3,000 cases and ∼ 400 Controls (% women not available). Control rates confirmed in a much larger transplant database.

†† UCSF Databases: J Oksenberg (personal communication) UCSF #1 (GeneMSA) - 485 cases (68% women) and 431 Controls (66% women) UCSF #2 (IMSGC) - 779 cases (76% women)

**Table 3:** HLA data used in the model^#^.

				2HB+		1HB+	HLA−
			**Canadian Data**				
			Observed Frequency – Cases (HLA+ and HLA−)##		0.55		0.45
			Observed Frequency – Controls (HLA+ and HLA−)##		0.24		0.76
			OR – (2HB+ & 1HB+) vs. (HLA−)*		3.9		
			**UCSF #1**				
			Observed Frequency – Cases†	0.10		0.46	0.44
			Predicted HWE frequencies – Cases††	0.11		0.45	0.44
			Predicted Controls – HWE at: P(HLA+) = 0.24	0.016		0.224	0.76
			OR – (2HB+) vs. (HLA−) & (1HB+) vs. (HLA−) *	10.4		3.6	
			OR – (2HB+ & 1HB+) vs. (HLA−)*		4.0		
			**UCSF #2**				
			Observed Frequency – Cases†	0.07		0.39	0.54
			Predicted HWE frequencies – Cases††	0.07		0.39	0.54
			Observed Frequency – Controls†	0.012		0.186	0.80
			Predicted HWE frequencies – Controls††	0.011		0.186	0.80
			OR – (2HB+) vs. (HLA−) & (1HB+) vs. (HLA−) *	9.3		3.1	
			OR – (2HB+ & 1HB+) vs. (HLA−)*		3.5		

# Numbers listed are genotype frequencies. 2HB+ = carrier of 2 copies of the DRB1*1501 allele (homozygous carrier). 1HB+ = carrier of 1 copies of the DRB1*1501 allele (heterozygous carrier). HLA− = carrier of 0 DRB1*1501 alleles. (HLA+) = (2HB+)+(1HB+).

## Canadian HLA data: D Sadovnick (personal communication). Based on ∼3,000 cases and ∼400 Controls (% women not available). Control rates confirmed in a much larger transplant database.

* Odds ratio (OR) versus controls. Calculated as odds of genotype in cases divided by odds of the same genotype in controls.

† UCSF Databases: J Oksenberg (personal communication). UCSF #1 (IMSGC) – 779 cases (76% women); No observed controls. UCSF #2 (GeneMSA) – 485 cases (68% women) and 431 Controls (66% women).

†† Hardy Weinberg Equilibrium (HWE) values predicted based on the observed P(2HB+) in Cases or Controls.

**Table 4:** HLA data by gender used in the model^#^.

	2HB+		1HB+	HLA−
**UCSF #1 (Women)**				
Observed Frequency – Cases†	0.11		0.46	0.43
Predicted HWE frequencies – Cases††	0.11		0.44	0.45
OR – (2HB+ & 1HB+) vs. (HLA−)*		4.3		
**UCSF #1 (Men)**				
Observed Frequency – Cases†	0.06		0.45	0.48
Predicted HWE frequencies – Cases††	0.09		0.42	0.48
OR – (2HB+ & 1HB+) vs. (HLA−)*		3.4		
**UCSF #2 (Women)**				
Observed Frequency – Cases†	0.08		0.41	0.51
Predicted HWE frequencies – Cases††	0.08		0.41	0.50
Observed Frequency – Controls†	0.01		0.17	0.82
Predicted HWE frequencies – Controls††	0.01		0.21	0.78
OR – (2HB+) vs. (HLA−) & (1HB+) vs. (HLA−) *	9.7		3.9	
OR – (2HB+ & 1HB+) vs. (HLA−)*		4.4		
**UCSF #2 (Men)**				
Observed Frequency – Cases†	0.05		0.35	0.61
Predicted HWE frequencies – Cases††	0.05		0.34	0.61
Observed Frequency – Controls†	0.01		0.22	0.78
Predicted HWE frequencies – Controls††	0.01		0.21	0.78
OR – (2HB+) vs. (HLA−) & (1HB+) vs. (HLA−) *	8.6		2.0	
OR – (2HB+ & 1HB+) vs. (HLA−)*		2.2		

# Numbers listed are genotype frequencies. 2HB+ = carrier of 2 copies of the DRB1*1501 allele (homozygous carrier). 1HB+ = carrier of 1 copies of the DRB1*1501 allele (heterozygous carrier). HLA− = carrier of 0 DRB1*1501 alleles; (HLA+) = (2HB+)+(1HB+).

† UCSF Databases: J Oksenberg (personal communication). UCSF #1 (IMSGC) – 779 cases (76% women). UCSF #2 (GeneMSA) – 485 cases (68% women) and 431 Controls (66% women).

†† Hardy Weinberg Equilibrium (HWE) values predicted based on the observed P(2HB+) in Cases or Controls. Because of the small number of men in these samples, the number of males with 2 copies of HLA DRB1*1501 was tiny. Therefore, in men, HWE was estimated from the observed P(HLA−).

* Odds ratio (OR) versus controls. Calculated as odds of genotype in cases divided by odds of the same genotype in controls.

**Table 5:** MS concordance rates in monozygotic twins of DRB1*1501 carrier *(HLA+)* and DRB1*1501 non-carrier *(HLA–)* probands*.

	Monozygotic Twins of MS Probands		
	***HLA+***	***HLA–***	**Totals**
**Concordant for MS (C)**	9	11	20
**Discordant for MS (D)**	31	42	73
**Totals**	40	53	93
**Pair-wise Concordance** †	(9/40) = 0.225	(11/53) = 0.207	0.215
**Proband-wise Concordance** ††	0.309	0.287	0.297
**Proband-wise Concordance (Adjusted)** †††	**t** = 0.166	**s** = 0.154	**b = ** 0.160
**Proband-wise Concordance (Adjusted)** ††††	**t** = 0.139	**s** = 0.129	**b = ** 0.134
***P(HLA+***|***MS, IG***_MS_)** (Adjusted)** #	0.57		

* *(HLA+)*  =  carrier of ≥1 copy of the DRB1*1501 allele; Data from: Reference [21].

† Pair-wise rates (*Z_1_*) calculated as: 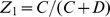; see Reference [30]

†† Proband-wise concordance rates (*Z_2_*) calculated as: 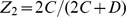; adjusted [30] for the overall probability of doubly ascertaining concordant twin-pairs 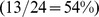
in the study of Willer, et al. [21]

††† For adjustment: See: Prop. (1.4a) 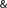 (1.4b) of Appendix S1 (Section C)

†††† Further adjusted to the requirement that: **b**  =  0.134

# Adjusted to the condition where: 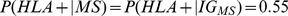

**Table 6:** MS concordance rates in monozygotic twins of female *(F)* and male *(M)* probands*.

	Monozygotic Twins of MS Probands		
	*F*	*M*	Totals
**Concordant for MS (C)**	22	2	24
**Discordant for MS (D)**	66	43	109
**Totals**	88	45	133
**Pair-wise Concordance** †	(22/88) = 0.25	(2/45) = 0.044	0.18
**Proband-wise Concordance** ††	0.34	0.067	0.25
**Proband-wise Concordance (Adjusted)** †††	**t** = 0.183	**s** = 0.036	**b = ** 0.134
***P(F***|***MS, IG***_MS_)** (Adjusted)** #	0.92		

* *(F)*  =  Women ; *(M)*  =  Men ; Data from: Reference [21]

† Pair-wise rates (*Z_1_*) calculated as: 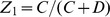; see Ref. [30]

†† Proband-wise concordance rates (*Z_2_*) calculated as: 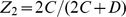; adjusted [30] for the overall probability of doubly ascertaining concordant twin-pairs 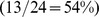 in the study of Willer, et al. [21]

††† For adjustment: See: Prop. (1.4a) & (1.4b) of Appendix S1 (Section C)

# Adjusted to the condition where: 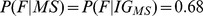

**Table 7:** Summary of conclusions regarding MS pathogenesis derived from the model*

**Conclusions about genetic susceptibility (in general)** (see Section C; Props. 4 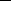 5)	
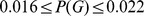	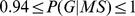
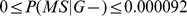	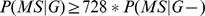
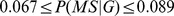	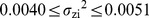
**Conclusions about DRB1** * **1501 status and genetic susceptibility** (see Sections D 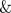 E; Props. 6.3 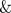 7.1a)	
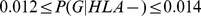	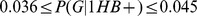
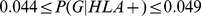	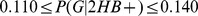
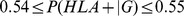	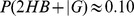
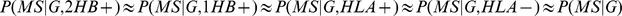	
**Conclusions about gender status and genetic susceptibility** (see Sections D 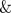 E; Props. 6.2 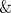 7.1a)	
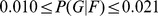	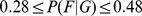
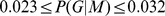	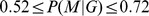
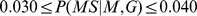	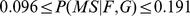
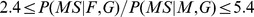	
**Conclusions about other relationships regarding genetic susceptibility** (see Section E; Prop. 7.2)	
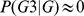 ; where, by definition: 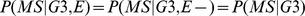	
**Conclusions about environmental susceptibility** (see Section F; Eqs. 24 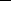 31)	
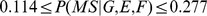	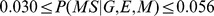
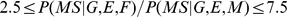	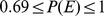
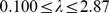 ; λ = threshold difference between women and men – (see Section F)	
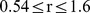 ; r = proportional hazard for MS – women to men – (see Section F)	

* See Table 1 for term definitions; In Table “Section” refers to Sections of Appendix S1

We define disease-penetrance as the conditional life-time probability of disease given the specific genotype for a member of the general population (see Appendix S1; Section B). We can also partition the general population into the mutually exclusive sets of carriers *(HLA+)* and non-carriers *(HLA−)* of at least one copy of the DRB1*1501 allele.

In MS, it is well established [21]–[26] that: 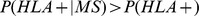
Therefore, it must also be the case that: 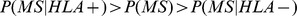
This last statement indicates, unequivocally, that some genotypes have a greater penetrance than others and, therefore, that at least one genotype must have the least penetrance of any. Consequently, the individual genotypes can be partitioned into two subsets, *(G)* and *(G−)*, where the term *P(MS|G−)* or, more generally, *P(D|G−)*, represents the disease-penetrance of the least-penetrant genotype in the population (see Appendix S1; Section B).

It could be the case that: 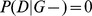


However, if so, and if we define (Table 1) disjoint sets of individual environmental experiences or exposures that either are *(E)* or are not *(E−)* sufficient to produce disease environmentally (see Appendix S1; Sections A&B), then this circumstance requires that:
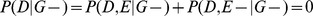
and, thus, that: 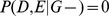


Consequently, the circumstance in which: 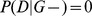; implies that “purely environmental” disease does not occur (see Appendix S1; Sections A&B).

Conversely, if “purely environmental” disease is possible, then:
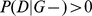



Members of the subset *(G)* are said to be “genetically susceptible” whereas members of the subset *(G−)* are said to be “genetically non-susceptible”. In this conceptualization, genetic-susceptibility is, by definition, binary (quantitatively) although the subset of susceptible individuals *(G)* could, at least theoretically, encompass virtually the entire population (i.e., all but one genotype) and the penetrance of the different susceptible genotypes within *(G)* could range from nearly zero to one (Appendix S1; Section B). The terms *P(D|G)* and (**z**) are used interchangeably and represent the expected disease-penetrance in genetically susceptible individuals. In the model, we imagine that, within the population of all susceptible-individuals *(G)*, each individual has their own individual-specific susceptibility-genotype, and each genotype has its own genotype-specific penetrance-value. The penetrance of disease for the (i^th^) genotype *(G_i_)* within *(G)* is represented as either *P(D|G_i_)* or (**z**_i_). The term *P(D)* represent the probability that a random member of the general population will develop the disease within their life-time. The set *(D,G−)* represents those cases of disease, which occur in individuals who are not genetically susceptible. We also consider the different circumstances that exist for men *(M)* and women *(F)* and, in addition, we partition the *(HLA+)* subset into those individuals who carry either one *(1HB+)* or two *(2HB+)* copies of the DRB1*1501 allele.

Without making any assumptions, two definitional statements can be made:
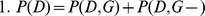


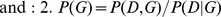


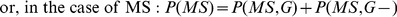
(1)

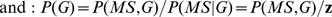
(2)From Equation (1), there must be some constant (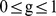) such that:
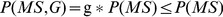
(3)and that: 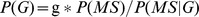; and also: 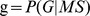


Moreover, because some MS-cases involve genetic-factors [3], [6], 9,13,17,20, then it also must be the case that: 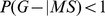; or, equivalently: 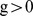


The purpose of the model is to use directly observable epidemiological information of the type presented in Tables 2,3,4,5,6 in order to estimate a variety of unknown quantities including:

*P(G), P(G|MS)*, *P(F|G)*, *P(G|HLA+)*, *P(G|HLA−)*, *P(G|2HB+)*, *P(G|1HB+)*, *P(MS|G)*, *P(MS|G−)*, *P(MS|G,HLA+)*, *P(MS|G,HLA−)*, *P(MS|G,F)*, *P(MS|G,M)*, *P(MS|G,2HB+)*, *P(E)*, *P(MS|E,G,F)*, and *P(MS|E,G,M)*.

In addition, we also use this information to provide other insight to the nature and basis of genetic-susceptibility in different sub-populations.

### Basic model assumptions and derivations

To begin, we define *P(M
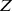
_MS_)* as the life-time probability that, for an individual from an MZ-twinship, their co-twin either has or will develop MS, independent from whatever has happened or will happen to them. Because there is no known genetic-predilection for having MZ-twins, the genetic composition of the MZ-twin population is assumed to be “representative” of the general population. The definition of “representative” is made explicit by Assumptions (A5)&(A6) – Appendix S1 (Section A). Thus, it is assumed (Appendix S1; Section A; Assumption A5) that *P(M
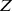
_MS_)* for the first twin *(FT)* is the same as it is for the second twin *(ST)*, and that the genetic-composition of the sets *(MS)* and *(M
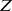
_MS_)* are the same. In this case:
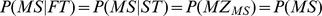



Moreover, it is assumed (Appendix S1; Section A; Assumption A6) that the genetic-composition of the sets *(G,FT)*, *(G,ST)*, and *(G)* are the same. Under these conditions:
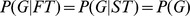



Importantly, for MS, the direct observational data supports the validity of the assumption that twins are “representative”. Thus, both the twin-rates in an MS-population and the probability of MS in twins are as expected for the population as a whole [21]. These same assumptions also underlie the “classical” twin methods discussed earlier [2].

In addition, we assume that *P(MS)* is approximately equal to the observed prevalence of MS in the general population (Appendix S1; Section A; Assumption A1). Nevertheless, because most prevalence-estimates use, as their denominator, the total population in the region and, because almost all MS cases begin (clinically) between the ages of 15 and 45 years [3] and most survive at least into late middle-age [28], Assumption (A1), almost certainly, underestimates *P(MS)*. A better estimator of *P(MS)* – the life-time risk of MS – will be derived from the prevalence in those aged 45–55 years (Appendix S1; Section A). In this age-bracket, new incident-cases are unlikely to occur [3] and substantial early mortality from MS is unlikely to have yet happened [28]. If so, the true *P(MS)* could, potentially, be double the estimate derived from the population-prevalence (e.g., [29]). The impact of this possibility is considered further in Appendix S1 (Section B) and also, subsequently, as a part of our sensitivity analyses.

MZ-twins, in addition to sharing the same nuclear and mitochondrial genes, also share the same intra-uterine *(IU)* and similar childhood *(CH)* environments. We further assume (Appendix S1; Section A; Assumption A2) that, of these, the *(IU)* environment has a far greater impact on the development of MS than does the shared *(CH)* environment. Once again, for MS, the direct observational data supports the validity of this assumption. Thus, studies in adopted individuals, in siblings and half-siblings raised together or apart, in conjugal couples, and in brothers and sisters of different birth order have generally indicated that MS-risk is unaffected by the *(CH)* micro-environment [4]–[7], [9], [10], [19], [20]. Regardless, however, the shared environmental experiences of MZ-twins, above and beyond the effect of the shared *(CH)* environment of siblings, potentially, could increase the proband-wise concordance rate [30]. As a result, the directly-observed MZ-twin concordance rates (Table 2) need to be adjusted to exclude the impact of these environmental similarities (Appendix S1; Section C; Prop. 1.4). These adjusted concordance rates, therefore, will reflect only the impact of an individual sharing an identical genotype *(IG)* with their MZ-twin who has MS. Two adjustments are envisioned. The first represents the total penetrance of the complex genetic trait (including both purely environmental and genetic cases) is referred to as:
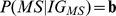
This penetrance (**b**) is estimated to be 0.134 (XPATH ERROR: unknown variable "string".; Section C; Prop. 1.4). The second adjusted rate represents the penetrance of the complex genetic trait exclusively in the set of genetically susceptible individuals and is referred to as:
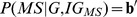
From Prop. (1.6) of Appendix S1 (Section C):
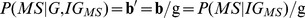
(4)

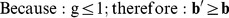
(5)


### Estimating proportion of population that is genetically susceptible to getting MS

We can partition *(G)* into two mutually-exclusive subsets (*G1* and *G2*) based on their disease-penetrance. The subset *(G1)* is defined as the high-penetrance subgroup (i.e., consisting of genotypes with a penetrance-value as high or higher than the expected penetrance for the entire susceptible-population) whereas *(G2)* is defined as the low-penetrance subgroup (i.e., genotypes having a penetrance-value as low or lower than this expected penetrance). Genotypes with a penetrance-value exactly equal to the expectation are divided evenly (and randomly) between the *(G1)* and *(G2)* subsets (to ensure that the subsets are mutually-exclusive). We define the expected disease-penetrance of these different sets as:
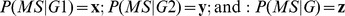
By these definitions: 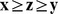


From Prop. (2.1) of Appendix S1 (Section C):
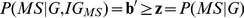
(6)So that, from Equations (2–6):
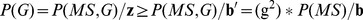
(7)As demonstrated below and in Prop. (5.2b) of Appendix S1 (Section C), we estimate that (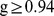). Therefore, using this estimate, together with the values presented in Table 2, yields the estimate of:
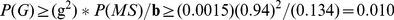
(8)This provides a lower-bound for the probability of being genetically susceptible to MS.

To provide an upper-bound for *P(G)*, we define three quantities (p, **a**, and **a′**) such that:
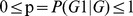


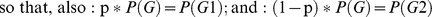
and, therefore, that:
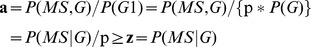


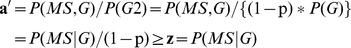
From Prop. (3.4) of Appendix S1 (Section C):
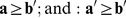
(9)Therefore: 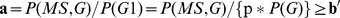


so that, with rearrangement: 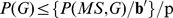



Moreover, because: 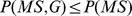; and, by Equation (5): 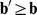


Therefore:
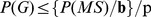
(10)Similarly: 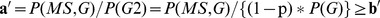



so that, with rearrangement: 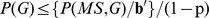



And, therefore, also:
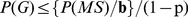
(11)Because one of the following three statements must be true:
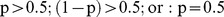
Therefore, making only Assumptions (A2–A4) from Appendix S1 (Section A), the Equations (10)&(11), place two simultaneous constraints on *P(G)* and, together with Equations (6–8), require that:
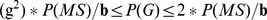
(12)which can be rewritten equivalently as:
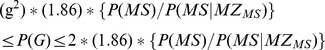
Because the quantities *P(MS)* and *P(MS|M
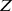
_MS_)* are directly observable parameters (Table 2), we can substitute, into Equation (12), the values of:
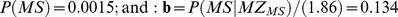
Doing this, together with Equation (8), yields the estimate of:
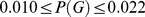
(13)Thus, making Assumptions (A1–A4) from Appendix S1 (Section A), no more than 2.2% of the general population is genetically susceptible to getting MS (Appendix S1; Section C; Prop. 4.2). A very similar range-estimate for *P(G)* is derived from epidemiological data obtained from different populations throughout North America and Europe (Table 8).

**Table 8:** Estimated prevalence (probability) of genetic susceptibility in different geographic regions.

	MSPrevalence ‡	MZ-TwinConcordance *	% Susceptible †
	*P(MS)*	*P(MS*|*MZ*_MS_)	*P(G)*
**North America**			
Canada [21]	68 – 248	25.3%	0.4 – 3.6%
Northern US [12]	100 – 160	31.4%	0.5 – 1.9%
Southern US [12]	22 – 112	17.4%	0.2 – 2.4%
**Europe**			
Finland [31]	52 – 93	46.2%	0.2 – 0.7%
Denmark [32]	110	24%	0.7 – 1.7%
British Isles [13]	74 – 193	40.0%	0.3 – 1.8%
France [11]	32 – 65	11.1%	0.5 – 2.2%
Sardinia [16]	144 – 152	22.2%	1.1 – 2.5%
Italy [16]	38 – 90	14.5%	0.4 – 2.3%

‡ Per 10^5^ population. The prevalence of MS in each region is from data provided in Reference [33]. A range is given because, often, a range of estimates are available for a particular region.

* Studies [11]–[13] reported pair-wise MZ-twin concordance-rates, which have been converted into proband-wise rates assuming a random sampling of twin-pairs [30]. Study [12], however, reported no double ascertainment of twin-pairs and, therefore, almost certainly violates this assumption [30].

† *P(G)* calculated according to Eq. (**6**); Prop. (4.2a); Appendix S1 (Section C); that: 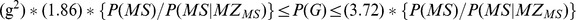

This equation assumes that (g) for each geographic region is: 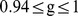; Appendix S1; Section C; Prop. (5.2b)

Moreover, as noted in Prop. (4.2b), Eq. (**6**) also assumes that: 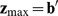

A narrower range-estimate could be provided by Eq. (**13**); Prop. (4.2b) of Appendix S1 (Section C) . However, regardless of which range-estimate for (**z**_max_) is used, this only impacts the lower-bound estimates for *P(G)*. The upper-bound estimates remain the same.

### Estimating the proportion of MS patients who are genetically susceptible

In order to estimate the quantity (g), we can partition the general population into two subsets, *(Gx+)* and *(Gx−)*, based on the presence or absence of some genetic factor *(Gx)* related to susceptibility (Appendix S1; Section C; Props. 1.7&5.2a). In Table 1, as before with (**b**&**b′**), we define two adjusted penetrance-values for each subset, either based on all cases (**s**&**t**) or based on just the genetic cases (**s′**&**t′**). Additionally, as in Table 1, we define two sets of parameters (A_0_&A) and (g_1_&g_2_) such that:
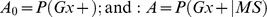
and: 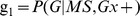; and: 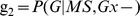


Using, in part, the result of Equation (13) and, as demonstrated in Prop. (5.1) of Appendix S1 (Section C), four relationships must hold:
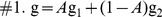
(14)

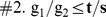
(15)

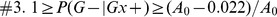


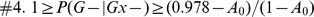
From this, we define the parameter (B) such that:
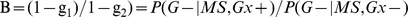
which, from Prop. (5.2a) of Appendix S1 (Section C), is equivalent to:
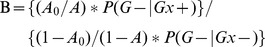
(16)

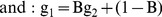
(17)Using Equations (13)&(16) together with the above relationships (#3)&(#4), yields:
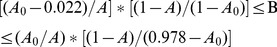
(18)Because the quantities A, A_0_, **t**, and **s** are either directly observable (or derived-directly from observations) for any partition of *(G)*, therefore, we can use Equations (14–18) to estimate the unknown values of B, g, g_1_, and g_2_ using experimental-data (Prop. 5.2a; Appendix S1; Section C).

In MS, from the gender-partition, our estimate is: 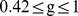


and, from the HLA-partition, our estimate is: 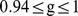


Notably: 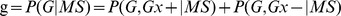


Therefore, the estimated value of (g) will be the same regardless of which partition is chosen for its estimation (as long as *Gx* is associated with susceptibility – see Props. (1.7)&(5.2a) of Appendix S1 (Section C). Thus, in order to satisfy both the gender and the HLA estimates of (g), we conclude that, for MS, more than 94% of the cases occur in genetically susceptible individuals (Prop. 5.2b; Appendix S1; Section C). The conclusion that the proportion of genetically susceptible cases is very high, is also reached in Prop. (5.3) of Appendix S1 (Section C) using the population-based epidemiological data reported from Finland [31], [32].

### HLA-DRB1 Subgroup differences in disease-penetrance

There are two possible mechanisms whereby *Gx+* individuals could be enriched in the MS-population compared to the general population (Appendix S1; Sections C&D; Props. (1.7)&(6). These are:

Mechanism (1) 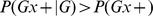


or, equivalently: 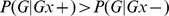 {a difference in “allelic” frequency}

Mechanism (2) 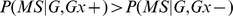 {a difference in penetrance}

In addition, there are three (potential) enrichment-stages for *(Gx+)*, which take place in MZ-twins (Appendix S1; Section D; Prop. 6.1a). The first stage occurs when moving from the set *(Gx+)* to the set *(Gx+,G)*; the second occurs when moving from the set *(Gx+,G)* to the set *(Gx+,G,MS)*, or equivalently to the set *(Gx+,G,IG_MS_)*; and the third occurs when moving from the set *(Gx+,G,IG_MS_)* to the set *(Gx+,G,MS,IG_MS_)*. As discussed in Prop. (6.1a) of Appendix S1 (Section D), the first stage can only involve Mechanism (1) whereas, the second and third stages can only involve Mechanism (2).

Moreover, the ratio (**s′**/**b′**) provides an estimate of the extent to which these two mechanisms operate (Appendix S1; Section D; Props. 6.1&6.2). If only Mechanism (1) is responsible for the enrichment, then:
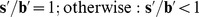
Unfortunately, the quantities (**s′**) and (**b′**), unlike the quantities (**s**) and (**b**), are not derived from direct-observations. However, from Props. (5.1&5.2b) of Appendix S1 (Section C) for MS and for the HLA partition, it is the case that:
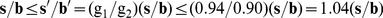
(19)So that, for the HLA-partition, this yields: 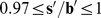


and, therefore, it follows that Mechanism (1) accounts almost entirely for the enrichment of DRB1*1501 in an MS-population. Consequently, from Props. (2.3b,6.3b,&7.1a) of Appendix S1 (Sections C&D), the following relationships can be demonstrated:
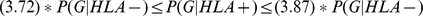


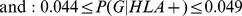


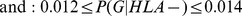


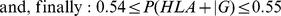


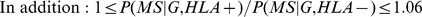


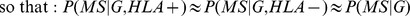



Consequently, despite the importance of DRB1*1501 for genetic-susceptibility, only a very small fraction of carriers (<5%) are even genetically susceptible to getting MS. Also, the conclusion that, for HLA-status, Mechanism (1) operates almost exclusively is supported by the observed lack of any continued HLA-enrichment in moving from the general population, to the *(MS)* population, and then to the *(MS, M
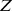
_MS_)* population. Thus, from Tables 2 and 5:
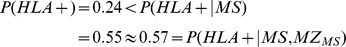



The enrichment of homozygous DRB1*1501 *(2HB+)* is approximately 3-fold greater than for single-allele carrier-status (Prop. 6.3c; Appendix S1; Section D). Nevertheless, even in this circumstance, Mechanism (1) still seems to account (almost entirely) for the enrichment of 2HB+ (Prop. 6.3c; Appendix S1; Section D). This suggests that neither heterozygous nor homozygous carrier-status affects disease-penetrance (Appendix S1; Sections C&D; Props. 5.3a,5.3c,6.3b,&6.3c).

In addition, it is a notable fact that all of these MS-populations seem to be at or near the Hardy-Weinberg equilibrium (HWE) state (Tables 3 and 4). From Prop. (6.4b) of Appendix S1 (Section D), this observation indicates that the relative normalized selection pressure for two DRB1*1501 alleles (w^2^) is equal to the square of that for one allele (w>1). In this sense the two DRB1*1501 alleles are said to be independently selected. Thus, the weighting for the homozygous-lack, and for the heterozygous- and homozygous-presence, of the risk allele is geometric (1,w,w^2^). This is analogous to the joint probability of two events being the product of the individual probabilities; and it contrasts to the weighting scheme for recessive and dominant traits (assuming a non-zero risk for non-carriers), which would be (1,1,w) and (1,w,w), respectively. This suggests the possibility that each DRB1*1501 allele contributes equally to the total number of susceptibility alleles required (Appendix S1; Section B & Section E; Prop. 6.4b). For example, if susceptible “non-DRB1*1501” genotypes have (on average) ten susceptibility alleles, perhaps susceptible genotypes with one DRB1*1501 allele have only nine, whereas genotypes with two such alleles might have only eight [27].

Finally, susceptible women (compared to susceptible men) have a higher mean allelic frequency (MAF) for the DRB1*1501 allele, a difference which is consistently reflected in MS-populations (Tables 2,3,4 & Appendix S1; Section E; Prop. 6.4d). This imbalance is due primarily to a gender difference in the composition of the subset *(G)* of susceptible individuals (; Section E; Prop. 6.4d).

As noted above, one of the features of susceptible genotypes that include the DRB1*1501 allele seems to be that they have a (slightly) reduced number of susceptibility alleles present (on average) compared to other susceptible genotypes [27]. In this circumstance, the observed MAF gender-difference would be expected if this reduction (for DRB1*1501 genotypes) were somewhat greater in women than in men.

### Gender Subgroup differences in disease-penetrance

**Figure 1:**
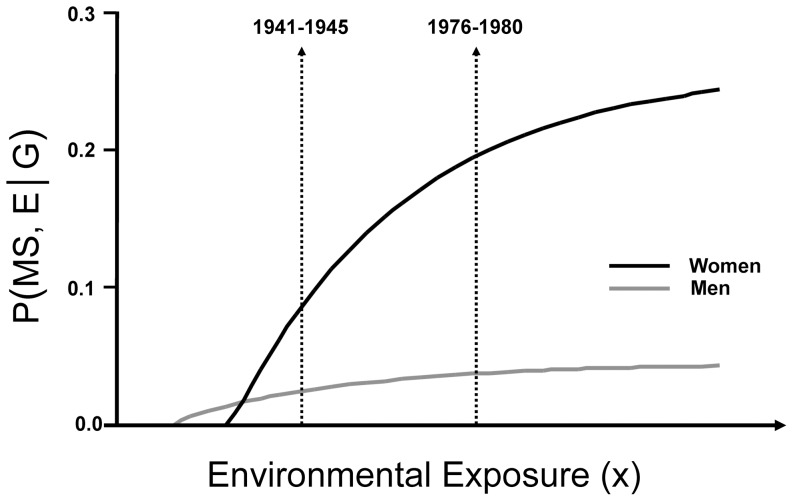
Response-curves for developing MS in susceptible men *(M)* and women *(F)* to an increasing likelihood of a “sufficient” environmental exposure *(E)*. Proportionate hazard is assumed for the two genders (see Appendix S1; Section F). The probability of developing MS – *P(MS, E|G)* – is shown on y-axis and the transformed environmental exposure (x) is shown on the x-axis {NB: (x) increases with *(E)* but not necessarily linearly – see Appendix S1; Section F}. The maximum y-axis excursions have been set to the high-point of the predicted ranges for *P(MS|G, E, M)* & *P(MS|G, E, F)* given by Eqs. (**14**) & (**16**) – Appendix S1; Section E; Prop. (7.1c). The proportionality constants, (C) and (r), are taken to be 0.5 and 1, respectively. One “environmental unit” has been defined arbitrarily as the change in the level of a sufficient environmental exposure *(E)*, which has taken place between the time-periods of (1941–1945) and (1976–1980). Based on the increase in the gender-ratio of MS patients over this interval, together with the proband-wise MZ-twin concordance-rates for MS in men and women from Canada [15], [21], two conclusions follow directly. First, there has been more than a 32% increase in the prevalence of MS in Canada between these two time-periods and second, compared to women, men begin to develop MS at a lower level of environmental exposure (x) or they have a greater hazard-rate (see Appendix S1; Section F). In either case, women are more responsive to the environmental changes that are taking place than men (regardless of what these changes actually are). Presumably, this explains the observation that the prevalence of MS is increasing, especially among women [4]. Each of these conclusions is apparent in the Figure. The response curve for men starts at a lower value of (x) than women but their response curve is almost at its plateau in (1941–1945). By contrast, women are nowhere near their (much higher) plateau in (1941–1945) and, compared to men, have a much steeper rise of *P(MS|G, E)* in response to the environmental changes, which have taken place during the interval. {NB: the x-axis is **not** a time-axis. The x-axis represents increasing levels of environmental exposure (x) from whatever cause and over whatever period of time it has taken place.}

For MS and for gender, from Prop. (6.1c) of Appendix S1 (Section D), we can also write Equation (19) as:
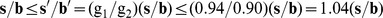
so that, from Table 6, for the gender partition, this becomes: 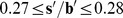



It turns out that this implies (Appendix S1; Sections D&E; Props. 6.3b&7.1a) that both Mechanisms (1) and (2) operate and, thus, that:
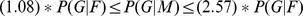


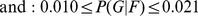


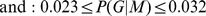


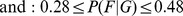


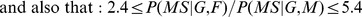
Thus, men are more likely to be genetically susceptible to MS compared with women although susceptible men are less likely to get MS than susceptible women. This same conclusion was suggested earlier [4] and, in fact, the actual response-curves demonstrating the greater responsiveness of women to increasing environmental exposures (and, thus, the greater penetrance of MS in women) can also be derived quantitatively (assuming proportionate hazard for MS in men and women) from the same epidemiological data (Figure 1; & Appendix S1; Section F). Notably, increasing the likelihood of a sufficient environmental exposure *(E)* in susceptible individuals, *P(E|G)*, does not increase the likelihood of MS developing beyond 28% in women and beyond 6% in men (Figure 1; & ; Section F). This must be due to the fact that certain genetic backgrounds are only (or more) responsive to certain sufficient environmental experiences (Appendix S1; Section F). For example, even if all susceptible genotypes required a particular environmental stimulus (e.g., vitamin D deficiency), some susceptible genotypes might require a longer duration or a greater intensity of exposure to produce MS than others (Appendix S1; Section F). Also, assuming a proportional hazard for men and women, susceptible men (compared to susceptible women) must have a lower threshold, a greater hazard-rate, or both in response to the environmental factors involved in MS pathogenesis (Figure 1 & Appendix S1; Section F).

In addition, the greater penetrance of MS in susceptible women is also reflected by the continued enrichment of women in going from the general population to the *(MS)* population and then to *(MS, M
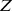
_MS_)* population. Thus, from Tables (2,3,4,5), and as discussed in Prop. (5.3) of Appendix S1 (Section C):
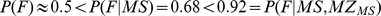
As a result, we conclude that gender has a marked impact on both disease penetrance and disease susceptibility (Appendix S1; Sections C&D; Props. 5.3b,5.3c,&6.3a).

### Estimating the penetrance of susceptible and non-susceptible genotypes

Rearranging Equations (4) and (11) yields:
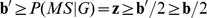
From Prop. (5.2b) and substituting into this equation the value of: 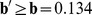
yields the estimate of:
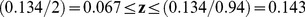
(20)


This range-estimate can be narrowed considerably (see Appendix S1; Section E; Prop. 7.1a) by recognizing that:
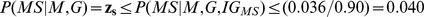


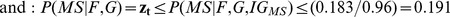


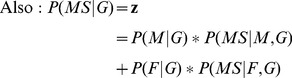
(21)Therefore, the predicted ranges from Prop. (6.2b) of Appendix S1 (Section D) lead to the boundaries:

lower-bound: 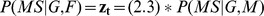; and: 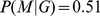


upper-bound: 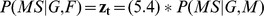; and: 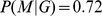


From Prop. (7.1) of Appendix S1 (Section E), substituting these values into Equation (21) yields the boundary estimates of:
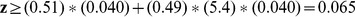
(22)

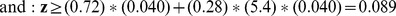
However, the lower-boundary of Equation (22) is slightly inconsistent with the most straight-forward lower bound condition that: 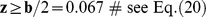


For MS, obviously, this discrepancy is quite minor. In other disease states, by contrast, it may be greater. Therefore, we provide a method for making the Equation (20) & (22) estimates “coherent” with each other (Appendix S1; Section E; Prop. 7.1a). For MS, solution of the two simultaneous equations yields the minimally modified lower boundary estimates of:
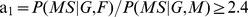
(23)

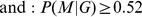
(24)so that:
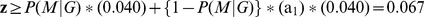
And, consequently, this yields the revised range-estimate for (**z**) of:
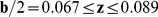
(25)From Equation (25), it also follows (Appendix S1; Section E; Prop. 7.1) that:
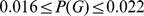


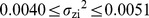


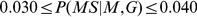
(26)

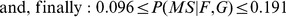
(27)Also, from Props. (4.2&5.2b) of Appendix S1 (Section C): 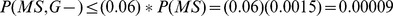


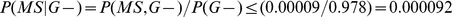


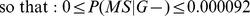


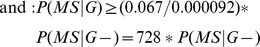



### Estimating the proportion of “purely genetic” MS

Because “purely genetic” MS is defined to be independent of the environment (see Appendix S1; Section B), its penetrance is expected to very high (i.e., near unity). Thus, we anticipate both that:
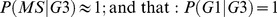
(28)If these conditions were not met, it would raise the question of what factors determined the lower penetrance. If these factors were potentially identifiable and non-hereditary, then they would constitute environmental events and, thus, these genotypes would be in *(G0)* and not in *(G3)*. Although a purely stochastic mechanism might lower the penetrance somewhat, this seems unlikely to reduce the penetrance markedly.

As shown in Prop. (7.2) of Appendix S1 (Section E), even if we make the extreme assumptions that:
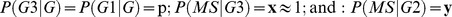
and assume that the variances of the of the (**x_i_**) and (**y_i_**) terms are zero;

and, finally assume that all values: 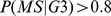; satisfy the conditions of Equation (28);

then, even in these extreme conditions, we still estimate that:
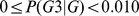



However, these conditions seem too extreme for any actual distribution and, notably, less extreme assumptions lead to even smaller estimates for *P(G3|G)*. Therefore, this derived upper limit for the range of *P(G3|G)* is, almost certainly, too large.

And, consequently, it must be that: 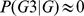


And, thus, for all practical purposes, “purely genetic” MS does not exist.

### Sensitivity considerations

Naturally, all of the range-estimates provided here are dependent upon the accuracy of the underlying epidemiological data in Tables 2,3,4,5,6. To illustrate this, we will use our Equation (13) estimate for P(G) where we estimated that:
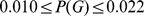
For example, if we consider the prevalence of MS in the 45–55 year age-range (e.g., Appendix S1; Section B) to be a better estimator of *P(MS)* then, potentially, the estimate of (0.0015) used here could double [29]. In this case {i.e., if: 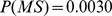; and all else is equal}, then our Equation (13) range-estimate for *P(G)* would be increased to:
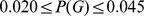
By contrast, even though the estimate for (B) changes slightly using this upper bound, the estimate for (g) derived from the HLA partition in Prop. (5.2a2) of Appendix S1 (Section C), remains unchanged at:
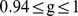
Similarly, if the proband-wise MZ-twin concordance in northern populations is 35% rather than the 25% used here [3], then this would lead to:
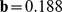
and our Equation (13) estimate would become: 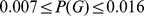



Also, if P(MS|S_MS_) is actually 3.5% instead of 2.9% then:
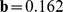
and the Equation (13) estimate would become: 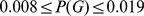



Finally, if all of these modifications were accepted, then the Equation (13) estimate would become:
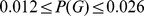
Thus, there is an additional level of uncertainty implicit in each of the range-estimates for the different parameters provided here.

### Assumption Violations

It is also important to consider what the impact might be if one or more of the Assumptions underlying the model were to be violated (Appendix S1; Section A). The most basic assumption of the model is that the twin populations are “representative” of the general population (see Assumptions A5&A6; Appendix S1; Section A). This assumption is critical and were it to be violated, the entire model would be invalid. Fortunately, as noted earlier, the direct observational data in MS support the validity of this assumption (e.g., [21]). Moreover, this assumption also underlies the “classical twin study” approach that has been used (and validated) for decades to elucidate the genetic and environmental bases of many human illnesses (e.g., [2]).

The second critical assumption of the model is that the *(CH)* micro-environment does not contribute to disease occurrence (Appendix S1; Section A; Assumption A2). Fortunately, as noted earlier, there is considerable observational data in MS (from numerous studies in adopted individuals, in siblings and half-siblings raised together or apart, in conjugal couples, and in brothers and sisters of different birth order) to support the notion that MS-risk is not impacted by the *(CH)* environment [4]–[7], [9], [10], [19], [20]. Nevertheless, if this assumption were to be violated, it would have a major impact on our ultimate conclusions.

For example, in Parkinson's disease *(PD)*, it has also been observed that siblings of a PD-proband carry a significantly greater risk of disease compared to unrelated controls (33). However, by contrast to MS, the MZ-twins of a PD-proband seem not to be at greater risk compared to DZ-twins, especially if the onset of illness is over age 50 [34]. In such a circumstance, the lack of any difference between the MZ-risk and DZ-risk, most likely reflects the fact that:
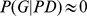
and, thus, that genetics are only minimally (or not) involved in disease pathogenesis. In this case, the increased-risk in siblings is presumably due to the similar *(CH)* environment, which siblings share, and, therefore that:
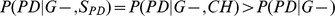
Even if, unlike the situation in PD, both the genetic make-up and the *(CH)* environment contribute to the increased disease *(D)* risk, then it would still be the case that:
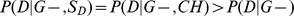
In this circumstance, however, the relationship between {*P(D|G−, CH)*} and {*P(D|G, CH)*} cannot be deduced. Therefore, this violation would invalidate the conclusion that:
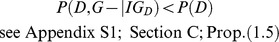
which would invalidate the further conclusion that: 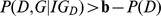



This, in turn, would invalidate the conclusion that:
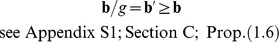
which would invalidate most of the Prop. 4&5 conclusions (Appendix S1; Section C).

Despite these consequences, however, a violation of Assumption (A2) would not be fatal to the model. Rather, it would mean that the model would need minor revision and that the *(CH)* impact would need to be estimated from experimental data, for example, by studying siblings raised separately or adopted children raised together with an MS-proband.

Assumption (A4); Appendix S1 (Section A), is crucial to conclusions about the relative prevalence of genetic susceptibility in the *(Gx+)* and the *(Gx−)* subsets. For example, for the gender partition (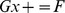), from Table 2 & Prop. (1.4b) of Appendix S1 (Section C), it seems that:
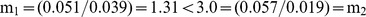
If these experimental observations are correct, then the impact of this violation would be that the true separation between men and women in the percentage of genetically susceptible individuals (Appendix S1; Sections C & D; Props. 1.4b&6.2a) would be underestimated. Naturally, the impact of the opposite violation (i.e., where: 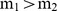), would be to overestimate this separation. However, from the available data, this seems not to be the case.

Other assumption violations would, in general only impact the specific propositions involved. Each of these assumptions, and the propositions they impact, are listed in Appendix S1 (Section A).

## Discussion

Both the mathematical model and the data presented here suggest that detailed study of MZ- and DZ-twin concordance data, combined with general epidemiological information regarding the disease from the same population as the twin data, are capable of providing quantitative estimates for many parameters associated with disease pathogenesis, which can't be directly-observed or easily measured. Thus, making only a few very simple (and quite plausible) assumptions about the genetic make-up of MZ- and DZ-twins, quantities such as *P(G)*, *P(E)*, *P(G3|G)*, *P(G|MS)*, *P(MS|G)*, *P(MS|G−)*, *P(F|G)*, *P(MS|E,G,F)*, *P(MS|E,G,M)*, and *(σ_zi_^2^)* can be estimated from directly observable data (Table 7). Also this model can provide these parameter estimates for other complex genetic disorders (e.g., Table 9). Finally, the model can provide insight to the mechanisms of disease pathogenesis. For example, in MS, this analysis indicates that the basis for the association of DRB1*1501 with MS is due to the fact that persons who carry this allele have a greater likelihood of being genetically susceptible compared to persons who lack this allele. In addition, each DRB1*1501 allele seem to affect susceptibility independently. By contrast, carrier status does not seem to affect the likelihood of developing the disease in the susceptible population. Moreover, despite the strong association of DRB1*1501 with MS, the majority (∼59%) of genetically susceptible individuals are susceptible based on genotypes that do not include this allele and, indeed, for the 25% of these individuals who, nonetheless, still carry this allele, the presence of DRB1*1501 seems not to contribute to their susceptibility (Prop. 8.1; Appendix S1; Section E). In addition, among carriers of this allele, fewer than 5% are even susceptible to getting MS in the first place (Appendix S1; Section D; Prop. 6.3b).

**Table 9:** Estimated prevalence (probability) of genetic susceptibility in rheumatoid arthritis, ankylosing spondylitis, and systemic lupus erythematosus

	Prevalence ‡	MZ-TwinConcordance *	% Susceptible
	*P(D)*	*P(D|MZ_D_)*	*P(G)*
Rheumatoid Arthritis	1 – 2%	∼ 35%	5.7 – 11.4%
Ankylosing Spondylitis	0.4 – 4%	∼ 53%	1.4 – 15%
Systemic Lupus Erythematosus	∼ 0.025%	∼ 39%	∼ 0.13%

‡ The prevalence of diseases {*P(D)*} is from data provided in Reference [35].

* Studies [36]-[38] report pair-wise MZ-twin concordance-rates. These have been converted into proband-wise rates {*P(D|MZ_D_)*} assuming a random sampling of twin-pairs [30]. Also, the IU environment has been assumed to have no impact on the disease. A violation of either of these assumptions will make the estimate of *P(G)* too low.

In the case of gender, however, the disease association turns out to result from a combination of effects. Thus, despite men having a greater likelihood than women of being genetically susceptible, women who are susceptible are considerably more likely to develop the disease than susceptible men. Although, the distinction between men and women is (in some sense) genetic, the principal anatomic and physiological differences between genders are likely not to be linked to specific allelic variations but, rather, are almost certainly based on differences in the regulation of developmental programs that are shared by all same-sexed individuals. Because the observed gender differences in disease penetrance seem to be the result of an increased physiological responsiveness of women to common environmental events (see Appendix S1; Section F), therefore, the genetic basis of this particular influence is unlikely to be uncovered through approaches such as genome-wide association studies (GWAS). By contrast, the genetic basis for the gender-related differences in the likelihood of susceptibility could arise from either allelic or epigenetic differences between the sexes and might, potentially, be detected using GWAS or other genetic methods, particularly if men and women were to be analyzed separately. Alternatively, if the lower likelihood of susceptibility in women were due to an increase in the average number of susceptibility-genes necessary to produce susceptibility in women, this, also, would likely not be evident using a GWAS approach. Moreover, because of the huge number of anticipated susceptibility-genotypes (Appendix S1; Section B), few MS patients are likely to share the exact same combination of susceptibility genes. Therefore, as discussed in Appendix S1 (Section B), novel approaches to the analysis of these large datasets [26] are almost certainly going to be necessary in order to clarify the genetic underpinnings of MS.

These considerations also have implications for some of the gene-disease associations, which have been occasionally suggested in the literature. For example, recently, Gregory and co-workers, reported genetic evidence that implicated the single nucleotide polymorphism (SNP), rs1800693, as the variant within the TNFRSF1A gene, which is associated with MS-susceptibility by genome wide association studies [39]. This is the gene, which encodes tumor necrosis factor (TNF) receptor-1. These authors further suggested that this particular genetic variant was “causal” for MS-susceptibility by demonstrating that the MS risk-allele results in expression of a novel and soluble form of TNF receptor-1. The novel transcript produced by this mutation skips Exon 6 and results in the formation of a substantially truncated protein, which functions as a TNF-blocker [39]. However, despite the seeming plausibility of this proposed mechanism for MS-susceptibility associated with this SNP, the offered explanation is, at best, incomplete – a conclusion based solely on relationships derived for the proposed model. Thus, because, only a tiny fraction (≤2.2%) of the population is genetically susceptible to getting MS, and because the risk-allele frequency (MAF) for this “causative” SNP-variant is 40% [39], the maximum percentage of “risk-allele” carriers who could possibly be genetically susceptible is only 3.4% (2.2/64). Even if the risk were assumed to be carried exclusively by homozygotes for the risk-allele, this maximum percentage rises to just 13.8% (2.2/16). Consequently, this risk-allele, by itself, is insufficient to produce susceptibility – rather, it is only in combination with other susceptibility alleles that this particular variant can lead to genetic susceptibility to MS [27]. Moreover, the fact that many MS patients are not carriers (∼36%) is indicated by the small odds ratio (1.12) for the association of this risk-allele with MS [39]. In such circumstances, this particular SNP-variant can hardly be described as “causative” for MS-susceptibility.

In conclusion, the mathematical model for disease pathogenesis, here developed, is capable of providing considerable insight to the nature and basis of genetic susceptibility to chronic human diseases in different groups of individuals.

## Supporting Information

Appendix S1 (PDF)
