# Supplementary material for: The Genetic and Environmental Bases of Complex Human-Disease: Extending the Utility of Twin-Studies
Source: PLoS One. 2012 Dec 18;7(12):e47875. doi: 10.1371/journal.pone.0047875 (PMC3525648; doi:10.1371/journal.pone.0047875)
Supplement: Appendix S1 — (PDF) [file pone.0047875.s001.pdf]

Appendix S1

1. Model Definitions, Assumptions, & Supplemental Tables ……..…................ Section A

2. Conceptualizing Susceptibility and Disease-Risk ………………...............… Section B

3. Establishing Relationships and Estimating the Parameters (Props. 1-5) .... Section C

4. Impact of Gender and HLA DRB1*1501 (Prop. 6) ………....….........…....… Section D

5. Refining the Parameter Estimates (Props. 7-8) ....………..........…... Section E

6. Response to Environmental Events ....…………..….........… Section F

Large Red Rectangles above represent hyperlinks to Main sections of Appendix S1:

Left Mouse click to navigate within the document

Small Red Boxes within Document represent hyperlinks within Appendix S1.

Small Green Boxes within Appendix S1 represent hyperlinks back to the Main Text.

NB: Hyperlinked and fully referenced document (Main Text and Appendix S1) is available from the author upon request: douglas.goodin@ucsf.edu

Appendix S1; Section A

Model Definitions, Assumptions, & Supplemental Tables

1. Principal Model Definitions from Main Text ………………………….. Table S1

2. Additional Model Definitions; specifically for Propositions …….……. Table S2

3. Components of Genetic Susceptibility to Multiple Sclerosis (MS)…...… Table S3

4. Model Assumptions ……………………………………...…...…...… Table S4

Large Red Rectangles above represent hyperlinks to main parts of Section A

Small Red Boxes within Document represent hyperlinks within Appendix S1.

Small Green Boxes within Appendix S1 are hyperlinks back to Main Text

Navigate Back to Main Menu

Table S1. Principle Model Definitions; from the Main Text

P(MS)	=	The life-time probability of developing MS in the general population.<br>[equated to the prevalence of the disease]
$P(MS \mid IG_{MS})$	=	The conditional life-time probability of developing a MS, given that the person's MZ-twin has MS; adjusted to exclude the impact of twins sharing intra-uterine (IU) and childhood (CH) environments: $P(MS \mid IG_{MS})$ = **b**
(MZ_{MS}) , (DZ_{MS}) , (S_{MS})	=	Sets of persons with a monozygotic (MZ)-twin, a dizygotic (DZ)-twin, or a sibling (S) who either has or will develop MS.
(IU) , (CH)	=	Sets of persons who share, with an MS-proband, either the same intra-uterine (IU) or a similar childhood (CH) environment
(G) , (G–)	=	Sets of persons who either are (G) or are not (G–) genetically-susceptible to MS.<br>(G+) + (G–) = (P)
(G1) , (G2)	=	Two mutually exclusive subsets of (G); one with high-penetrance genotypes (G1) and the other with low-penetrance genotypes (G2). (G1) + (G2) = (G)
(G0) , (G3)	=	Mutually exclusive sets of genetically-susceptible individuals who depend upon (G0) or don't depend upon (G3) environmental events to get MS: (G0) + (G3) = (G)
(FT) , (ST)	=	The sets of first (FT) or second (ST) twins of an MZ-twin pair
(Gx+) , (Gx–)	=	The set of persons who either possess (Gx+) or don't possess (Gx–) the particular genetic characteristic (Gx).
(HLA+) , (HLA–)	=	The set of persons who either carry (HLA+) or don't carry (HLA–) at least one HLA DRB1*1501 allele. (HLA+) = (2HB+) + (1HB+)
(1HB+) , (2HB+)	=	Sets of persons who carry one (1HB+) or two (2HB+) copies of the DRB1*1501 allele.
(1HB–)	=	The set of persons who carry one copy of a non-DRB1*1501 allele<br>P(1HB–, 1HB–) = P(HLA–) ; P(1HB+, 1HB–) = P(1HB+) = P(1HB–)
(F) , (M)	=	Sets consisting of either women (F) or men (M)
**a** , **a'**	=	P(MS, G) / P(G1) = **a** ; and: P(MS, G) / P(G2) = **a'**
**b** , **b'**	=	$P(MS \mid IG_{MS})$ = **b** ; and: $P(MS \mid G, IG_{MS})$ = **b'**
**x** , **y**, **z**	=	$P(MS \mid G1)$ = **x** ; $P(MS \mid G2)$ = **y** ; and: $P(MS \mid G)$ = **z**
$\mathbf{z_t}$, $\mathbf{z_s}$	=	$P(MS \mid G, Gx+)$ = $\mathbf{z_t}$; and: $P(MS \mid G, Gx–)$ = $\mathbf{z_s}$
**t** , **t'**	=	$P(MS \mid Gx+, IG_{MS})$ = **t** ; and: $P(MS \mid G, Gx+, IG_{MS})$ = **t'**
**s** , **s'**	=	$P(MS \mid Gx–, IG_{MS})$ = **s** ; and: $P(MS \mid G, Gx–, IG_{MS})$ = **s'**
p	=	$P(G1 \mid G)$
g , g_1 , g_2	=	$P(G \mid MS)$ = g ; $P(G \mid Gx+, MS)$ = g_1 ; and: $P(G \mid Gx–, MS)$ = g_2
A_0 , A , A_1	=	P(Gx+) = A_0 ; $P(Gx+ \mid MS)$ = A ; and : $P(Gx+ \mid MS, IG_{MS})$ = A_1
MAF, HWE	=	Mean allelic frequency (MAF) ; Hardy-Weinberg Equilibrium (HWE)

Table S2. Additional Model Definitions; specifically for Propositions

(E) , (E–)	=	Sets of environmental exposures that either are (E) or are not (E–) sufficient to produce MS (environmentally) in an individual (see Section B)
(E0) , (E3)	=	Mutually exclusive sets of environmental exposures that depend upon (E0) or don't depend upon (E3) genetic susceptibility to produce MS: (E0) + (E3) = (E) (see Section B)
(S+) , (S–)	=	Sets of persons with susceptible genetic combinations that either do (S+) or do not (S–) include the DRB1*1501 allele (see Section B & Section E; Prop. 8)
(P) , (P1) , (P2) , (P3)	=	Sets of: all individuals in the population (P) ; those aged <15 years (P1) ; those aged 15–45 years (P2) ; and those aged > 45 years (P3)
**x'**, **y'**	=	$P(MS \mid G1, IG_{MS}) = \mathbf{x'}$; and: $P(MS \mid G2, IG_{MS}) = \mathbf{y'}$
m	=	$P(MS \mid DZ_{MS}) / P(MS \mid S_{MS})$
m_1	=	$P(MS \mid Gx+, DZ_{MS}) / P(MS \mid Gx+, S_{MS})$
m_2	=	$P(MS \mid Gx–, DZ_{MS}) / P(MS \mid Gx–, S_{MS})$
q	=	$P(G1 \mid G, MS) = P(G1 \mid G, MZ_{MS}) = P(G1 \mid G, IG_{MS})$
q'	=	$[P(MS \mid G, IG_{MS}) – P(MS \mid G2)] / [P(MS \mid G1) – P(MS \mid G2)]$: $\{q' = (\mathbf{b'} – \mathbf{y}) / (\mathbf{x} – \mathbf{y})\}$
G_i , G_j , G_k	=	Individual susceptibility genotypes: within the general population (i); within the (Gx+)-population (j); and within the (Gx–)-population (k)
$\mathbf{z}_{min}$, $\mathbf{z}_{max}$	=	Minimum ($\mathbf{z}_{min}$) and maximum ($\mathbf{z}_{max}$) of the range-estimate for ($\mathbf{z}$): $\{\mathbf{z}_{min} \leq \mathbf{z} \leq \mathbf{z}_{max}\}$
$\mathbf{z_i}$, $\mathbf{z_j}$, $\mathbf{z_k}$	=	Penetrance of the (i^{th}), (j^{th}), and (k^{th}) susceptibility genotype
σ_{zi}^2, σ_{zj}^2, σ_{zk}^2	=	Variance of the Penetrance distributions:<br>$\{Var(\mathbf{z_i}) = \sigma_{zi}^2\}$; $\{Var(\mathbf{z_j}) = \sigma_{zj}^2\}$; and: $\{Var(\mathbf{z_k}) = \sigma_{zk}^2\}$
n_b , n_t , n_s	=	Total number of susceptible genotypes: in the (G) subset (n_b) ; in the (G, Gx+) subset (n_t) ; and in the (G, Gx–) subset (n_s).
g_{01} , g_{02}	=	$P(G \mid Gx+) = g_{01}$; and: $P(G \mid Gx–) = g_{02}$
R_0 , R , R_1	=	$P(Gx+ \mid G) = R_0$; $P(Gx+ \mid G, MS) = R$; and: $P(Gx+ \mid MS, G, IG_{MS}) = R_1$
w_p , w_q , w_{pq}	=	normalized fitness levels of different population genotypes ; (see Prop. 6.4; Section E)
w	=	relative normalized fitness level ; $w = (w_p / w_q)^{½} > 1$; (see Prop. 6.4; Section E)
λ_w , λ_m , λ	=	Exposure threshold in women (λ_w), men (λ_m), and the threshold difference: ($\lambda = \lambda_w – \lambda_m$)
u , x	=	Actual (u) and transformed (x) environmental exposure levels for the susceptible population
**c** , **d**	=	Maximum probability of MS in genetically susceptible men (**c**) and women (**d**).
h(u) , g(u)	=	hazard-functions for developing MS in susceptible men {h(u)} and women {g(u)}
C , r	=	Proportionality constants for disease-prevalence and hazard. (see Section F)
Zm , Zw	=	probability of MS and a sufficient environmental exposure (E) in susceptible men (Zm) and women (Zw): $Zm = P(MS, E \mid G, M)$; and: $Zw = P(MS, E \mid G, F)$

Table S3. Components of Genetic Susceptibility to Multiple Sclerosis (MS)

Breakdown of P(MS) based on a Simple Susceptibility Scheme

	**E**	**E–**	
**G**	P(MS, G, E)	P(MS, G, E–)	P(MS, G)
**G–**	P(MS, G–, E)	P(MS, G–, E–)	P(MS, G–)
	P(MS, E)	P(MS, E–)	**P(MS)**

Breakdown of P(MS) based on a slightly more Complex Susceptibility Scheme

	**E3**	**E0**	**E–**	
**G3**	P(MS, G3, E3)	P(MS, G3, E0)	P(MS, G3, E–)	P(MS, G3)
**G0**	P(MS, G0, E3)	P(MS, G0, E0)	P(MS, G0, E–)	P(MS, G0)
**G–**	P(MS, G–, E3)	P(MS, G–, E0)	P(MS, G–, E–)	P(MS, G–)
	P(MS, E3)	P(MS, E0)	P(MS, E–)	**P(MS)**

Definitions (See also Section B & Tables S1 and S2; Section A)

G = Set of all susceptible genotypes

G0 = Set of susceptible genotypes that depend upon a susceptible environment

G3 = Set of susceptible genotypes that are independent of environment (i.e., purely genetic)

G– = Set of non-susceptible genotypes – i.e., those with the smallest penetrance: P(MS | G–)

E = Set of all susceptible environments

E0 = Set of susceptible environments that depend upon a susceptible genotype

E3 = Set of susceptible environments that are independent of genotype (i.e., purely environmental)

E– = Set of non-susceptible environments – i.e., those with the smallest penetrance: P(MS | E–)

Table S4. Model Assumptions

<u>Assumptions applicable to all propositions:</u>

A1. P(MS) is approximately equal to the prevalence of MS in the population (see Section B)

A2. $P(MS \mid G-, S_{MS}) = P(MS \mid G-, CH) = P(MS \mid G-, IG_{MS}) = P(MS \mid G-)$

A5. The genetic composition of the sets (MS), (IG_{MS}), and (MZ_{MS}) is the same.

Also: $P(MS \mid FT) = P(MS \mid ST) = P(MZ_{MS}) = P(IG_{MS}) = P(MS)$

i.e., the probability of being a 1st or 2nd MZ-twin is independent of MS-status

Therefore, also, for the ith susceptibility genotype (G_i) within (G): $P(G_i \mid IG_{MS}) = P(G_i \mid MZ_{MS}) = P(G_i \mid MS)$

Consequently: $P(G \mid IG_{MS}) = P(G \mid MZ_{MS}) = P(G \mid MS) = g$

Finally, also: $P(Gx+ \mid IG_{MS}) = P(Gx+ \mid MZ_{MS})$; and: $P(Gx+ \mid G, IG_{MS}) = P(Gx+ \mid G, MZ_{MS})$

A6. The genetic composition of the sets (G, FT), (G, ST), and (G) is the same

Also: $P(G \mid FT) = P(G \mid ST) = P(G)$

i.e., the probability of being a 1st or 2nd MZ-twin is independent of (G) status

<u>Assumptions limited to specific propositions:</u>

A3. For Props. (1.3) & (5.3), we assume that the observed increased risk of MS from the IU environment (see observed relationship #5; Prop. 1) applies to both the (G) and the (G–) subsets (although not necessarily equally to each). Therefore, we assume that:

$P(MS, G \mid DZ_{MS}) \geq P(MS, G \mid S_{MS})$ and: $P(MS, G- \mid DZ_{MS}) \geq P(MS, G- \mid S_{MS})$

A4. For Props. (1.4b, 1.5b, 5 & 6), we assume that:

$$m_1 = P(MS \mid Gx+, DZ_{MS}) / P(MS \mid Gx+, S_{MS}) = P(MS \mid Gx-, DZ_{MS}) / P(MS \mid Gx-, S_{MS}) = m_2$$

A7. Given our convention that: $P(MS \mid G, Gx+) = \mathbf{z_t} \geq \mathbf{z_s} = P(MS \mid G, Gx-)$

For Props. (5 & 6), we assume that: $P(MS \mid G, Gx+, IG_{MS}) = \mathbf{t'} \geq \mathbf{s'} = P(MS \mid G, Gx-, IG_{MS})$

A8. For Prop. (7.2), we assume that: $(G3) \subset (G1)$; or, equivalently: $P(G1 \mid G3) = 1$

A9. For Prop. (8.1), we assume that: $P(HLA+ \mid S-) \approx P(HLA+)$

A10. For Section F, we assume that: $P(MS, E) \approx P(MS)$

A11. For Section F, we assume that the hazard for developing MS in susceptible men and women is proportional.

Appendix S1; Section B

Conceptualizing Susceptibility and Disease-Risk

The Nature of Genetic Susceptibility ………...……………..………… p. 1

The Impact of Environmental Factors ….………………..…………… p. 3

The Relationship of P(MS) to Disease Prevalence ...……………….…… p. 4

The Number and Uniqueness of Susceptible Genotypes …………..…. p. 6

Large Red Rectangles above represent hyperlinks to main parts of Section B

Small Red Boxes within Document represent hyperlinks within Appendix S1.

Small Green Boxes within Appendix S1 are hyperlinks back to Main Text

Navigate Back to Main Menu

1. The Nature of Genetic Susceptibility

We define disease-penetrance for any specific genotype (or, equivilently, any specific individual) as the conditional life-time probability of disease given that particular genotype. For MS, it has been established that the DRB1*1501 allele, located on the short arm of chromosome 6, is an MS susceptibility-allele.[21-26] The set of carriers of this allele (HLA+) and the set of non-carriers (HLA–) form a partition of the general population. Within the populations of North America and northern Europe, it has been consistently observed that:

$$P(HLA+ \mid MS) > P(HLA+)$$

and, thus, also: $P(HLA- \mid MS) < P(HLA-)$ # (HLA+) and (HLA–) form a partition

Rewriting, rearranging, and combining these two equations yields:

(1) $$P(MS \mid HLA+) > P(MS) > P(MS \mid HLA-)$$

Therefore, a direct consequence of these observations[21-26] is that some genotypes must have a higher penetrance (for MS) than others and, therefore, there must be at least one genotype, within the population, that has the smallest penetrance of any. The set of all genotyes that share this same smallest penetrance is defined as (G–) and its penetrance is P(MS | G–). Members of this set are referred to as being "genetically non-susceptible." Conversely, the set of all genotyes, which have a penetrance greater than this minimum, is defined as (G), and its members are referred to as being "genetically susceptible." Based on these observations and considerations, therefore, both (G) and (G–) must contain at least one member, they are mutually exclusive, and they partition the population (see Table S3; Section A). Also, the penetrance P(MS | G–) may (or may not) be zero, depending upon the prevalence of purely environmental MS (see Main Text).

In the Model,[27] it is supposed that there are some number of susceptibility loci (**n**) that harbor susceptibility alleles (i.e., specific DNA sequences – in either "coding" or "non-coding" genomic regions – which, alone or in certain combinations with other such alleles, increase the likelihood of MS compared to individuals who possess only non-susceptibility alleles at each locus). Each locus is assumed to be a chromosomal region that is independent of other loci, although a particular locus may harbor more than one susceptibility allele at a particualar region or more than one (linked) susceptibility region.[27] By definition, the set (G) includes all genetic combinations at these **n** susceptibility loci that lead to genetic-susceptibility. The term P(G) represents the probability that an individual in the general population is a member of this set. We can partition (G) into disjoint subsets (G_h),where every genetic-combination in the subset (G_h) has, within its collection of genotypes at the

different susceptibility-loci, at least one group of (h) loci, which are in a "susceptible state" and that, by themselves (i.e., as a combination), would result in susceptibility to MS.[27] In addition, no member of the subset (G_h) can have a group of fewer than (h) loci in a "susceptible state" that, by themselves, would lead to MS-susceptibility. The term "by themselves" indicates that a person having this particular combination of "susceptible states" at the (h) loci is susceptible to getting MS, regardless of the "allelic state" at any other genetic locus.[27] Each subset (G_h) can be further divided into two sub-subsets (S_h+) and (S_h–) based on whether the particular combination that defines membership in the (G_h) subset either does (S_h+) or does not (S_h–) include the DRB1*1501 allele. Thus, we can also define two subsets of G, (S+) and (S–), such that:

$$S+ = \sum_h (S_h+) \; ; \qquad \text{and:} \qquad S- = \sum_h (S_h-)$$

In this conceptualization, genetic-susceptibility is understood to be (quantitatively) binary – an individual is either genetically susceptible or they are not. Nevertheless, within (G), there may be a wide variation in the likelihood that MS will develop (i.e., in the penetrance of the different genotypes). As can be appreciated from Eq. (**1**), such a binary structure is a direct consequence of the fact that DRB1*1501 is an undisputed MS susceptibility-allele for MS.[21-26] Also, as a consequence of this, both the number of susceptibility alleles and the number of loci that harbor such alleles must be at least one. Presumably, there are many others but, in any case, the total number must also have an upper-bound (i.e., not every allele or locus in the genome can be a susceptibility allele or locus). As noted earlier, the combination of allelic states (genotype) at the different susceptibility loci that has the least likelihood of resulting in MS, together with all other combinations that share this minimum likelihood, constitute the set (G–). Any combination (genotype) that increases this likelihood (even by a miniscule amount) belongs, by definition, to the set (G). Thus, the sets (G) and (G–) are mutually exclusive and both are non-empty. Nevertheless, the set of susceptible individuals (G) could, at least theoretically, encompass virtually the entire population (i.e., all but one genotype) and the penetrance of different susceptible-genotypes could range from nearly zero to one.

The set (G) can also be partitioned into those genotypes that are sufficient to produce disease but do so more often, or exclusively, in "susceptible" environmental circumstances (G0), and those that are sufficient to produce the disease but do so independently of an individual's environmental experiences (G3). The subset (MS, G3) will be referred to as "purely genetic" MS (see Table S3; Section A).

2. The Impact of Environmental Factors

As with genotypes, environmental factors can be partitioned into those sets of environmental experiences that are sufficient to produce the disease environmentally (E), and those that are not (E–). The set (E–), again, is defined as being that environmental exposure, which is associated with the least penetrance of MS {i.e., $P(MS \mid E–)$} of any environmental experience.

Analogous to genotypes, it has been consistently observed that: $P(MS \mid DZ_{MS}) > P(MS \mid S_{MS})$

Because DZ-twins and siblings have the same genetic relationship to each other, this observed penetrance difference must be due to the different environmental experiences of DZ-twins compared to siblings. Therefore, at least one environmental experience must be associated with the least (and greatest) likelihood of developing MS. Therefore, the sets (E–) and (E) are also non-empty.

Also, analogous to "susceptible" genotypes, the subset of "susceptible" environmental exposures (E) can be partitioned into those environmental experiences that are sufficient to produce disease but do so more often, or exclusively, in "susceptible" genetic backgrounds (E0), and those that produce disease independently of the genetic background (E3). The subset (MS, E3) will be referred to as "purely environmental" MS (see Table S3; Section A).

Moreover, because environmental factors are not considered in Sections (C – E), the conclusions of these propositions do not depend upon specific environmental considerations. Rather, they are based on the expected environmental experience of the population as a whole (i.e., E plus E–). This is similar to the manner, in which the different environmental events (see Table S3; Section A) are distributed over the genotypic make-up of the whole population (i.e., G plus G–).

To begin, we note that: $P(MS, G) \geq 0.00141 >> 0.00009 \geq P(MS, G–) \approx 0$ # Props. (4.2d & 5.2b)

Because: $P(MS, G–, E–) \leq P(MS, G–) \approx 0$ # $(G–, E–) \subset (G–)$

(**2**) Therefore, we assume that: $P(MS, G–, E–) = 0$

Moreover, because: $P(MS \mid G) \leq 0.089$ # Eq. (**8**) ; Prop. (7.1a)

Less than 10% of genetically susceptible individuals will actually develop MS. This indicates that environmental factors play a key role of MS pathogenesis.

In addition, because: $P(G3 \mid G) \approx 0$ # Prop. (7.2)

therefore: $P(MS \mid G, E–) \approx P(MS \mid G0, E–)$

The term $P(MS \mid G0, E–)$ represents the penetrance of genotypes, which are defined by their dependence upon a "susceptible" environment to produce MS, but which occur in the setting where the individual actually experiences a "non-susceptible" environment.

For both of these reasons, we assume that: $P(MS \mid G, E–) \approx 0$

(**3**) This translates to the assumption that: $P(MS, G, E–) \approx 0$

And, thus, from Eq. (**2** & **3**): $P(MS) \approx P(MS, E)$ # Table S3; Section A

And, if: $P(E \mid G, M) \approx P(E \mid G, F)$ # Eqs. (**28** & **29**); Section F

The observation that: $P(MS \mid G, M) << P(MS \mid G, F) << 1$ # Prop. (6.2)

Indicates that: $P(MS \mid E, G, M) << P(MS \mid E, G, F) << 1$

If so, then it must be the case that some sufficient environmental exposures (possibly most) are not equally effective at producing MS in all susceptible individuals.

3. The Relationship of P(MS) to Disease Prevalence

By the definition provided in Table S1, P(MS) represents the life-time (longitudinal) probability that an individual from the general population (P) will develop MS. In making Assumption (A1), we are equating this probability with the (cross-sectional) probability $\{P(MS \mid P)\}$ that an individual from the general population has MS at some particular point in time. Because almost all MS cases begin (clinically) between the ages of 15 and 45 years,[3] therefore, using the 2010 United States census data (total resident population) as an approximation, we can divide the general population (P) into the three mutually exclusive age-band subsets (P1, P2, and P3), defined as:

$P1 = \{< 15 \text{ years}\}$; where: $P(P1) \approx 0.20$; and: $P(MS \mid P1) \approx 0$

(**4**) $P2 = \{15\text{-}45 \text{ years}\}$; where: $P(P2) \approx 0.41$; and: $P(MS \mid P2) = (a_3)*P(MS)$; $0 < a_3 < 1$

and: $P3 = \{> 45 \text{ years}\}$; where: $P(P3) \approx 0.39$; and: $P(MS \mid P3) \approx P(MS)$

Clearly, using these approximate probabilities (together with these conditional probabilities), if they have been assigned correctly for the population under consideration, then our Assumption (A1) that:

$$P(MS) \approx P(MS \mid P)$$

will yield an estimate for P(MS), which is too low. The estimate will be better if only the (P2) and (P3) subsets are included in the denominator and will be better still if only P(MS | P3) is considered.

However, it is also the case that, in any MS cohort, individuals will experience an excessive mortality (due to MS) compared an unaffected control population.[29] Therefore, an even better estimate would be derived from the prevalence in the cohort of the population restricted to ages 45-55 years, in which new incident cases are unlikely to occur [3] and where substantial early mortality from MS is unlikely to have yet happened.[28]

To get a sense for the possible magnitude of the underestimation, using these approximate probabilities above, then, from Eq. (**4**), we can calculate that:

If: $1 > a_3 \geq 0.5$; as seems likely with an average onset-age [3] for MS of: ~30 yrs

Then: $(1.3)*P(MS \mid P) < P(MS) \leq (1.7)*P(MS \mid P)$

Clearly, a similar underestimation will occur for the quantities $P(MS \mid P, MZ_{MS})$ and $P(MS \mid P, DZ_{MS})$; the estimates for which, again, rely on cross-sectional probabilities being substituted for longitudinal probabilities. In these cases, however, because the affected proband in the twin-ship is known to have MS, he or she (and, thus, also their twin) will already be in either the (P2) or (P3) age-band. Therefore, for all ascertained pairs (with at least 1 affected) the degree of underestimation for $P(MS \mid DZ_{MS})$ and $P(MS \mid MZ_{MS})$ will be less than it is for P(MS). Nevertheless, from Prop. (4.2); Section C, the estimate of P(G) is derived from the ratio of these two quantities such that:

$$P(G) \leq 2*(1.86)*\{P(MS) / P(MS \mid MZ_{MS})\}$$

Therefore, the under-estimate of P(G) from using P(MS | P) – i.e., by using Assumption (A1) – will be mitigated, to some extent, in the ratio.

4. The Number and Uniqueness of Susceptible Genotypes

It seems that individual MS patients are unlikely to share specific susceptibility genotypes with other MS patients. Thus, both from recent genome-wide screens[26] and from theoretical considerations alone,[27] it seems that there are approximately 100-200 susceptibility loci in the human genome. In addition, it seems that, on average, between 11 and 18 of these loci need to be in a susceptible state in order to confer susceptibility.[27] Under these circumstances, the number of different susceptible combinations (N) will be huge. For example, assuming that there are 100 loci, 11 of which are necessary, yields:

$$N = (100!) / \{(89!)(11!)\} = 1.4 \times 10^{14} \text{ genetic combinations}$$

With 15 necessary loci, this calculation yields:

$$N = (100!) / \{(85!)(15!)\} = 2.5 \times 10^{17} \text{ genetic combinations}$$

Thus, regardless of the exact distribution of the number of susceptible loci necessary for each susceptible genotype, with only 7 billion people on earth (of whom, less than 5% are susceptible), it is unlikely that any more than a tiny fraction of MS patients actually share the exact same combination of susceptibility genes with another MS patient. Nevertheless, even granting this conclusion, this does not exclude the possibility that patients might still be classifiable into "clusters" of genetic associations. In this view, it may be possible to subdivide the universe of susceptible genotypes (i.e., combinations of "susceptible genes") into a more manageable number of different, but possibly overlapping, groups. Thus, perhaps, each group would share certain properties (e.g., expected penetrance, involvement of specific pathways, and so forth) although no member of the group would share an identical collection of susceptibility genes with any other member. Nor would they, necessarily, share any specific subset of susceptibility genes. Rather, for example, each member of the group might possess some number of a cluster of genes in addition to whatever else is necessary to make their particular genotype susceptible.

Consequently, in order to identify these "clusters" (if they exist) using a GWAS approach in large datasets,[26] it is important to test as many different combinations of as many different associated genes as possible to explore these "group-associations" with MS. In addition, because gender and HLA-status impact MS-susceptibility (see Main Text & Section D), it is important to use this "cluster" approach, not

only for the population as a whole, but also for the different subgroups broken down by gender (men or women) and/or by HLA-status (carriers of 0, 1, or 2 copies of the DRB1*1501 allele).

Moreover, as discussed in the text and in Section D, the prevalence of women in the susceptible subset (G) is low (28 – 48%). There are (at least) two possible explanations for this circumstance. First, it is possible that the genes, which are associated with MS, are different between men and women. Second, susceptible women may, on average, require more susceptibility alleles to MS than susceptible men.[27] Therefore, it would be interesting (and important) to perform the GWAS analyses, separately by gender, to determine both whether the same set of genes are associated in men and women and, also, whether MS-women possess more of the ~100 identified susceptibility-genes [26] than MS-men.

Finally, as noted earlier,[27] part of the DRB1*1501 effect is seems to be due to reduction in the number of susceptibility genes needed to produce susceptibility. If this reduction is of greater magnitude in women than men, it might help to explain the gender-difference in MAF between MS-men and MS-women (see Main Text & Prop. 6.4; Section D). Therefore, in the large datasets now becoming available,[26] it would be important to confirm that the MAF difference between men and women actually exists, to confirm the observation that each DRB1*1501 allele and each "(HLA–) allele" has an independent impact on susceptibility, and to compare the number of susceptibility genes present for the different subgroups broken down both by gender and by HLA-status.

Appendix S1 ; Section C

Establishing Relationships and Estimating the Parameters

Propositions

Proposition 1 .... p. 1

Proposition 2 .... p. 7

Proposition 3 .... p. 11

Proposition 4 .... p. 14

Proposition 5 .... p. 16

Large Red Rectangles above represent hyperlinks to main parts of Section C

Small Red Boxes within Document represent hyperlinks within Appendix S1.

Small Green Boxes within Appendix S1 are hyperlinks back to Main Text

Navigate Back to Main Menu

Proposition 1:

1. $P(MS) > P(MS \mid G-)$

2. $P(MS \mid G-, Gx+) = P(MS \mid G-, Gx-) = P(MS \mid G-, S_{MS}) = P(MS \mid G-)$

3. $P(MS, G \mid MZ_{MS}) > (0.90)\, P(MS \mid MZ_{MS}) = 0.224$

4. $P(MS \mid IG_{MS}) = \mathbf{b} = 0.134$

5. $P(MS, G \mid IG_{MS}) > (0.99)*P(MS \mid IG_{MS}) \approx P(MS \mid IG_{MS}) = \mathbf{b}$

6. $P(MS \mid G, MZ_{MS}) \geq P(MS \mid G, IG_{MS}) = \mathbf{b}/g = \mathbf{b'} \geq \mathbf{b}$

7. If the Gx+ state is associated with MS, then, so too, is the Gx– state

Definitions for Proposition 1: (See also Table 1; Main Paper)

1. (MZ_{MS}), (DZ_{MS}), (S_{MS}) = sets of persons who have a monozygotic (MZ)-twin, a dizygotic (DZ)-twin, or a sibling (S) with MS but whose MS-status is unknown.

   (IG_{MS}) = hypothetical set of persons with identical genotypes (IG) to the set (MZ_{MS}) but who have the environmental experience (including both intra-uterine and childhood) of the general population.

2. (G), (G–) = sets of persons who are (G) or are not (G–) genetically susceptible to MS (see Section B).

   $P(MS \mid G) > P(MS \mid G-)$ # From the definition of $P(MS \mid G-)$; Section B

   $P(G) + P(G-) = 1$ # (G) & (G–) partition the population

3. (Gx+), (Gx–) = sets of persons who either have (Gx+) or lack (Gx–) the (Gx) genetic characteristic.

   $P(Gx+) + P(Gx-) = 1$ # (Gx+) & (Gx–) partition the population

4. (IU), (CH) = sets of persons who share intra-uterine (IU) or childhood (CH) environments with an MS proband.

5. $\mathbf{b} = P(MS \mid IG_{MS})$; $\mathbf{b'} = P(MS \mid G, IG_{MS})$; $\mathbf{t} = P(MS \mid Gx+, IG_{MS})$

   $\mathbf{s} = P(MS \mid Gx-, IG_{MS})$; $g = P(G \mid MS) = P(G \mid IG_{MS}) = P(G \mid MZ_{MS})$

   $g_1 = P(G \mid Gx+, IG_{MS})$; $g_2 = P(G \mid Gx-, IG_{MS})$ $m = P(MS \mid DZ_{MS}) / P(MS \mid S_{MS})$

   $m_1 = P(MS \mid Gx+, DZ_{MS}) / P(MS \mid Gx+, S_{MS})$ $m_2 = P(MS \mid Gx-, DZ_{MS}) / P(MS \mid Gx-, S_{MS})$

Observed Relationships:

1. $P(MS, G) > 0$; and, thus: $P(G) > 0$ # At least some cases of MS are due to genetic factors [3, 6, 9, 13, 17, 20]

2. $P(MS \mid CH) = P(MS)$ # MS is independent of the shared CH micro-environment [4-7, 9, 10, 19, 20]

3. $P(G \mid DZ_{MS}) = P(G \mid S_{MS})$ # DZ twins and siblings are genetically similar

4. $P(MS \mid MZ_{MS}) = P(MS \mid IU, CH, IG_{MS}) = P(MS \mid IU, IG_{MS})$ # From Definitions (1 & 4); Relationship (2)

5. $P(MS \mid DZ_{MS}) = P(MS \mid IU, CH, S_{MS}) > P(MS \mid S_{MS})$ # From Definitions (1 & 4); Relationship (2)

Assmptuions:

A1. P(MS) is approximately equal to the prevalence of MS in the population (see Section B for a discussion)

A2. $P(MS \mid G–, S_{MS}) = P(MS \mid G–, CH) = P(MS \mid G–, IG_{MS}) = P(MS \mid G–)$ # See Prop. (1.2a) for a discussion

A3. For Prop. (1.3), we assume that the observed increased risk of MS from the IU environment (see observed relationship #5 above) applies to both the (G) and the (G–) subsets (although not necessarily equally to each).

Thus, we assume that: $P(MS, G \mid DZ_{MS}) \geq P(MS, G \mid S_{MS})$ and: $P(MS, G– \mid DZ_{MS}) \geq P(MS, G– \mid S_{MS})$

A4. For Props. (1.4b, 1.5b, 5 & 6), we assume that: $m_1 = m_2$

Proof of Proposition 1.1

$P(MS) = P(MS, G) + P(MS, G–) = P(G)*P(MS \mid G) + P(G–)*P(MS \mid G–)$

Because, by definition: $P(MS \mid G) > P(MS \mid G–)$; and: $P(G) + P(G–) = 1$

Therefore: $P(MS) > P(G)*P(MS \mid G–) + P(G–)*P(MS \mid G–) = P(MS \mid G–)$

Also: $P(MS, G) = (g)*P(MS) \leq P(MS)$ # $0 < g \leq 1$

Proof for Proposition 1.2:

1.2a. By definition, $P(MS \mid G–)$ has the least penetrance of any genotype. Moreover, based on observations from Canada, the impact of a shared CH environment on disease occurrence seems to be minimal.[4-7, 9, 10, 19, 20]

Thus, we assume that: $P(MS \mid G–, S_{MS}) = P(MS \mid G–, CH) = P(MS \mid G–)$ # Assumption (A2)

The term (IG_{MS}) is defined, specifically, to exclude the impact of the CH and IU environments beyond any possible impact of CH in siblings (see Prop. 1.4a). Therefore, also: $P(MS \mid G–, IG_{MS}) = P(MS \mid G–)$

The set (G–) has the lowest penetrance of any genotype (see Section B).

Therefore: $P(MS \mid G–, Gx+) = P(MS \mid G–, Gx–) = P(MS \mid G–)$

1.2b. From Props. (1.1) & (1.2a):

$P(MS, G– \mid S_{MS}) = P(G– \mid S_{MS})*P(MS \mid G–, S_{MS}) = P(G– \mid S_{MS})*P(MS \mid G–) < P(MS)$

Thus: $P(MS, G \mid S_{MS}) = P(MS \mid S_{MS}) – P(MS, G– \mid S_{MS}) > P(MS \mid S_{MS}) – P(MS)$

or: $P(MS, G \mid S_{MS}) > P(MS \mid S_{MS}) – P(MS) = 0.029 – 0.0015 = 0.0275$ # Data: Table (2)

so that: $P(MS, G \mid S_{MS}) > (0.0275 / 0.029)*P(MS \mid S_{MS}) = (0.95)*P(MS \mid S_{MS})$

Consequently, over 95% of concordant MS in siblings is due to genetic susceptibility. This percentage increases to much more than (95%) when a more realistic estimate for $P(MS, G– \mid S_{MS})$ is used.

Proof for Proposition 1.3:

Because: $P(MS \mid DZ_{MS}) = 0.054 > 0.029 = P(MS \mid S_{MS})$ # Data: Table (2)

and: $P(G \mid DZ_{MS}) = P(G \mid S_{MS})$ # DZ-twins are genetically siblings

Therefore, the shared intrauterine (IU) environment, the more similar childhood (CH) environment of DZ-twins (compared to non-twin siblings), or both, increase the risk of MS. However, the fact that all siblings share similar CH environments, together with the actual evidence,[4-7, 9, 10, 19, 20] suggest that this is increased MS-risk in twins is due, almost entirely, to an IU environmental effect.

Therefore: $P(MS \mid DZ_{MS}) = P(MS \mid IU, S_{MS}) = P(MS, G \mid IU, S_{MS}) + P(MS, G– \mid IU, S_{MS})$

And: $P(MS \mid DZ_{MS}) = P(MS, G \mid IU, S_{MS}) + P(MS, G– \mid IU)$ # Prop. (1.2a)

Also: $P(MS, G \mid IU, S_{MS}) \geq P(MS, G \mid S_{MS}) > 0.0275$ # Prop. (1.2b) & Assumption (A3)

(1) Thus: $P(MS, G– \mid IU) = P(MS, G– \mid DZ_{MS}) = P(MS \mid DZ_{MS}) – P(MS, G \mid IU, S_{MS}) < 0.054 – 0.0275 = 0.0265$

Using the same IU adjustment in MZ-twins (and Assumption A3) then:

$P(MS, G \mid MZ_{MS}) = P(MS \mid MZ_{MS}) – P(MS, G– \mid IU) > 0.25 – 0.0265 = 0.224$ # Eq. (**1**)

Thus: $P(MS, G \mid MZ_{MS}) > (0.224 / 0.25)*P(MS \mid MZ_{MS}) = (0.90)*P(MS \mid MZ_{MS})$

Proof of Proposition 1.4:

1.4a. $P(MS \mid DZ_{MS}) = P(MS \mid IU, S_{MS}) = m*P(MS \mid S_{MS})$; $m = 0.054 / 0.029 = 1.86$ # Data: Table (2)

Using the same IU adjustment for MZ-twins, then: $P(MS \mid MZ_{MS}) = (m)*P(MS \mid IG_{MS})$

And, thus: $\mathbf{b} = P(MS \mid IG_{MS}) = P(MS \mid MZ_{MS}) / (m) = (0.25) / (1.86) = 0.134$ # Data: Table (2)

1.4b. The quantities (m_1) and (m_2) are defined such that:

$\mathbf{t} = P(MS \mid Gx+, IG_{MS}) = P(MS \mid Gx+, MZ_{MS}) / m_1$

and also: $\mathbf{s} = P(MS \mid Gx–, IG_{MS}) = P(MS \mid Gx–, MZ_{MS}) / m_2$

which is subject to the condition that: $m\mathbf{b} = P(Gx+ \mid MS)*(m_1)\mathbf{t} + P(Gx– \mid MS)*(m_2)\mathbf{s}$

From Assumption (A4): $m_1 = m_2$; and, therefore: $m_1 = m_2 = m$

For the gender partition (Gx+ = F), the actual data from Table 2 suggests, if anything, that:

$m_1 = (0.051 / 0.039) = 1.31 < 3.0 = (0.057 / 0.019) = m_2$

Such a violation of Assumption (A4) would only serve to increase the estimated excess of men in the genetically susceptible population (G) – a condition, which, as indicated in Prop (6.2a), already exists

Proof of Proposition 1.5:

1.5a. Using the logic of Prop. (1.2b) – [i.e., substituting IG_{MS} for S_{MS}] – & from Prop. (1.1), therefore:

$$P(MS, G– \mid IG_{MS}) = P(G– \mid IG_{MS})*P(MS \mid G–, IG_{MS}) = P(G– \mid IG_{MS})*P(MS \mid G–) < P(MS)$$

Thus: $P(MS, G \mid IG_{MS}) = P(MS \mid IG_{MS}) – P(MS, G– \mid IG_{MS}) > P(MS \mid IG_{MS}) – P(MS) = \mathbf{b} – P(MS)$

where: $\mathbf{b} – P(MS) = 0.134 – 0.0015 = 0.1325$; and consequently:

(2) $$P(G \mid MS, IG_{MS}) = P(MS, G \mid IG_{MS}) / P(MS \mid IG_{MS}) > [\mathbf{b} – P(MS)] / \mathbf{b} = 0.1325 / 0.134 = 0.99$$

With a more realistic estimate of $P(MS, G– \mid IG_{MS})$, this estimate of (> 99%) is increased still further.

1.5b. From Eq. (**2**), therefore, for all practical purposes: $\mathbf{b} = P(MS \mid IG_{MS}) = P(MS, G \mid IG_{MS})$

Thus, the sets (MS, IG_{MS}) and (MS, G, IG_{MS}) are the same. Therefore, also:

$$\mathbf{t} = P(MS \mid Gx+, IG_{MS}) = P(MS, G \mid Gx+, IG_{MS})$$

$$\mathbf{s} = P(MS \mid Gx–, IG_{MS}) = P(MS, G \mid Gx–, IG_{MS})$$

Proof of Proposition 1.6:

From Prop. (1.5b) and Assumption (A2):

$$P(MS \mid IG_{MS}) = P(MS, G \mid IG_{MS}) = P(G \mid IG_{MS})*P(MS \mid G, IG_{MS}) = g*P(MS \mid G, IG_{MS})$$

Therefore: $P(MS \mid G, IG_{MS}) = P(MS \mid IG_{MS}) /g = \mathbf{b}/g = \mathbf{b'} \geq \mathbf{b}$

Similarly: $P(MS \mid Gx+, G, IG_{MS}) = \mathbf{t}/g_1$; and: $P(MS \mid Gx–, G, IG_{MS}) = \mathbf{s}/g_2$

Proof of Proposition 1.7:

1.7a. If: $P(G \mid Gx+) = P(G)$; then: $P(G, Gx+) = P(G)*P(Gx+)$ # i.e., if: (G) and (Gx+) are independent

However: $P(G) = P(G, Gx+) + P(G, Gx–) = P(G)*P(Gx+) + P(G)*P(Gx– \mid G)$

which yields: $1 – P(Gx+) = P(Gx–) = P(Gx– \mid G)$ # Dividing by P(G) & rearranging

Also: $P(Gx+) = P(G, Gx+) + P(G–, Gx+) = P(Gx+)*P(G) + P(Gx+)*P(G– \mid Gx+)$

which, yields: $1 – P(G) = P(G–) = P(G– \mid Gx+)$ # Dividing by P(Gx+) & rearranging

Similarly: $P(G–) = P(G–, Gx+) + P(G–, Gx–) = P(Gx+)*P(G–) + P(G–)*P(Gx– \mid G–)$

which yields: $1 – P(Gx+) = P(Gx–) = P(Gx– \mid G–)$ # Dividing by P(G–) & rearranging

Thus, the independence of (G) and (Gx+), implies the independence of (G) and (Gx–), of (G–) and (Gx+), and of (G–) and (Gx–).

1.7b. If: $P(MS \mid G, Gx+) = P(MS \mid G)$; then: $P(MS, Gx+ \mid G) = P(MS \mid G)*P(Gx+ \mid G)$

also: $P(MS \mid G) - P(MS, Gx- \mid G) = P(MS \mid G)*\{1 - P(Gx- \mid G)\} = P(MS \mid G) - P(MS \mid G)*P(Gx- \mid G)\}$

so that: $P(MS, Gx- \mid G) = P(MS \mid G)*P(Gx- \mid G)$; and, thus: $P(MS \mid G, Gx-) = P(MS \mid G)$

Alternatively, if we start with the condition: $P(MS \mid G, Gx+) = P(MS \mid G, Gx-)$

Then: $P(MS \mid G) = P(MS \mid G, Gx+)*P(Gx+ \mid G) + P(MS \mid G, Gx-)*P(Gx- \mid G)$

$= P(MS \mid G, Gx+)*\{P(Gx+ \mid G) + P(Gx- \mid G)\} = P(MS \mid G, Gx+)$

1.7c. Each argument in Props. (1.7a) & (1.7b) is reversible. Thus, each conclusion implies each starting condition.

Consequently: $P(G \mid Gx+) = P(G)$; if and only if: $P(G \mid Gx-) = P(G)$

and also: $P(MS \mid G, Gx+) = P(MS \mid G)$; if and only if: $P(MS \mid G, Gx-) = P(MS \mid G)$

1.7d. If the conditions of both Props. (1.7a) & (1.7b) hold, then:

(3) $P(MS, G \mid Gx+) = P(G \mid Gx+)*P(MS \mid G, Gx+) = P(G)*P(MS \mid G) = P(MS, G)$

and it also follows from Prop. (1.7c) that:

(4) $P(MS, G \mid Gx-) = P(G \mid Gx-)*P(MS \mid G, Gx-) = P(G)*P(MS \mid G) = P(MS, G)$

1.7e. If the conditions of Props. (1.7a) & (1.7b) hold, then, using Props. (1.2a) & (1.7a), it also follows that:

$P(MS, G-, Gx+) = P(G-, Gx+)*P(MS \mid G-, Gx+) = P(Gx+)*P(G-)*P(MS \mid G-) = P(Gx+)*P(MS, G-)$

(5) Dividing by P(Gx+) this becomes: $P(MS, G- \mid Gx+) = P(MS, G-)$

(6) and similarly: $P(MS, G- \mid Gx-) = P(MS, G-)$ # Prop. (1.7c)

From Eqs. (**3** & **5**), therefore:

$P(MS \mid Gx+) = P(MS, G \mid Gx+) + P(MS, G- \mid Gx+) = P(MS, G) + P(MS, G-) = P(MS)$

and similarly: $P(MS \mid Gx-) = P(MS)$ # Eqs. (**4**) & (**6**)

Therefore, in circumstances where Props. (1.7a) & (1.7b) hold, then Eq. (**3**) also holds.

{NB: Eq. (**3**) could hold under circumstances where Props. (1.7a) & (1.7b) do not hold}

Nevertheless, when Props. (1.7a) & (1.7b) do hold, (Gx+) status is independent of MS-status and, thus, whatever defines (Gx+) is not associated with MS.

Conversely, when Eq. (**3**) doesn't hold (i.e., where the Gx+ state is associated with MS), then it must also be the case that, at least, one of these conditions – Prop. (1.7a) or (1.7b) – does not hold; and also that Eq. (**4**) does not hold.

Consequently, if the (Gx+) state is associated with MS, then, so too, is the (Gx–) state.

Proposition 2: **1.** $P(MS \mid G, IG_{MS}) \geq P(MS \mid G)$ or: $\mathbf{b'} \geq \mathbf{z}$

$P(MS \mid G1, IG_{MS}) \geq P(MS \mid G1)$ or: $\mathbf{x'} \geq \mathbf{x}$

$P(MS \mid G2, IG_{MS}) \geq P(MS \mid G2)$ or: $\mathbf{y'} \geq \mathbf{y}$

2. $P(G1 \mid G, IG_{MS}) \geq P(G1 \mid G)$; and: $P(G2 \mid G, IG_{MS}) \leq P(G2 \mid G)$

3. $\mathbf{t'/s'} \geq \mathbf{z_t} / \mathbf{z_s} = E(\mathbf{z_j}) / E(\mathbf{z_k})$

<u>New Definitions for Proposition 2:</u> (See also Table 1; Main Paper)

1. $P(MS \mid FT), P(MS \mid ST)$ = probability that the first (FT) or second (ST) twin of an MZ twin-pair will get MS, independent of whatever has happened or will happen to their co-twin

2. $\mathbf{z} = P(MS \mid G)$ $\qquad P(i) = P(G_i \mid G)$

3. $P(MS \mid G_i) = \mathbf{z_i}$ $\qquad P(MS \mid G_j) = \mathbf{z_j}$ $\qquad P(MS \mid G_k) = \mathbf{z_k}$

4. G_i = the ith susceptibility genotype within (G) ; where: $E(\mathbf{z_i}) = \mathbf{z}$; and: $Var(\mathbf{z_i}) = \sigma_{zi}^2$

   G_j = the jth susceptibility genotype within (G, Gx+) ; where: $E(\mathbf{z_j}) = \mathbf{z_t}$; and: $Var(\mathbf{z_j}) = \sigma_{zj}^2$

   G_k = the kth susceptibility genotype within (G, Gx–) ; where: $E(\mathbf{z_k}) = \mathbf{z_s}$; and: $Var(\mathbf{z_k}) = \sigma_{zk}^2$

5. n_b = the total number of susceptible genotypes in (G)

   n_t = the total number of number susceptible genotypes in (G, Gx+)

   n_s = the total number of susceptible genotypes in (G, Gx–)

6. (G1) = High-penetrance subset of (G), such that: $(G_i \in G1) \mid \{\mathbf{z_i} > \mathbf{z}\}$

   (G2) = Low-penetrance subset of (G), such that: $(G_i \in G2) \mid \{\mathbf{z_i} < \mathbf{z}\}$

   If: $\mathbf{z_i} = \mathbf{z}$; then these genotypes are assigned to (G1) and (G2) evenly and randomly so that the sets (G1) and (G2) are mutually exclusive and form a partition of (G).

   Thus: $P(G1 \mid G) + P(G2 \mid G) = 1$

7. $\mathbf{x} = P(MS, G \mid G1) = P(MS \mid G, G1) = P(MS \mid G1)$ # Because: $(G1) \subset (G)$

   $\mathbf{y} = P(MS, G \mid G2) = P(MS \mid G, G2) = P(MS \mid G2)$ # Because: $(G2) \subset (G)$

   $\mathbf{x'} = P(MS, G \mid G1, IG_{MS}) = P(MS \mid G1, IG_{MS})$ # Because: $(G1) \subset (G)$

   $\mathbf{y'} = P(MS, G \mid G2, IG_{MS}) = P(MS \mid G2, IG_{MS})$ # Because: $(G2) \subset (G)$

8. $\mathbf{t'} = P(MS \mid G, Gx+, IG_{MS}) = P(MS \mid Gx+, IG_{MS}) / P(G \mid Gx+, IG_{MS})$ # Prop. (1.5b)

   $\mathbf{s'} = P(MS \mid G, Gx–, IG_{MS}) = P(MS \mid Gx–, IG_{MS}) / P(G \mid Gx–, IG_{MS})$ # Prop. (1.5b)

Assumptions:

A5. The genetic composition of the sets (MS), (IG_{MS}), and (MZ_{MS}) are the same.

Also: $P(MS \mid FT) = P(MS \mid ST) = P(MZ_{MS}) = P(IG_{MS}) = P(MS)$

i.e., the probability of being a first or second MZ-twin is independent of MS-status

Therefore, also, for the ith susceptibility genotype (G_i) within (G): $P(G_i \mid IG_{MS}) = P(G_i \mid MZ_{MS}) = P(G_i \mid MS)$

Consequently: $P(G \mid IG_{MS}) = P(G \mid MZ_{MS}) = P(G \mid MS) = g$

Finally, also: $P(Gx+ \mid IG_{MS}) = P(Gx+ \mid MZ_{MS})$; and: $P(Gx+ \mid G, IG_{MS}) = P(Gx+ \mid G, MZ_{MS})$

A6. The genetic composition of the sets (G, FT), (G, ST), and (G) are the same

Also: $P(G \mid FT) = P(G \mid ST) = P(G)$

i.e., the probability of being a first or second MZ-twin is independent of (G) status

A7. Given our convention that: $P(MS \mid G, Gx+) = \mathbf{z_t} \geq \mathbf{z_s} = P(MS \mid G, Gx–)$

For Props. (5 & 6), we assume that: $P(MS \mid G, Gx+, IG_{MS}) = \mathbf{t'} \geq \mathbf{s'} = P(MS \mid G, Gx–, IG_{MS})$

(see Prop. 2.3 for a consideration of the validity of this assumption)

Proof of Proposition 2.1:

$P(MS \mid G, G_i) = P(MS \mid G_i) = \mathbf{z_i}$; $G_i \in (G)$; For: $i = 1$ to n_b # By definition

Therefore, because (G_i) is a subset of (G) – i.e., $G_i \subset (G)$ – therefore:

$P(G, G_i) = P(G_i)$; $P(MS \mid G, G_i) = P(MS \mid G_i)$; and: $P(MS, G, G_i) = P(MS, G_i)$

By Assumptions (A5 & A6), for the sets (G_i, MS) and (G, MS); it must be the case that:

$(G_i, MS) = (G_i, IG_{MS})$; and: $(G, MS) = (G, IG_{MS})$

(1) Therefore: $P(G_i \mid G, MS) = P(G_i \mid G, IG_{MS})$; and: $P(G \mid MS) = P(G \mid IG_{MS})$

Among the population of susceptible individuals, the probability of the (i^{th}) genotype, P(i), is:

$P(i) = P(G_i \mid G)$; so that: $\sum_i P(i) = 1$

By definition, the penetrance of any specific genotype is expected to be the same under equivalent environmental circumstances. The quantity $P(MS \mid IG_{MS})$ has been specifically adjusted (Prop. 1.4a) to remove the impact of the similar environment that twins experience. Therefore, by definition:

(2) $P(MS \mid G, G_i, IG_{MS}) = P(MS \mid G, G_i) = P(MS \mid G_i, IG_{MS}) = P(MS \mid G_i) = \mathbf{z_i}$ # $G_i \subset (G)$

With these definitions and assumptions, by the definition of mathematical expectation for the discrete random variable ($\mathbf{z_i}$), and from the definition of the variance (σ_{zi}^2) of such a variable, therefore:

$$E(\mathbf{z_i}) = \sum_i (\mathbf{z_i})*P(i) = \sum_i P(MS \mid G, G_i)*P(G_i \mid G) = \sum_i P(MS, G_i \mid G) = P(MS \mid G) = \mathbf{z}$$

(3) $$E(\mathbf{z_i}^2) = \sum_i (\mathbf{z_i}^2)*P(i) = \sum_i (\mathbf{z_i}^2)*P(G_i \mid G) = \{E(\mathbf{z_i})\}^2 + \sigma_{zi}^2 = \mathbf{z}^2 + \sigma_{zi}^2$$

And, using Assumptions (A5 & A6) together with Eqs. (**1** & **2**) yields:

$$P(MS, G_i \mid G, IG_{MS}) = P(G_i \mid G, IG_{MS})*P(MS \mid G, G_i, IG_{MS}) = P(G_i \mid G, MS)*(\mathbf{z_i})$$

(4) where: $P(G_i \mid G, MS) = P(MS, G, G_i) / P(MS, G) = \{P(MS \mid G_i)*P(G_i \mid G)\} / P(MS \mid G) = (\mathbf{z_i})*P(G_i \mid G)/ \mathbf{z}$

Therefore: $P(MS, G_i \mid G, IG_{MS}) = \{(\mathbf{z_i})*P(G_i \mid G) / \mathbf{z}\}*(\mathbf{z_i}) = (\mathbf{z_i}^2)*P(G_i \mid G) / \mathbf{z} = (\mathbf{z_i}^2)*P(i) / \mathbf{z}$

Also, because: $\sum_i P(MS, G_i \mid G, IG_{MS}) = P(MS, G \mid G, IG_{MS}) = P(MS \mid G, IG_{MS}) = \mathbf{b'}$

Therefore, from Eq. (**3**), it follows that:

(5) $$\mathbf{b'} = \sum_i P(MS, G_i \mid G, IG_{MS}) = \sum_i (\mathbf{z_i}^2)*P(i) / \mathbf{z} = E(\mathbf{z_i}^2) / \mathbf{z} = \mathbf{z} + (\sigma_{zi}^2) / \mathbf{z} \geq \mathbf{z} = P(MS \mid G)$$

(6) Similarly: $$\mathbf{t'} = \sum_j (\mathbf{z_j}^2)*P(j) / \mathbf{z_t} = E(\mathbf{z_j}^2) / \mathbf{z_t} = \mathbf{z_t} + (\sigma_{zj}^2) / \mathbf{z_t} \geq \mathbf{z_t} = P(MS \mid G, Gx+)$$

(7) and: $$\mathbf{s'} = \sum_k (\mathbf{z_k}^2)*P(k) / \mathbf{z_s} = E(\mathbf{z_j}^2) / \mathbf{z_s} = \mathbf{z_s} + (\sigma_{zk}^2) / \mathbf{z_s} \geq \mathbf{z_s} = P(MS \mid G, Gx–)$$

Thus, the penetrance for susceptible individuals from the MZ_{MS} population is increased compared to the penetrance for susceptible individuals in the general population (NB: similar logic applies equally to its subsets).

Therefore, also: $P(MS \mid G1, IG_{MS}) \geq P(MS \mid G1)$; or: $\mathbf{x'} \geq \mathbf{x}$

and: $P(MS \mid G2, IG_{MS}) \geq P(MS \mid G2)$; or: $\mathbf{y'} \geq \mathbf{y}$

<u>Proof of Proposition 2.2:</u>

$$P(G_\mathbf{i} \mid G, MS) = P(G_\mathbf{i} \mid G, IG_{MS}) = \{(\mathbf{z_i})*P(G_i \mid G)\} / P(MS \mid G)$$ # Eq. (**4**) & Assumptions (A5) & (A6)

By convention, we will designate any pair of genotypes $\{(G_1)$ and $(G_2)\}$ such that: $\mathbf{z_1} \geq \mathbf{z_2}$

In this case, Eq. (**4**) can be rearranged (for each of the pair) to yield:

$$P(G_1 \mid G, IG_{MS}) / P(G_1 \mid G) = (\mathbf{z_1}) / P(MS \mid G)$$

and also: $$P(G_2 \mid G, IG_{MS}) / P(G_2 \mid G) = (\mathbf{z_2}) / P(MS \mid G)$$

Thus: $$P(G_1 \mid G, IG_{MS}) / P(G_1 \mid G) \geq P(G_2 \mid G, IG_{MS}) / P(G_2 \mid G)$$ # by our convention

By extension, this must also apply, collectively, to the genotypes in the (G1) and (G2) subsets.

Thus: $P(G1 \mid G, IG_{MS}) / P(G1 \mid G) \geq P(G2 \mid G, IG_{MS}) / P(G2 \mid G)$

Moreover, defining: $\mathbf{b_i'} = P(MS \mid IG_{MS}, G_i)$; then, as in Eq. (**4**), it follows that:

$$P(G_i \mid G, MS, IG_{MS}) = \{P(MS \mid IG_{MS}, G_i)*P(G_i \mid G, IG_{MS})\} / P(MS \mid G, IG_{MS}) = (\mathbf{b_i'})*P(G_i \mid G, IG_{MS})/ \mathbf{b'}$$

Therefore, substituting: $\mathbf{b_1'} \geq \mathbf{b_2'}$; for: $\mathbf{z_1} \geq \mathbf{z_2}$; into the above equations, and using the same logic as above for both (G_1) and (G_2), leads to the conclusion that:

$$P(G_1 \mid MS, G, IG_{MS}) / P(G_1 \mid G, IG_{MS}) \geq P(G_2 \mid MS, G, IG_{MS}) / P(G_2 \mid G, IG_{MS})$$

Consequently, more penetrant genotypes are enriched to a greater extent than less penetrant genotypes in both the (MS) and the (MS, IG_{MS}) populations. Also, because (G1) and (G2) partition (G), therefore:

$$P(G1 \mid MS, G, IG_{MS}) \geq P(G1 \mid G, IG_{MS}) \geq P(G1 \mid G)$$

and, also: $P(G2 \mid MS, G, IG_{MS}) \leq P(G2 \mid G, IG_{MS}) \leq P(G2 \mid G)$

<u>Proof of Proposition 2.3:</u>

We can define the discrete random variable (a_j) as the set of coefficients that randomly pair each of the (j) genotypes in (G, Gx+) with a genotype in a subset (kj) of the genotypes in (G, Gx–).

The penetrance of the (kj) subset will be defined as: $P(MS \mid G_{kj}) = \mathbf{z_{kj}}$

We can then chose the subset (kj) such that: $E(\mathbf{z_{kj}}) = \mathbf{z_s}$; and: $Var(\mathbf{z_{kj}}) = Var(\mathbf{z_k})$

If (j > k), then some of the genotypes in (G, Gx–) will be used more than once to make up the (kj) subset.

The (a_j) coefficients will be chosen such that: $\mathbf{z_j} = (a_j)(\mathbf{z_{kj}})$; where, we define: $E(a_j) = a \geq 1$

Because the sets (G, Gx+) and (G, Gx–) are mutually exclusive, the random variables ($\mathbf{z_j}$) and ($\mathbf{z_k}$) are expected to be independent. In this case, (a_j) and ($\mathbf{z_{kj}}$) will also be independent, as will (a_j) and ($\mathbf{z_k}$).

Therefore: $E(\mathbf{z_j}) = E(a_j\mathbf{z_{kj}}) = E(a_j)*E(\mathbf{z_{kj}}) = a\mathbf{z_s} = \mathbf{z_t}$

and: $Var(\mathbf{z_j}) = Var(a_j\mathbf{z_{kj}}) = (a)^2*Var(\mathbf{z_k}) + (\mathbf{z_t})^2*Var(a_j) + Var(\mathbf{z_k})*Var(a_j) \geq (a)^2*Var(\mathbf{z_k}) = Var(a\mathbf{z_k})$

In which case: $\mathbf{t'} = \mathbf{z_t} + Var(\mathbf{z_j}) / \mathbf{z_t} \geq (a)\mathbf{z_s} + (a)^2*Var(\mathbf{z_k}) / a\mathbf{z_s} = a\mathbf{s'}$ # Eqs. (**5 & 6**); Prop. (2.1)

(8) and, therefore: $\mathbf{t'/s'} \geq \mathbf{z_t} / \mathbf{z_s} = E(\mathbf{z_j}) / E(\mathbf{z_k}) = a \geq 1$

Thus, if ($\mathbf{z_j}$) and ($\mathbf{z_k}$) are independent, as expected, Assumption (A7) and Eq. (**8**) will necessarily hold.

Proposition 3:

1. a. $\mathbf{z} = p\mathbf{x} + (1 - p)\mathbf{y} \geq \mathbf{y}$; or: $p = (\mathbf{z} - \mathbf{y}) / (\mathbf{x} - \mathbf{y})$

   b. $q = p\mathbf{x} / [p\mathbf{x} + (1 - p)\mathbf{y}]$

2. a. $q'\mathbf{x} + (1 - q')\mathbf{y} = \mathbf{b'}$

   b. $p \leq q$

   c. If: $q = q'$; then: $\mathbf{b'} = \{(p\mathbf{x}^2) + (1 - p)\mathbf{y}^2\} / \mathbf{z}$

3. a. $q \leq q'$

   b. $\mathbf{x} \geq \mathbf{b'} \geq \mathbf{z} \geq \mathbf{y}$

4. a. $\mathbf{a} \geq \mathbf{x} \geq \mathbf{b'}$

   b. $\mathbf{a'} \geq \mathbf{b'}$

New Definitions for Proposition 3: See also previous Definitions in Props. (1) & (2); see also Table 1; Main Text

1. $p = P(G1 \mid G) = P(G, G1) / P(G) = P(G1) / P(G)$; $q = P(G1 \mid G, MS)$

   $q' = \{P(MS \mid G, IG_{MS}) - P(MS \mid G2)\} / \{P(MS \mid G1) - P(MS \mid G2)\} = (\mathbf{b'} - \mathbf{y}) / (\mathbf{x} - \mathbf{y})$

2. $\mathbf{a} = P(MS, G) / P(G1)$; $\mathbf{a'} = P(MS, G) / P(G2)$

Defined Relationships:

$P(IG_{MS}) = P(MZ_{MS}) = P(MS)$ # by definition of $P(MZ_{MS})$ and $P(IG_{MS})$; Assumption (A5 & A6)

$P(MS \mid G1) = \mathbf{x} \geq P(MS \mid G) \geq \mathbf{y} = P(MS \mid G2)$ # by the definitions of (G1) and (G2)

$P(G1) + P(G2) = P(G)$ # by definition, (G1) and (G2) partition (G)

$P(G, Gx+) + P(G, Gx–) = P(G)$ # by definition, (Gx+) and (Gx–) partition (G)

Proof for Proposition 3.1:

3.1a $P(MS, G \mid G1) = P(MS \mid G1)$; and: $P(MS, G \mid G2) = P(MS \mid G2)$ # (G1), (G2) ⊂ (G)

$P(MS, G) = P(G)*P(MS \mid G) = P(G1)*P(MS, G \mid G1) + P(G2)*P(MS, G \mid G2)$

$= p*P(G)\mathbf{x} + (1 - p)*P(G)\mathbf{y}$ # By definition of (p), ($\mathbf{x}$), and ($\mathbf{y}$)

Thus: $P(MS \mid G) = \mathbf{z} = p\mathbf{x} + (1 - p)\mathbf{y}$; or, with rearrangement: $p = (\mathbf{z} - \mathbf{y}) / (\mathbf{x} - \mathbf{y})$

Because: $\mathbf{x} \geq \mathbf{y}$; and: $p \geq 0$; therefore: $\mathbf{z} \geq \mathbf{y}$ # By the definitions

3.1b. $q = P(G1 | MS, G) = P(MS, G1 | G) / P(MS | G) = P(G1 | G)*P(MS | G1, G) / P(MS | G)$

$= \{p*P(MS | G1)\} / P(MS | G)$ # $(G1) \subset (G)$ & By definition of (p)

(**1**) Therefore: $q = p\mathbf{x} / [p\mathbf{x} + (1 - p)\mathbf{y}] = p(\mathbf{x}/\mathbf{z})$; and: $(1 - q) = (1 - p)(\mathbf{y}/\mathbf{z})$ # Prop. (3.1a)

Also: $P(MS | G, IG_{MS}) = P(G1 | G, IG_{MS})*P(MS | G1, IG_{MS}) + [1 - P(G1 | G, IG_{MS})]*P(MS | G2, IG_{MS})$

or: $\mathbf{b'} = q\mathbf{x'} + (1 - q)\mathbf{y'}$ # By the definitions

<u>Proof of Proposition 3.2:</u>

3.2a. By definition: $q' = \{P(MS | G, IG_{MS}) - P(MS | G2)\} / \{P(MS | G1) - P(MS | G2)\} = (\mathbf{b'} - \mathbf{y}) / (\mathbf{x} - \mathbf{y})$

Simple rearrangement leads to: $q'\mathbf{x} + (1 - q')\mathbf{y} = \mathbf{b'}$

3.2b Rearrangement of Eq. (**1**) yields: $p / (1 - p) = [q / (1 - q)]*[\mathbf{y} / \mathbf{x}]$

Therefore, if: $q < p$; then: $P(MS | G2) = \mathbf{y} > \mathbf{x} = P(MS | G1)$

However, because, by definition: $\mathbf{x} \geq \mathbf{y}$; therefore: $q \geq p$

3.2c. If: $q = q'$; then: $\mathbf{b'} = q\mathbf{x} + (1 - q)\mathbf{y} = \{p\mathbf{x}^2 + (1 - p)\mathbf{y}^2\} / \mathbf{z}$ # Prop. (3.2a) & Eq. (**1**)

and, thus: $P(G1 | MS, G, IG_{MS}) = q\mathbf{x} / \mathbf{b'} = p\mathbf{x}^2 / \mathbf{z}\mathbf{b'} = p\mathbf{x}^2 / \{p\mathbf{x}^2 + (1 - p)\mathbf{y}^2\}$

<u>Proof of Proposition 3.3:</u>

3.3a. Because: $\mathbf{b'} = P(MS | G, IG_{MS}) = q'\mathbf{x} + (1 - q')\mathbf{y}$ # Prop. (3.2a)

and, because: $\mathbf{b'} = P(MS | G, IG_{MS}) = q\mathbf{x'} + (1 - q)\mathbf{y'}$ # Prop. (3.1b)

and, because: $\mathbf{x'} \geq \mathbf{x}$; and: $\mathbf{y'} \geq \mathbf{y}$ # Prop. (2.1)

Therefore: $\mathbf{b'} = q'\mathbf{x} + (1 - q')\mathbf{y} = q\mathbf{x'} + (1 - q)\mathbf{y'} \geq q\mathbf{x} + (1 - q)\mathbf{y}$

Simple rearrangement leads to: $(q' - q)(\mathbf{x} - \mathbf{y}) \geq 0$

However, because, by definition: $\mathbf{x} \geq \mathbf{y}$; therefore: $q' \geq q$

3.3b. It follows from the definitions and from Props. (3.1a), (3.2a), & (3.3a) that:

$(\mathbf{b'} - \mathbf{y}) / (\mathbf{x} - \mathbf{y}) = q' \geq q \geq p \geq 0$; and, therefore: $\mathbf{b'} \geq \mathbf{y}$ # Because $\mathbf{x} \geq \mathbf{y}$

Moreover, if: $\mathbf{x} < \mathbf{b'}$; then: $\mathbf{b'} = q'\mathbf{x} + (1 - q')\mathbf{y} < q'\mathbf{b'} + (1 - q')\mathbf{y}$

or, with rearrangement: $\mathbf{y} > \mathbf{b'} > \mathbf{x}$

However, because, by definition: $\mathbf{x} \geq \mathbf{y}$; therefore, we conclude that: $\mathbf{x} \geq \mathbf{b'} \geq \mathbf{y}$

Proof of Proposition 3.4:

3.4a We define: $\mathbf{a} = P(MS, G) / P(G1) \geq P(MS, G1) / P(G1) = P(MS \mid G1) = \mathbf{x}$ # $(G1) \subset (G)$

Therefore: $\mathbf{a} \geq \mathbf{x} \geq \mathbf{b'} \geq \mathbf{y}$ # Combined with Prop. (3.3b)

3.4b. $\mathbf{a} = P(MS, G) / P(G1) = P(MS, G) / p{*}P(G) = P(MS \mid G) / p$ # By definition of (**a**)

$\mathbf{a'} = P(MS, G) / P(G2) = P(MS, G) / (1 - p){*}P(G) = P(MS \mid G) / (1 - p)$ # By definition of (**a'**)

Because: $p \leq 1$; and: $(1 - p) \leq 1$

Therefore: $\mathbf{a} \geq P(MS \mid G) = \mathbf{z}$; and: $\mathbf{a'} \geq P(MS \mid G) = \mathbf{z}$

Also, whenever (p) and (1 – p) represent the same percentage, then: $\mathbf{a} = \mathbf{a'}$

Throughout the domain of: $0 < p < 1$; {or , equivalently: $0 < (1 - p) < 1$}

$$(1 - p)\mathbf{a'} = p\mathbf{a} = P(MS \mid G) = \mathbf{z}$$

In this way (**a**) and (**a'**) mirror each other such that:

If it is true that: $\mathbf{a} \geq \mathbf{b'}$; throughout the domain of (p) # Prop. (3.4a)

Then it also must be true that: $\mathbf{a'} \geq \mathbf{b'}$; throughout the domain of (1 – p)

Therefore: $\mathbf{a'} \geq \mathbf{b'}$

Proposition 4:

1. $P(G) \geq P(MS, G) / \mathbf{b'} = (g^2) * P(MS) / \mathbf{b}$

2.

a. $0.010 \leq (g^2) * P(MS) / \mathbf{b} \leq P(G) \leq 2 * P(MS) / \mathbf{b} = 0.022$

b. $0.067 = \mathbf{b}/2 \leq \mathbf{z} \leq \mathbf{b'} \leq 0.143$

c. $g * P(MS) / \mathbf{z}_{max} \leq P(G) \leq 2*(1.86)*\{P(MS) / P(MS \mid MZ_{MS})\}$

d. $0 \leq P(MS \mid G–) \leq 0.000092$; $P(MS \mid G) \geq 728 * P(MS \mid G–)$

<u>Proof of Proposition 4.1:</u>

$P(MS, G) = g*P(MS)$; $m = 1.86$; $\mathbf{b'} = \mathbf{b}/g$; and: $\mathbf{b'} \geq \mathbf{z}$ # Props. (1.1), (1.4a), (1.6) & (2.1)

(1) Thus: $P(G) = P(MS, G)/ \mathbf{z} \geq P(MS, G)/ \mathbf{b'} = (g^2)*\{P(MS) / \mathbf{b})\} = (g^2)(1.86)*\{P(MS) / P(MS \mid MZ_{MS})\}$

<u>Proof of Proposition 4.2:</u>

4.2a. $\mathbf{a} = P(MS, G) / P(G1) = P(MS, G) / p*P(G) \geq \mathbf{b'}$ # Prop. (3.4a)

so that: $P(G) \leq \{P(MS, G) / \mathbf{b'}\} / p$

Similarly: $P(G) \leq \{P(MS, G) / \mathbf{b'}\} / (1 – p)$ # From Prop. (3.4b)

(2) Therefore: $P(G) \leq \{P(MS, G) / \mathbf{b'}\} / p \leq \{P(MS) / \mathbf{b}\} / p$ # $P(MS) \geq P(MS, G)$; $\mathbf{b'} \geq \mathbf{b}$

(3) and: $P(G) \leq \{P(MS, G) / \mathbf{b'}\} / (1 – p) \leq \{P(MS) / \mathbf{b}\} / (1 – p)$ # $P(MS) \geq P(MS, G)$; $\mathbf{b'} \geq \mathbf{b}$

Because: $(0 \leq p \leq 1)$; one of the following three statements must be true:

(4) (#1) $p > 0.5$; (#2) $(1 – p) > 0.5$; or: (#3) $p = (1 – p) = 0.5$

Thus, from Eqs. (**2** & **3**), together with requirement of Eq. (**4**) & Prop. (4.1), it follows that:

(5) $P(MS, G) / \mathbf{b'} \leq P(G) = P(MS, G) / \mathbf{z} \leq 2*P(MS) / \mathbf{b} = 2*(1.86)*\{P(MS) / P(MS \mid MZ_{MS})\}$

Together with Eq. (**1**) this becomes:

(**6**) $(g^2)(1.86)*\{P(MS) / P(MS \mid G, MZ_{MS})\} \leq P(G) \leq (3.72)*\{P(MS) / P(MS \mid MZ_{MS})\}$

Because P(MS) and $P(MS \mid MZ_{MS})$ are directly observed parameters (Table 2), together with Props. (1.4a), (4.1), & (5.2b), then Eq. (**6**) yields the estimate:

$\{(0.94)^2(1.86)(0.0015)\}/ (0.25) = 0.010 \leq P(G) \leq \{(3.72)(0.0015)\}/ (0.25) = 0.022$ # $0.94 \leq g \leq 1$

Consequently, in MS, the maximum possible value for P(G) in the general population is 2.2%. A very similar range-estimate for P(G) is derived from epidemiological data obtained from different populations throughout North America and Europe (Table 7).

4.2b. In addition, rearrangement of Eq. (**5**), together with Eq. (**5**) of Prop. (2.1), yields:

(7) $\mathbf{b'} \geq P(MS \mid G) = \mathbf{z} \geq \mathbf{b'}/2 \geq \mathbf{b}/2 = 0.067$ # **b'** ≥ **b**

Again using the Prop. 5.2b estimate that: $0.94 \leq g \leq 1$

leads to: $0.134 \leq \mathbf{b'} = \mathbf{b}/g \leq (0.134 / 0.94) = 0.143$

(8) and, thus $0.067 \leq \mathbf{z} \leq \mathbf{b'} \leq 0.143$

In addition, Eq. (**5**) of Prop. (2.1) can be solved such that:

$$\mathbf{b'} = \mathbf{z} + (\sigma_{zi}^2) / \mathbf{z} \;; \quad \text{or:} \quad \sigma_{zi}^2 = \mathbf{z}*(\mathbf{b'} - \mathbf{z})$$

Therefore, using the Eq. (**8**) range-estimate for (**z**), yields the estimate that:

(9) $0 \leq \sigma_{zi}^2 \leq 0.0051$; or equivalently: $0 \leq \sigma_{zi} \leq 0.071$

4.2c. A narrower range-estimate for (**z**), and, therefore, also for (σ_{zi}^2) is possible. # Props. (7.1a) & (7.1b)

(10) Thus, we note that: $P(G) = P(MS, G) / \mathbf{z} \leq P(MS) / \mathbf{z}$ # By definition

(11) and that, under any circumstance: $\mathbf{b}/2 \leq \mathbf{z} \leq \mathbf{b'}$ # Eq. (**7**)

Defining ($\mathbf{z}_{min}$) and ($\mathbf{z}_{max}$) as the minimum and maximum levels of the range-estimate for (**z**) and using the range-estimate in Eq. (**11**), then: $\mathbf{z}_{min} = \mathbf{b}/2$; and: $\mathbf{z}_{max} = \mathbf{b'}$

and substitution of these into Eq. (**10**) yields: $g*P(MS) / \mathbf{z}_{max} \leq P(G) \leq P(MS) / \mathbf{z}_{min}$

(12) or: $(g^2)*P(MS) / \mathbf{b} \leq P(G) \leq 2*P(MS) / \mathbf{b}$ # Which is equivalent to Eqs. (**5 & 6**)

However, because the circumstance in which: $\mathbf{z} = \mathbf{b'}$; implies a zero variance, this estimate is almost certainly too high. Therefore, the most useful form for Eq. (**12**) to take is:

(13) $(g)*P(MS) / \mathbf{z}_{max} \leq P(G) \leq 2*(1.86)*\{P(MS) / P(MS \mid MZ_{MS})\}$

4.2d. From Prop. (5.2b): $P(MS, G–) \leq (0.06)*P(MS) = (0.06)(0.0015) = 0.00009$

Consequently: $P(MS \mid G–) = P(MS, G–) / P(G–) \leq (0.00009 / 0.978) = 0.000092$

Thus: $0 \leq P(MS \mid G–) \leq 0.000092$

And therefore: $P(MS \mid G) \geq (0.067 / 0.000092)*P(MS \mid G–) = 728*P(MS \mid G–)$

Proposition 5:

1. $g_1 / g_2 \leq$ **t/s**

$g = Ag_1 + (1 - A)g_2$

2. **a**. For gender and HLA: $g_1 \geq g_2$

b. $0.94 \leq g \leq 1$; so that : $0.134 = \mathbf{b} \leq \mathbf{b'} \leq 0.143$

c. For gender and HLA: $\mathbf{s}/\mathbf{b} \leq \mathbf{s'}/\mathbf{b'} \leq (1.04)(\mathbf{s}/\mathbf{b})$

3. $P(G \mid MS) = g >> 0.77$

New Definitions for Proposition 5:

The set G can be partitioned into two disjoint subsets (Gx+ and Gx–) based upon whether or not the susceptible person carries a specific genetic characteristic (Gx). Moreover, as in Prop. (2), the labeling convention adopted is that:

$$P(MS \mid G, Gx+) \geq P(MS \mid G, Gx-)$$

Because: $P(G, Gx+ \mid MS) + P(G, Gx- \mid MS) = P(G \mid MS) = g$; therefore all partitions estimate the same (g).

1. (F), (M) = sets of women (F) and men (M)

   (HLA+), (HLA–) = sets of DRB1*1501 carriers (HLA+) and non-carriers (HLA–)

2. $g_{01} = P(G \mid Gx+)$; $g_{02} = P(G \mid Gx-)$

3. $\mathbf{t'} = P(MS \mid G, Gx+, IG_{MS}) = P(MS \mid Gx+, IG_{MS}) / P(G \mid Gx+, IG_{MS}) = \mathbf{t}/g_1$ # As in Props. (1.5b) & (1.6)

   $\mathbf{s'} = P(MS \mid G, Gx-, IG_{MS}) = P(MS \mid Gx-, IG_{MS}) / P(G \mid Gx-, IG_{MS}) = \mathbf{s}/g_2$ # As in Props. (1.5b) & (1.6)

4. $A_0 = P(Gx+)$; $R_0 = P(Gx+ \mid G)$

   $A = P(Gx+ \mid IG_{MS}) = P(Gx+ \mid MS)$ $R = P(Gx+ \mid G, IG_{MS}) = P(Gx+ \mid G, MS)$

   $A_1 = P(Gx+ \mid MS, IG_{MS})$ $R_1 = P(Gx+ \mid MS, G, IG_{MS})$

Data for Proposition 5: # For the Gender and HLA partitions; From Tables (2 – 6)

$P(F) = A_0 = 0.5$	$P(HLA+) = A_0 = 0.24$
$P(F \mid IG_{MS}) = P(F \mid MS) = A = 0.68$	$P(HLA+ \mid IG_{MS}) = P(HLA+ \mid MS) = A = 0.55$
$P(F \mid MS, IG_{MS}) = A_1 = 0.92$	$P(HLA+ \mid MS, IG_{MS}) = A_1 = 0.57$

<u>Proof of Proposition 5.1:</u>

$\mathbf{t'} \geq \mathbf{b'} \geq \mathbf{s'}$; $\mathbf{t'} = \mathbf{t}/g_1$; and: $\mathbf{s'} = \mathbf{s}/g_2$ # Assumption (A7), Prop. (2.3) & as in Prop. (1.6)

(**1**) Thus: $g_2/g \geq \mathbf{s}/\mathbf{b}$; $\mathbf{s'}/\mathbf{b'} = (g/g_2)(\mathbf{s}/\mathbf{b})$; $g_1/g \leq \mathbf{t}/\mathbf{b}$; and: $g_1/g_2 \leq \mathbf{t}/\mathbf{s}$

$P(G \mid MS) = P(Gx+ \mid MS)*P(G \mid MS, Gx+) + P(Gx– \mid MS)*P(G \mid MS, Gx–)$

(**2**) or: $g = Ag_1 + (1 – A)g_2$ # Because: $1 – A = P(Gx– \mid MS)$

and: $\mathbf{b} = P(Gx+ \mid IG_{MS})\mathbf{t} + P(Gx– \mid IG_{MS})\mathbf{s}$ # Assumption (A4) ; Prop. (1.4b)

so that: $\mathbf{b'} = P(Gx+ \mid IG_{MS})*(g_1/g)\mathbf{t'} + P(Gx– \mid IG_{MS})*(g_2/g)\mathbf{s'}$ # Prop. (1.6)

Rearranging Eq. (**2**) yields: $A = (g – g_2) / (g_1 – g_2) \geq 0$; Therefore, one of three relationships must hold:

(**3**) (#1) $g_1 > g > g_2$; (#2) $g_1 < g < g_2$; or: (#3) $g_1 = g = g_2$

<u>Proof of Proposition 5.2:</u>

5.2a $P(MS, G–) = P(MS, Gx+, G–) + P(MS, Gx–, G–)$ # (Gx+, G–) & (Gx–, G–) partition (G–)

$P(Gx+, G–) = P(Gx+) – P(Gx+, G) \geq P(Gx+) – P(G)$ # (G) & (G–) partition the general population

and: $P(Gx–, G–) = P(Gx–) – P(Gx–, G) \geq P(Gx–) – P(G)$ # (G) & (G–) partition the general population

From Prop. (4.2a): $P(G) = P(G, Gx+) + P(G, Gx–) \leq 0.022$

Consequently: $A_0 = P(Gx+) \geq P(Gx+, G–) = A_0 – P(Gx+, G) \geq A_0 – 0.022$ # See Definition (4)

and: $(1 – A_0) = P(Gx–) \geq P(Gx–, G–) = (1 – A_0) – P(Gx–, G) \geq 0.978 – A_0$

(**4**) As a result: $1 \geq 1 – g_{01} = P(G– \mid Gx+) \geq (A_0 – 0.022) / A_0$ # See Definitions (2) & (4)

(**5**) and: $1 \geq 1 – g_{02} = P(G– \mid Gx–) \geq (0.978 – A_0) / (1 – A_0)$ # See Definitions (2) & (4)

(**6**) Also: $1 – g_1 = P(G– \mid MS, Gx+) = P(MS, G– \mid Gx+) / P(MS \mid Gx+)$ # See Definitions (2) & (4)

(**7**) where: $P(MS, G– \mid Gx+) = P(G– \mid Gx+)*P(MS \mid G–, Gx+) = P(G– \mid Gx+)*P(MS \mid G–)$ # Prop. (1.2a)

(**8**) and: $P(MS \mid Gx+) = P(MS, Gx+) / P(Gx+) = \{P(Gx+ \mid MS)*P(MS)\} / P(Gx+)$

Thus: $1 – g_1 = \{P(Gx+) / P(Gx+ \mid MS)\}*P(G– \mid Gx+)*\{P(MS \mid G–) / P(MS)\}$ # Eqs. (**6 – 8**)

or, equivalently: $1 – g_1 = (A_0 / A)*(1 – g_{01})*\{P(MS \mid G–) / P(MS)\}$

and similarly: $1 – g_2 = \{(1 – A_0) / (1 – A)\}*(1 – g_{02})*\{P(MS \mid G–) / P(MS)\}$

Rearranging these equations (when: g_{01}, g_{02}, g_1, and $g_2 < 1$), yields:

(9) $$(1 - g_1) \ / \ \{(A_0 / A)*(1 - g_{01})\} \ = \ (1 - g_2) / \{[(1 - A_0) / (1 - A)]*(1 - g_{02})\}$$

For convenience, we will define the term (B), such that:

(10) $$B \ = \ \{(A_0 / A)*(1 - g_{01})\} \ / \ \{[(1 - A_0) / (1 - A)]*(1 - g_{02})\} \ = \ (1 - g_1) / (1 - g_2)$$

so that Eq. (**10**) can be re-written as: $1 - g_1 = (1 - g_2)B$

(11) or, with rearrangement: $g_1 = B(g_2) + (1 - B)$

Thus: $g_1 \geq g \geq g_2$; if and only if: $B \leq 1$

Therefore, for any complex genetic disorder, we can estimate the permissible values of (g) using experimental data. Thus, the constraints of Eqs. (**4 & 5**), together with Eq. (**9**), combine to yield:

(12) $$\{(A_0 - 0.022)/A\}*\{(1 - A)/(1 - A_0)\} \ \leq \ B \ \leq \ (A_0/A)*\{(1 - A) / (0.978 - A_0)\}$$

As noted in the definitions, the same value of (g) will be estimated, regardless of which partition of (G) is chosen. Moreover, the parameter (g) can be estimated from the range of possible values that the parameter (B) can take and, in turn, this range can be estimated from directly-observed or directly-derived data. If the genetic characteristic (Gx) chosen to partition (G) <u>is not associated</u> with MS, then:

$P(G \mid Gx+) = P(G)$; and: $P(MS \mid G, Gx+) = P(MS \mid G)$

in which case: $P(MS, G \mid Gx+) = P(MS, G \mid Gx-) = P(MS, G)$ # Prop. (1.7d)

so that: $g_1 = g_2 = g$

and, thus: $B = 1$; for all possible values of (g) # Eq. (**10**)

Consequently, in this situation, Eq. (**11**) provides no information about the value of (g).

By contrast, if the genetic characteristic (Gx) that is chosen to partition (G) <u>is associated</u> with MS (Prop. 1.7), then the same estimate of (g) will be given by any such partition, in which case:

when: $B \neq 1$; then: $g_1 = g_2$; if and only if: $g = 1$ # Eqs. (**3**) & (**11**)

In the circumstances of MS, we have (available) observed data from two different partitions.

5.2a1. Thus, for the <u>gender partition</u> (Gx+ = F), Eqs. (**4** & **5**) yield:

$$1 \geq 1 - g_{01} = P(G-|F) \geq (0.5 - 0.022) / 0.5 = 0.96$$

$$1 \geq 1 - g_{02} = P(G-|M) \geq (0.5 - 0.022) / 0.5 = 0.96$$

Substituting these values, and the data from Tables 2 & 6, into Eq. (**12**) yields the range of:

$$0.450 \leq B \leq 0.492$$

In this case, from Table 6 and from Eqs. (**1** & **11**), therefore:

$$1 \leq g_1/g_2 = B + (1 - B) / g_2 \leq \mathbf{t/s} = (0.183 / 0.036) = 5.08$$

or: $g_2 \geq (1 - B) / \{\mathbf{t/s} - B)\}$

So that: $g_2 \geq (1 - 0.492) / (5.08 - 0.492) = 0.111$; and, thus, from Eq. (**11**): $1 \geq g_1 \geq 0.56$

(**13**) Eq. (**2**) then gives the estimate of: $0.42 \leq g \leq 1$

5.2a2. Similarly, for the <u>HLA partition</u> (Gx+ = HLA+), Eqs. (**4** & **5**) yield:

$$1 \geq 1 - g_{01} = P(G-|HLA+) \geq (0.24 - 0.022) / 0.24 = 0.91$$

$$1 \geq 1 - g_{02} = P(G-|HLA-) \geq (0.76 - 0.022) / 0.76 = 0.97$$

Substituting these values, and the data from Tables 2–6, into Eq. (**12**) yields the range of:

$$0.235 \leq B \leq 0.266$$

In this case, from Table 6 and from Eqs. (**1** & **11**), therefore:

$$1 \leq g_1/g_2 = B + (1 - B) / g_2 \leq \mathbf{t/s} = (0.166 / 0.154) = 1.08$$

or: $g_2 \geq (1 - B) / \{\mathbf{t/s} - B)\}$

So that: $g_2 \geq (1 - 0.266) / (1.08 - 0.266) = 0.90$; and, thus, from Eq. (**11**): $0.97 \leq g_1 \leq 1$

Eq. (**2**) then gives the estimate of: $0.94 \leq g \leq 1$

If: $P(HLA+|F, G) > P(HLA+|M, G)$ # See Table (2) & Prop. (6.4d)

Then, because: $P(MS|F, G) >> P(MS|M, G)$; # Prop. (6.2b)

our estimated (**t/s**) will be artificially high and, consequently, the estimate of ($g \geq 0.94$) will be too low.

5.2b. Because both partitions must estimate the same parameter (g), therefore, the only solution for (g) that is consistent with both estimates is: $0.94 \leq g \leq 1$;

(**14**) Consequently: $\mathbf{b} = 0.134 \leq \mathbf{b'} \leq (0.134) / (0.94) = 0.143$

5.2c. Combining Eqs. (**2** & **11**) yields:

(15) $g_2 \geq \{0.94 - (1 - B)A\} / (AB + 1 - A)$

For (Gx+ = F), Eqs. (**11** & **15**), together with the Prop. (5.2a1) estimate for B, yields:

$P(G \mid M, MS) = g_2 \geq 0.90$; and: $P(G \mid F, MS) = g_1 \geq 0.96$

Thus, for both (Gx+ = F) and (Gx+ = HLA+): $1 \leq g/g_2 \leq (0.94 / 0.90) = 1.04$

(16) and, therefore, from Eq. (**1**): **s/b** ≤ **s'/b'** ≤ 1.04(**s/b**) # For both of these partitions

<u>Proof of Proposition 5.3:</u>

5.3a. From Prop. (1.3) & Assumption (A3): $P(MS, G \mid MZ_{MS}) > (0.90)*P(MS \mid MZ_{MS})$

A population-wide survey of monozygotic twins in Finland identified 3,083 monozygotic twin-pairs born prior to 1957.[31] The authors reported that a total of 21 persons from this cohort had a diagnosis of MS and, of these, 10 pairs (3 concordant for MS) agreed to participate in the study. Using this information, together with Prop. (1.3), we can estimate the amount of genetic MS by the prevalence of concordant twins in this MZ-twin population. Thus:

$$P(MS, G, MZ_{MS}) > (0.90)*(3 / 10)*(21)*(1 / 3{,}083) = 0.00184$$

Because this estimate exceeds the reported prevalence of MS in Finland,[30] this observation also supports the notion that most MS cases develop through the genetic pathway.

5.3b. Even estimating the prevalence of MS in Finland from this particular cohort (excluding the second twins of concordant pairs) yields:

$$P(MS) = P(MZ_{MS}) = \{21 - (3 / 10)*(21)\} / \{(2)*(3{,}083) - (3 / 10)*(21)\} = 0.00239$$

Thus, the minimum estimated percentage of genetic MS in Finland (from this cohort) is:

$$P(MS, G, MZ_{MS}) / P(MZ_{MS}) > (0.00184 / 0.00239) = 0.77$$

However, because the prevalence of "genetic" MS should be far greater than the prevalence of just the concordant cases, therefore:

$$P(G \mid MS) = P(MS, G) / P(MS) >> P(MS, G, MZ_{MS}) / P(MZ_{MS}) = P(MS, G \mid MZ_{MS})$$

and, thus: $P(G \mid MS) = g = P(G \mid MZ_{MS}) >> 0.77$

Appendix S1; Section D

Impact of Gender & HLA DRB1*1501

Proposition 6.1 …………………………………………..… p. 1

Proposition 6.2 (Gender Status) …..………………......…… p. 3

Proposition 6.3 (HLA DRB1*1501 Status) ………………..… p. 6

Proposition 6.4 (Hardy-Weinberg Considerations) …...……….p. 10

Large Red Rectangles above represent hyperlinks to main parts of Section D

Small Red Boxes within Document represent hyperlinks within Appendix S1.

Small Green Boxes within Appendix S1 are hyperlinks back to Main Text

Navigate Back to Main Menu

Proposition 6: **1.** **a.** $R_1 \geq R \approx A$; # for all partitions

b. If only Mechanism (1) occurs: **s'/b'** = 1

c. If Mechanism (2) occurs at all: **s'/b'** < 1

2. $0.28 \leq P(F \mid G) \leq 0.49$

$(2.3)*P(MS \mid G, M) \leq P(MS \mid G, F) \leq (5.4)*P(MS \mid G, M)$

$0 \leq \sigma_{zj}^2 \leq 0.009$; and: $0 \leq \sigma_{zk}^2 \leq 0.0004$

3. $(3.72)*P(G \mid HLA–) \leq P(G \mid HLA+) \leq (3.87)*P(G \mid HLA–)$

$P(MS \mid G, 2HB+) \approx P(MS \mid G, 1HB+) \approx P(MS \mid G, HLA–) \approx P(MS \mid G)$

4. Each DRB1*1501 allele affects susceptibility independently

Definitions:

1. (HLA–), (1HB+), (2HB+), = sets of individuals who carry zero (HLA–), one (1HB+), or two (2HB+) copies of the DRB1*1501 allele ; Also: (1HB–) = set of individuals who carry one "non-DRB1*1501" allele

2. MAF = mean allelic frequency (defined broadly) ; HWE = Hardy Weinberg Equilibrium

3. W_p , W_q , W_{pq} = absolute fitness levels for the different genotypes.

w_p , w_q , w_{pq} = normalized fitness levels for the different genotypes.

$w = (w_p / w_q)^{½} > 1$ = "relative normalized fitness" at HWE. {appropriate definitions for other circumstances}

Mechanisms of Enrichment:

Only two mechanisms (see Prop. 1.7) can enrich Gx+ in an (MS) or an (MS, IG_{MS}) population. These are:

1) a MAF change such as: $P(Gx+ \mid G) > P(Gx+)$; or: $P(G \mid Gx+) > P(G \mid Gx–)$

and: 2) a Penetrance change such as: $P(MS \mid G, Gx+) > P(MS \mid G, Gx–)$

or: $P(MS \mid G, Gx+, IG_{MS}) > P(MS \mid G, Gx–, IG_{MS})$

Proof for Proposition 6.1:

6.1a. $R_1 = P(Gx+ \mid MS, G, IG_{MS}) = P(Gx+, G, IG_{MS})*P(MS \mid G, Gx+, IG_{MS}) / P(MS, G, IG_{MS})$

$= R*\{P(MS \mid G, Gx+, IG_{MS}) / P(MS \mid G, IG_{MS})\} = R*(\mathbf{t'}/\mathbf{b'})$

A comparable analysis leads to: $(1 – R_1) = (1 – R)*(\mathbf{s'}/\mathbf{b'})$

$R = R_0*\{P(MS \mid G, Gx+) / P(MS \mid G)\}$

and: $(1 – R) = (1 – R_0)*\{P(MS \mid G, Gx–) / P(MS \mid G)\}$

Three (Gx+) enrichment-stages occur for twin-populations: the 1st in going from the set (Gx+) to (Gx+, G); the 2nd in going from the set (Gx+, G) to (Gx+, G, MS) {or equivalently to (Gx+, G, IG_{MS})}; and the 3rd in going from the set (Gx+, G, IG_{MS}) to (Gx+, G, MS, IG_{MS}). Odds ratios (ORs) associated with these stages are:

$OR_1 = \{R_0 / (1 - R_0)\} / \{A_0 / (1 - A_0)\}$ # 1st stage

$OR_2 = \{R / (1 - R)\} / \{R_0 / (1 - R_0)\} = E(\mathbf{z_j}) / E(\mathbf{z_k}) = \mathbf{z_t / z_s}$ # 2nd stage

and: $OR_3 = \{R_1 / (1 - R_1)\} / \{R / (1 - R)\} = \mathbf{t'/ s'}$ # 3rd stage

The first of these enrichments (OR_1) is due to Mechanism (1) whereas the second and third (OR_2 and OR_3) are due to Mechanism (2). Because, from Prop. (5.2b): $g \approx 1$; therefore: $A \approx R$; and: $A_1 \approx R_1$

In this case, both (OR_3) and the combination of the first two enrichment stages ($OR_{1/2}$) can be directly observed.

(1) Thus: $OR_{1/2} = (OR_1)*(OR_2) = \{R / (1 - R)\} / \{A_0 / (1 - A_0)\} \approx \{A / (1 - A)\} / \{A_0 / (1 - A_0)\}$

(2) and: $OR_3 = \mathbf{t'/ s'} \approx \{A_1 / (1 - A_1)\} / \{A / (1 - A)\}$

(3) Based on Prop. (2.3): $OR_3 = \mathbf{t'/ s'} \geq E(\mathbf{z_j}) / E(\mathbf{z_k}) = \mathbf{z_t / z_s} = OR_2$

Because: $\mathbf{t'} \geq \mathbf{s'}$; then: $R_1 \geq R$ # Assumption (A7) & Prop. (2.3)

6.1b. If <u>only</u> Mechanism (1) accounts for the Gx+ enrichment in MS patients.

$P(MS \mid G, Gx+) = P(MS \mid G, Gx-) = P(MS \mid G)$ # Mechanism (2) does not operate

so that: $P(MS \mid G, Gx+, IG_{MS}) = P(MS \mid G, Gx-, IG_{MS}) = P(MS \mid G, IG_{MS})$ # Assumption (A7) & Prop. (2.3)

This second expression is the same as: $\mathbf{s'} = \mathbf{b'}$ or: $\mathbf{s'/b'} = 1$

For example, the data for the <u>HLA partition</u>, yields the estimate of:

$0.97 = \mathbf{s/b} \leq \mathbf{s'/b'} \leq 1.04(\mathbf{s/b}) = 1$ # Eq. (**16**) of Prop. (5.2c) & Table (6)

Thus, most of the DRB1*1501 enrichment in MS must be due to Mechanism (1).

6.1c. If Mechanism (2) accounts for <u>even a portion</u> of the Gx+ enrichment.

$P(MS \mid G, Gx+) > P(MS \mid G) > P(MS \mid G, Gx-)$ # Mechanism (2) does operate

so that: $P(MS \mid G, Gx+, IG_{MS}) > P(MS \mid G, IG_{MS}) > P(MS \mid G, Gx-, IG_{MS})$ # Assumption (A7) & Prop. (2.3)

This second expression is the same as: $\mathbf{s'} < \mathbf{b'}$ or: $\mathbf{s'/b'} < 1$

For the <u>Gender partition</u>, using the Table 6 data, together with Eq. (**16**) of Prop. (5.2c), yields:

$\mathbf{s/b} = 0.27 \leq \mathbf{s'/b'} \leq 1.04(\mathbf{s/b}) = 0.28 < 1$

So that, at least some of the Female enrichment in MS must be due to Mechanism (2).

Proof of Proposition 6.2:

Gender-Status

6.2a. The development of Prop. (4.2) would be unaltered if men and women were to be considered separately. Therefore, from Table (6) it is the case that:

$$\mathbf{t} = P(MS \mid F, IG_{MS}) = 0.183 \; ; \qquad \text{and:} \qquad \mathbf{s} = P(MS \mid M, IG_{MS}) = 0.036$$

Using the data in Tables 2 & 6:

$$P(MS \mid F) = P(F \mid MS)*P(MS) / P(F) \approx (0.68)(0.0015) / (0.5) = 0.00204$$

$$P(MS \mid M) = P(M \mid MS)*P(MS) / P(M) \approx (0.32)(0.0015) / (0.5) = 0.00096$$

Then from Eqs. (**1** & **5**) of Prop. (4), without making any assumptions, it must be the case that:

(4) For women: $(g_1^2)*P(MS \mid F) / \mathbf{t} \leq P(G \mid F) = P(MS, G \mid F) / \mathbf{z_t} \leq (2)*P(MS \mid F) / \mathbf{t}$

(5) And for men: $(g_2^2)*P(MS \mid M) / \mathbf{s} \leq P(G \mid M) = P(MS, G \mid M) / \mathbf{z_s} \leq (2)*P(MS \mid M) / \mathbf{s}$

Substituting into Eqs. (**4** & **5**), the data from Tables (2) & (6), yields:

(6) For women: $(0.00204 / 0.183)*(0.96)^2 = 0.010 \leq P(G \mid F) \leq 0.022 = 2*(0.00204 / 0.183)$

(7) For men: $(0.00096 / 0.036)*(0.90)^2 = 0.022 \leq P(G \mid M) \leq 0.053 = 2*(0.00096 / 0.036)$

See Prop. (6.2d) for an alternative derivation of this relationship and also Eq. (**10**); Prop. (7.1a) for a minor adjustment to these range estimates. The lack of overlap of these predicted ranges indicates that men are more likely than women to be genetically susceptible to getting MS.

Also, because: $P(F) \approx P(M) \approx 0.5$; then, also: $P(M \mid G) \geq P(F \mid G)$

and, thus: $P(G \mid M) \geq P(G) \geq P(G \mid F)$

6.2b1. Moreover, using logic directly analogous to that for Eq. (**6**) in Prop. (4.2a) & Prop. (6.2a) & the Prop. (5.2c) estimates for (g_1) and (g_2), rearrangement of Eqs. (**4** & **5**) yields:

(8) $0.183 / (0.96) = 0.191 \geq \mathbf{t'} \geq P(MS \mid F, G) = \mathbf{z_t} \geq \mathbf{t'}/2 \geq \mathbf{t}/2 = 0.092$

(9) and: $0.036 / (0.90) = 0.040 \geq \mathbf{s'} \geq P(MS \mid M, G) = \mathbf{z_s} \geq \mathbf{s'}/2 \geq \mathbf{s}/2 = 0.018$

Because there is no overlap between these two ranges, we conclude that, for this partition, it must be the case that: $\mathbf{t'} > \mathbf{b'} > \mathbf{s'}$

6.2b2. From Tables (2) & (6), as well as Prop. (6.1a), the odds ratios for the 2nd and 3rd enrichment stages (OR_2 and OR_3) are:

(10) $$OR_2 = \{P(F \mid G, IG_{MS}) / [1 - P(F \mid G, IG_{MS})]\} / \{P(F \mid G) / [1 - P(F \mid G)]\} = \mathbf{z_t / z_s}$$

(11) and: $$OR_3 = \mathbf{t'/s'} \approx \{P(F \mid MS, IG_{MS}) / [1 - P(F \mid MS, IG_{MS})]\} / \{P(F \mid MS) / [1 - P(F \mid MS)]\} = 5.4$$

Using Eqs. (**3**, **10** & **11**), together with the results of Eqs. (**8** & **9**), yields:

$$OR_3 = \mathbf{t'/s'} \approx 5.4 \geq OR_2 = \mathbf{z_t / z_s} \geq (0.092) / (0.040) = 2.3$$ # Eq. (**3**) & Prop. (2.3)

Consequently, from these analyses, we conclude that there is a large penetrance-imbalance for gender, in both the 2nd and 3rd enrichment-stages.

6.2c. For gender (Gx+ = F): $P(G \mid F) \leq P(G)$ # Prop. (6.2a)

Therefore: $P(G \mid F) + P(G- \mid F) = 1 \leq P(G) + P(G- \mid F)$

so that: $P(G- \mid F) \geq P(G-)$ # $1 - P(G) = P(G-)$

Therefore: $P(F, G-) \geq P(G-)*P(F) = \{1 - P(G)\}*P(F)$

Similarly: $P(G- \mid M) \leq P(G-)$

and: $P(M, G-) \leq P(G-)*P(M)$

Therefore: $P(F) = 0.5 \geq P(F, G-) \geq P(G-)*P(F) \geq (1 - 0.022)(0.5) = 0.489$ # Prop (4.2a)

Because: $P(M, G-) + P(F, G-) = P(G-) \geq 0.978$

$P(M) = 0.5 \geq P(M, G-) = P(G-) - P(F, G-) \geq 0.478$

From Prop. (1.2a), it follows that:

$$P(F, G- \mid MS) = \{P(F, G-)*P(MS \mid F, G-)\}/ P(MS) = \{P(F, G-)*P(G- \mid MS)\} / P(G-)$$

and: $$P(M, G- \mid MS) = \{P(M, G-)*P(MS \mid M, G-)\}/ P(MS) = \{P(M, G-)*P(G- \mid MS)\} / P(G-)$$

Therefore: $1 \geq P(M, G- \mid MS) / P(F, G- \mid MS) \geq (0.478) / (0.5) = 0.96$

so that: $P(F, G- \mid MS) \approx P(M, G- \mid MS)$

6.2d. Alternatively, we can use the relationship: $P(G \mid IG_{MS}) = g \geq 0.94$ # Prop. (5.2b)

together with: $P(F, G- \mid IG_{MS}) \approx P(M, G- \mid IG_{MS}) \leq (1 - 0.94)/2 = 0.03$ # Props. (1.2a), (5.2a1) & (6.2c)

to yield: $P(F \mid G, IG_{MS}) = \{P(F \mid IG_{MS}) - P(F, G- \mid IG_{MS})\} / P(G \mid IG_{MS}) \leq (0.68 - 0.03) / 0.94 = 0.69$

Therefore: $0.68 \leq P(F \mid G, IG_{MS}) \leq 0.69$

Using this range estimate for $P(F \mid G, IG_{MS})$, rearranging Eq. (**10**), using the estimate of $(\mathbf{z_t} / \mathbf{z_s})$ from Prop. (6.2b2),

and substituting the values from Tables (2 & 6), yields:

$$\{P(F \mid G) / [1 - P(F \mid G)]\} \geq (0.68 / 0.32) / (5.4) = 0.394$$

and: $$\{P(F \mid G) / [1 - P(F \mid G)]\} \leq (0.69 / 0.31) / (2.3) = 0.967$$

(12) or, with rearrangement: $0.28 \leq P(F \mid G) \leq 0.49$; and: $0.51 \leq P(M \mid G) \leq 0.72$

so that: $(1.04)*P(F \mid G) \leq P(M \mid G) \leq (2.57)*P(F \mid G)$

also: $(0.022)(0.28) / 0.5 = 0.012 \leq P(G \mid F) = P(G)*P(F \mid G) / P(F) \leq (0.022)(0.49) / 0.5 = 0.022$

$(0.022)(0.51) / 0.5 = 0.022 \leq P(G \mid M) = P(G)*P(M \mid G) / P(M) \leq (0.022)(0.72) / 0.5 = 0.032$

(13) and: $(2.3)*P(MS \mid G, M) \leq P(MS \mid G, F) \leq (5.4)*P(MS \mid G, M)$ # Props. (6.1a) & (6.2b2)

6.2e. From Eqs. (**8** & **9**):

$0.092 \leq \mathbf{z_t} \leq \mathbf{t'} \leq 0.191$; and: $0.018 \leq \mathbf{z_s} \leq \mathbf{s'} \leq 0.040$

Because: $\mathbf{t'} = \mathbf{z_t} + (\sigma_{zj}^2) / \mathbf{z_t}$; and: $\mathbf{s'} = \mathbf{z_s} + (\sigma_{zk}^2) / \mathbf{z_s}$ # Prop. (2.1)

therefore: $\sigma_{zj}^2 = (\mathbf{t'} - \mathbf{z_t})(\mathbf{z_t})$; and: $\sigma_{zk}^2 = (\mathbf{s'} - \mathbf{z_s})(\mathbf{z_s})$

(14) and, thus: $0 \leq \sigma_{zj}^2 \leq 0.009$; and: $0 \leq \sigma_{zk}^2 \leq 0.0004$

or, equivalently: $\sigma_{zj} \leq 0.095$; and: $\sigma_{zk} \leq 0.02$

Proof of Proposition 6.3:

HLA-Carrier Status

6.3a. Again, because the development of Prop 4.2 is unaffected by considering HLA+ and HLA– individuals separately, therefore, we can define (see Table 5) the quantities:

$$\mathbf{t} = P(MS \mid HLA+, IG_{MS}) = 0.139$$

and: $$\mathbf{s} = P(MS \mid HLA–, IG_{MS}) = 0.129$$

Using the data in Tables (2) & (5):

$$P(MS \mid HLA+) = P(HLA+ \mid MS)*P(MS) / P(HLA+) = (0.55)(0.0015) / (0.24) = 0.0034$$

$$P(MS \mid HLA–) = P(HLA– \mid MS)*P(MS) / P(HLA–) = (0.45)(0.0015) / (0.76) = 0.0009$$

Therefore, from Eqs. (**1** & **5**) of (Prop. 4.2a), even without Assumptions (A7), it must be the case that:

For HLA+: $(g_1^2)*P(MS \mid HLA+) / \mathbf{t} \leq P(G \mid HLA+) \leq (2)*P(MS \mid HLA+) / \mathbf{t}$

And for HLA–: $(g_2^2)*P(MS \mid HLA–) / \mathbf{s} \leq P(G \mid HLA–) \leq (2)*P(MS \mid HLA–) / \mathbf{s}$

Substituting into these equations the data from Tables 2 & 6, yields:

(15) For (HLA+): $(0.0034 / 0.139)*(0.97)^2 = 0.023 \leq P(G \mid HLA+) \leq (2)(0.0034 / 0.139) = 0.049$

(16) For (HLA–): $(0.0009 / 0.129)*(0.90)^2 = 0.0057 \leq P(G \mid HLA–) \leq (2)(0.0009 / 0.129) = 0.014$

See also Eq. (**10**); Prop. (7.1a) for an adjustment to these range estimates.

Again, the lack of any overlap between these predicted ranges, indicates that HLA+ individuals are more likely than HLA– individuals to be genetically-susceptible to MS.

6.3b. The observations from Tables (2) & (5) for the HLA partition (Gx+ = HLA+) also support this notion.

Thus $P(HLA+ \mid IG_{MS}) = A = 0.55 \approx 0.57 = A_1 = P(HLA+ \mid MS, IG_{MS})$ # Tables (2) & (5)

where: $A \approx R$; and: $A_1 \approx R_1$; so that, for this partition: $\mathbf{t'} \approx \mathbf{b'} \approx \mathbf{s'}$ # Props. (1.5) & (5.2b)

Also from Tables (2 & 5), the OR for the 3rd enrichment-stage (OR_3) is:

$$OR_3 = \{P(HLA+ \mid MS, IG_{MS}) / [1-P(HLA+ \mid MS, IG_{MS})]\}/\{P(HLA+ \mid MS) / [1-P(HLA+ \mid MS)]\} = 1.06$$

Thus, there is little or no discernable penetrance-imbalance in the 3rd enrichment-stage for HLA-carrier status.

From Prop. (2.3): $1 \leq OR_2 \leq 1.06$; or: $\mathbf{z_t} \approx \mathbf{z_s} \approx \mathbf{z}$

Together with ($g \geq 0.94$) from Prop. (5.2b) and substituting the values from Tables (2) & (5) yields:

$$1 \leq \{P(HLA+ \mid G) / [1 - P(HLA+ \mid G)]\} \leq \{(0.55 / 0.45) / 1.06\} = 1.15$$

or, with rearrangement: $0.54 \leq P(HLA+ \mid G) \leq 0.55$; and: $0.45 \leq P(HLA- \mid G) \leq 0.46$

$$0.050 = (0.022)(0.54) / 0.24 \leq P(G \mid HLA+) = P(G)*P(HLA+ \mid G) / P(HLA+) \leq (0.022)(0.55) / 0.24 = 0.050$$

$$0.013 = (0.022)(0.45) / 0.76 \leq P(G \mid HLA-) = P(G)*P(HLA- \mid G) / P(HLA-) \leq (0.022)(0.44) / 0.75 = 0.013$$

Also: $P(G \mid HLA+) = \{[P(HLA-) / P(HLA+)]*[P(HLA+ \mid G) / P(HLA- \mid G)]\}*P(G \mid HLA-)$

Thus: $P(G \mid HLA+) \geq (0.76 / 0.24)*(0.54/ 0.46)*P(G \mid HLA-) = (3.72)*P(G \mid HLA-)$

and: $P(G \mid HLA+) \leq (0.76 / 0.24)*(0.55/ 0.45)*P(G \mid HLA-) = (3.87)*P(G \mid HLA-)$

Also: $P(MS \mid G, HLA-) \leq P(MS \mid G, HLA+) \leq (1.06)*P(MS \mid G, HLA-)$ # Prop. (6.1a)

This confirms that the vast majority of the enrichment of HLA+ status in MS results from Mechanism (1).

Also, if gender and HLA status are either independent or if: $P(F \mid G, HLA+) \geq P(F \mid G)$

Then, the prevalence of HLA+ women is expected to rise at each enrichment stage, so that:

$P(F \mid G, HLA+) \geq 0.28$ # Eq. (**11**)

$P(F \mid G, HLA+, MZ_{MS}) \geq 0.68$ # Table (2)

and: $P(F \mid G, HLA+, MS, MZ_{MS}) \geq 0.92$ # Table (2)

6.3c. Homozygous DRB1*1501-Status

Compared to individuals who lack the DRB1*1501 allele (HLA–), there is an enrichment of individuals who are homozygous for this allele (2HB+) in an MS population, and this enrichment is much greater than it is for individuals who carry one copy of this allele (1HB+) and one copy of a “non-DRB1*1501” allele (1HB–). This can be appreciated from Table 3, where the ORs in these circumstances are:

(17) $OR_{2HB+} = OR_{1/2} = 9.3 - 10.4$ # Comparing (2HB+) to (HLA–)

$OR_{1HB+} = OR_{1/2} = 3.1 - 3.6$ # Comparing (1HB+) to (HLA–)

Notably: $P(2HB+ \mid HLA+, IG_{MS}) = 0.18$ # Table (3)

From Tables 2 & 5: $P(HLA+ \mid MS, IG_{MS}) = 0.57$; and: $P(HLA+ \mid MS) = 0.55$

For illustrative purposes, we will assume that, all of the enrichment occurs via Mechanism (2) and is due to a penetrance imbalance in the (2HB+, G) subset. Assigning the factor (**v**) to represent this additional enrichment, and because the subsets (1HB+, G) and (2HB+, G) partition the set (HLA+, G), therefore:

(18) $$P(MS \mid 2HB+, HLA+, G, IG_{MS}) = P(MS \mid 2HB+, G, IG_{MS})$$

$$= (\mathbf{v})*P(MS \mid G, 1HB+, IG_{MS}) = (\mathbf{v})*P(MS \mid G, HLA-, IG_{MS})$$

(19) $$P(MS, HLA+ \mid G, IG_{MS}) = P(HLA+ \mid G, IG_{MS})*P(MS \mid HLA+, G, IG_{MS})$$

(20) $$P(MS \mid HLA+, G, IG_{MS}) = P(MS, 2HB+ \mid HLA+, G, IG_{MS}) + P(MS, 1HB+ \mid HLA+, G, IG_{MS})$$

$$= P(2HB+ \mid HLA+, G, IG_{MS})*P(MS \mid 2HB+, G, IG_{MS})$$

$$+ P(1HB+ \mid HLA+, G, IG_{MS})*P(MS \mid 1HB+, G, IG_{MS})$$

So that, using the relationships of Eq. (**18**) yields:

(21) $$P(MS \mid HLA+, G, IG_{MS}) = \{(\mathbf{v})*P(2HB+ \mid HLA+, G, IG_{MS}) + P(1HB+ \mid G, IG_{MS})\}*P(MS \mid HLA-, G, IG_{MS})$$

Using the results of Prop. (5.2b), which indicates that: $P(MS, G) \approx P(MS)$

And substituting into Eq. (**21**), the observed values of:

$P(2HB+ \mid HLA+, G, IG_{MS}) = 0.18$; and: $P(1HB+ \mid HLA+, G, IG_{MS}) = 0.82$ # Table (3)

and using Eq. (**20**) yields:

(22) $$P(MS, HLA+ \mid G, IG_{MS}) = \{(0.18)\mathbf{v} + 0.82\}*P(HLA+ \mid G, IG_{MS})*P(MS \mid HLA-, G, IG_{MS})$$

(23) also: $P(MS, HLA{-} \mid G, IG_{MS}) \;=\; P(HLA{-} \mid G, IG_{MS})*P(MS \mid HLA{-}, G, IG_{MS})$

and: $P(MS \mid G, IG_{MS}) \;=\; \{[(0.18)\mathbf{v} + 0.82]*P(HLA{+} \mid G, IG_{MS}) + P(HLA{-} \mid G, IG_{MS})\}*P(MS \mid HLA{-}, G, IG_{MS})$

Therefore, using Eqs. (**22** & **23**) & Prop. (5.2b) & Table 5, yields:

(24) $P(HLA{+} \mid MS, G, IG_{MS}) \;=\; P(MS, HLA{+} \mid G, IG_{MS}) / P(MS \mid G, IG_{MS})$

$$= \{[(0.18)\mathbf{v} + 0.82]*(0.55)\} / \{[(0.18)\mathbf{v} + 0.82]*(0.55) + 0.45\} \;=\; 0.57$$

Solving Eq. (**24**) for (**v**) yields: $\mathbf{v} = P(MS \mid 2HB{+}, IG_{MS}) / P(MS \mid 1HB{+}, IG_{MS}) = 1.47$

or, from Eq. (**18**) and Prop. (2.3), equivalently:

$$\mathbf{v} = P(MS \mid 2HB{+}, G, IG_{MS}) / P(MS \mid HLA{-}, G, IG_{MS}) \;=\; \mathbf{t'/s'} \;=\; OR_3 \;=\; 1.47 \;\geq\; OR_2$$

Thus, as is the case for carrier-status, the large majority (possibly all) of the enrichment ($OR_{1/2} \geq 9.3$), which takes place in the 2HB+ subset during the first and second enrichment stages, occurs via Mechanism (1).

Consequently, the fact that: $R_1 \approx R$ # See above; Prop. (6.3b)

Suggests that, even for the partition (Gx+ = 2HB+): $\mathbf{t'} \approx \mathbf{b'} \approx \mathbf{s'}$

Defining: $\mathbf{z_{2HB+}} \;=\; P(MS \mid G, 2HB{+})$

$\mathbf{z_{1HB+}} \;=\; P(MS \mid G, 1HB{+})$

and: $\mathbf{z_{HLA-}} \;=\; P(MS \mid G, HLA{-})$

Then, from Prop. (2.3), Eq. (**18**), and by convention:

$$\mathbf{t'/s'} \;\geq\; \mathbf{z_{2HB+}} / \mathbf{z_{HLA-}} \;=\; \mathbf{z_{2HB+}} / \mathbf{z_{1HB+}} \;\geq\; 1$$

(25) Therefore, because: $\mathbf{t'/s'} \approx 1$; then, also: $\mathbf{z_{2HB+}} \approx \mathbf{z_{1HB+}} = \mathbf{z_{HLA-}} \approx \mathbf{z}$

From Prop. (5.2b), and using the data in Tables 2:

$$P(MS \mid 2HB{+}) \;\approx\; P(MS, G \mid 2HB{+}) \;=\; P(2HB{+} \mid MS, G)*P(MS, G) / P(2HB{+})$$

so that: $P(MS \mid 2HB{+}) \;\approx\; (0.10)(0.0015) / (0.0.016) = 0.00938$

Also: $\mathbf{z_{2HB+}} = P(MS, G \mid 1HB{+}) \;=\; P(1HB{+} \mid MS, G)*P(MS, G) / P(1HB{+})$

so that: $P(MS \mid 1HB{+}) \;\approx\; (0.45)(0.0015) / (0.224) = 0.00301$

(26) Therefore: $P(G \mid 2HB+) = P(MS, G \mid 2HB+) / \mathbf{z_{2HB+}}$

(27) and: $P(G \mid 1HB+) = P(MS, G \mid 1HB+) / \mathbf{z_{1HB+}}$

Following the logic of Prop. (4.2b) and Eq. (**25**), therefore, again:

$$0.067 \leq \mathbf{z_{2HB+}} \approx \mathbf{z_{1HB+}} \leq \mathbf{b'} \leq (0.134) / (0.94) = 0.143$$

Substituting these ranges into Eqs. (**26** & **27**) yields

For 2HB+: $(0.00938) / (0.143) = 0.066 \leq P(G \mid 2HB+) \leq (0.00938) / (0.067) = 0.140$

And for 1HB+: $(0.00301) / (0.143) = 0.021 \leq P(G \mid 1HB+) \leq (0.00301) / (0.067) = 0.045$

Using a more refined estimate for (**z**) of:

$0.067 \leq \mathbf{z} \leq 0.089$ # From Eq. (**8**) of Prop. (7.1a); Section E

yields: $0.110 \leq P(G \mid 2HB+) \leq 0.140$

and: $0.036 \leq P(G \mid 1HB+) \leq 0.045$

<u>Proof of Proposition 6.4</u> <u>Hardy-Weinberg Considerations</u>

6.4a. Alternatively, we can analyze the impact of DRB1*1501 status on MS using a Hardy-Weinberg Equilibrium (HWE) approach. We will consider a population in HWE with respect to a particular gene (which has one of two possible allelic states) and with each allelic state having a specific mean allelic frequency (MAF). In this section, these two states are distinguished by (p) and (q), where (p) is the MAF of the first state and (q) is the MAF of the second. {NB: elsewhere in this paper (p), and (q) have different meanings.}

Thus: $p = P(\text{State 1})$; $q = P(\text{State 2})$; and: $p + q = 1$ # (State 1) and (State 2) form a partition

In this case, the three genotypes (in combination) at equilibrium are represented by:

$$(p + q)^2 = p^2 + 2pq + q^2 = 1$$

where: $p^2 = P(\text{homozygous; State 1/ State 1})$

$2pq = P(\text{heterozygous; State 1/ State 2})$

and: $q^2 = P(\text{homozygous; State 2/ State 2})$

We can then apply a selection pressure to perturb this equilibrium state. We define absolute fitness levels for the different genotypes $\{(W_p), (W_{pq}), \text{and } (W_q)\}$ such that the make-up of the selected, next generation, population is:

$$(W_p)p^2 + (W_{pq})2pq + (W_q)q^2$$

Thus, the new MAFs (p' and q') in the next generation, which results from this applied selection, will be:

(28) $$p' = \{(W_p)p^2 + (½)(W_{pq})2pq\} / \{(W_p)p^2 + (W_{pq})2pq + (W_q)q^2\}$$

(29) and: $$q' = \{(W_q)q^2 + (½)(W_{pq})2pq\} / \{(W_p)p^2 + (W_{pq})2pq + (W_q)q^2\}$$

where, by this definition: $p' + q' = 1$

Eqs. (**28** & **29**) can be re-expressed by defining (X) as:

$$X = (W_p)p^2 + (W_{pq})2pq + (W_q)q^2$$

and by defining normalized fitness levels for the different genotypes $\{(w_p), (w_q), \text{and } (w_{pq})\}$ as :

$$w_p = W_p / X \;;\quad w_q = W_q / X \;;\quad \text{and:}\quad w_{pq} = W_{pq} / X$$

In which case: $p' = (w_p)p^2 + (½)(w_{pq})2pq$

and: $q' = (w_q)q^2 + (½)(w_{pq})2pq$

If, after the selection process, the resultant population is still in HWE, then it must be the case that:

(31) $$(p' + q')^2 = (p')^2 + 2(p')(q') + (q')^2 = (w_p)p^2 + (w_{pq})2pq + (w_q)q^2$$

(30) In which case: $p' = (w_p)^{½}\,p$; $q' = (w_q)^{½}\,q$; and: $(w_{pq}) = (w_p)^{½}(w_q)^{½}$

Otherwise, the resulting population will not be at HWE.

6.4b. This suggests a method for further exploring the impact of a genetic trait (Gx+) on the development of MS. Thus, by analogy to HWE (Prop. 6.4a), we can consider the development of MS as a selection process with a different "fitness" for each genotype. In the circumstanaces of DRB1*1501, the three genotypes are:

1. Homozygous DRB1*1501 ; or: (2HB+) or: (1HB+, 1HB+)

2. Heterozygous DRB1*1501 ; or: (1HB+) or: (1HB+, 1HB–)

and: 3. Homozygous "non-DRB1*1501" ; or: (HLA–) or: (1HB–, 1HB–)

In the analogy, for a general population (at HWE) where: P(HLA+) = 0.24 ; therefore:

(32) $p^2 = P(2HB+) = 0.016$; $2pq = P(1HB+) = 0.224$; and: $q^2 = P(HLA–) = 0.76$

In the general population, these genotypes are presumed to be in HWE and, in fact, for the UCSF #2 control population, this presumption is supported by the data (Table 3). In addition:

$$1 \approx P(G \mid MS) = P(2HB+, G \mid MS) + P(1HB+, G \mid MS) + P(HLA–, G \mid MS)$$ # Prop. (5.2b)

where, from Eq. (**31**) and Table (2):

$$P(2HB+, G \mid MS) = \{(p^2)*P(G \mid 2HB+)*P(MS \mid 2HB+, G)\} / P(MS) \approx 0.10$$

$$P(1HB+, G \mid MS) = \{(2pq)*P(G \mid 1HB+)*P(MS \mid 1HB+, G)\} / P(MS) \approx 0.45$$

and: $$P(HLA–, G \mid MS) = \{(q^2)*P(G \mid HLA–)*P(MS \mid HLA–, G)\} / P(MS) \approx 0.45$$

Consequently $$w_p = \{P(G \mid 2HB+)*P(MS \mid 2HB+, G)\}/ P(MS) = P(G \mid MS)*P(MS, G \mid 2HB+) / P(MS, G)$$

$$w_{pq} = \{P(G \mid 1HB+)*P(MS \mid 1HB+, G)\}/ P(MS)$$

$$w_q = \{P(G \mid HLA–)*P(MS \mid HLA–, G)\}/ P(MS)$$

Based on the data in Table 3, each of the MS populations studied are either at or very near to HWE with respect to DRB1*1501 status, even though this HWE is (in all cases) a very different one from that of the control populations. Therefore, based on Eqs. (**30**–**32**), this yields the relationship that:

$$(w_p)p^2 + (w_{pq})2pq + (w_q)q^2 = p'^2 + 2p'q' + q'^2 = 1$$

(33) and, thus: $$w_p = P(MS \mid 2HB+) / P(MS) = P(2HB+ \mid MS) / P(2HB+) = (p'/ p)^2$$

Similarly: $$w_q = P(MS \mid HLA–) / P(MS) = P(HLA– \mid MS) / P(HLA–) = (q'/ q)^2$$

Thus, for a population at HWE, the quantity $(w_p)^{½}$ estimates the relative MAF of the risk allele in the susceptible MS population compared to its MAF in the general population. Accepting the conclusion that:

$$P(MS, G) \approx P(MS)$$ # Prop. (5.2b)

Then, this relative MAF, in turn, represents the entire enrichment (OR_1 and OR_2) that occurs when moving, first, from the general population to the (G) population and then, second, from the (G) population to the (MS, G) population. In addition, the ratios of these "fitness" levels represent the relative enrichment of the different genotypes when moving from the general population to the (MS) population. For example, comparing the relative enrichment of (2HB+) compared to (HLA–), yields:

(34) $$\{P(G \mid 2HB+)*P(MS \mid 2HB+, G)\} / \{P(G \mid HLA–)*P(MS \mid HLA–, G)\} \approx (w_p) / (w_q)$$

Moreover, because, based on the many considerations of Props. (6.3a – 6.3d), & Eq. (**25**), it seems

to be the case that: $$P(MS \mid G, 2HB+) \approx P(MS \mid G, 1HB+) \approx P(MS \mid G, HLA–)$$

(35) and, therefore, that: $$(w_p) / (w_q) \approx P(G \mid 2HB+) / P(G \mid HLA–)$$

We will assume that this approximate equality is a true equality and refine our nomenclature such that:

$P(HB+ \mid G) = p' =$ MAF of the DRB1*1501 allele in the susceptible population

and: $P(HB– \mid G) = q' =$ combined MAF of "non-DRB1*1501 alleles" in the susceptible population

In the context of DRB1*1501 (Table 3), we take the <u>independent selection</u> of these alleles to imply that:

$$P(1HB+, 1HB– \mid G) = 2*P(HB+ \mid G)*P(HB– \mid G) = 2\{(w_p)^{½} p\}*\{(w_q)^{½} q\}$$

and: $$P(1HB+, 1HB+ \mid G) = \{P(HB+ \mid G)\}^2 = (p')^2 = w_p (p^2) ;$$

and: $$P(1HB–, 1HB– \mid G) = \{P(HB– \mid G)\}^2 = (q')^2 = w_q (q^2)$$

Applying these weights to Eq. (**31**) yields:

(36) $$(w_p)p^2 + (w_{pq})2pq + (w_q)q^2 = (w_p)p^2 + (w_p)^{½} (w_q)^{½} 2pq + (w_q)q^2 = \{p(w_p)^{½} + q(w_q)^{½}\}^2 = 1$$

Defining (w) by the relationship: $w = (w_p / w_q)^{½} > 1$; we can transform Eq. (**36**) to yield:

$$q^2 + (w)*2pq + (wp)^2 = (w^0)*q^2 + (w^1)*2pq + (w^2)*p^2 = (q + wp)^2 = 1/ w_q$$

For convienience, we can then define "apparent" initial probabilities for the different genotypes as:

$$P(HLA–)^{app} = (w_q)*P(HLA–) ; \quad P(1HB+)^{app} = (w_q)*P(1HB+)$$

and: $$P(2HB+)^{app} = (w_q)*P(2HB+)$$

So that, the relative proportions of genotypes in the susceptible population can be represented as:

$$P(HLA– \mid G) = P(HLA–)^{app} ; \quad P(1HB+ \mid G) = (w)*P(1HB+)^{app}$$

and: $$P(2HB+ \mid G) = (w^2)*P(2HB+)^{app}$$

Moreover, from Eq. (**25**): $P(MS \mid G, 2HB+) \approx P(MS \mid G, 1HB+) \approx P(MS \mid G, HLA–)$

Thus, the relative proportions of genotypes in the MS population can also be represented as:

$$P(HLA– \mid MS) = P(HLA–)^{app} ; \quad P(1HB+ \mid MS) = (w)*P(1HB+)^{app}$$

and: $$P(2HB+ \mid MS) = (w^2)*P(2HB+)^{app}$$

It is in this sense that the two DRB1*1501 alleles are said to be <u>independently selected</u>; that is the relative normalized selection pressure for two alleles (w^2) is equal to the square of that for one allele (w).

Thus, the weighting scheme implied here is geometric (1, w, w^2) for the homozygous-lack, and for the heterozygous- and homozygous-presence, of the risk allele. This is analogous to the joint probability of two events being the product of the individual probabilities; and it contrasts to the weighting scheme for recessive and dominant traits, assuming a non-zero risk for non-carriers and a suitable definition of ($w > 1$), which would be (1, 1, w) and (1, w, w), respectively. Moreover, because the arguments made above are fully reversible, the initial and final populations will be in HWE if, and only if, the selection pressure is geometric. Consequently, if both initial and resulting populations (following strong selection) are at HWE (Tables 2 – 4), this implies that, for some (w_p) and (w_q), Eq. (**36**) holds. Furthermore, a geometric scheme for DRB1*1501 implies that the selection is occurring at the level of the allele; not the genotype. Thus, each DRB1*1501 allele is being <u>independently selected</u> to produce genetic-susceptibility.

This suggests that each 1501 allele contributes equally to the total number of susceptibility alleles needed to produce susceptibility.[27] For example, if on average, susceptible non-DRB1*1501 genotypes have ten susceptibility alleles, susceptible genotypes with one DRB1*1501 allele might have only nine, whereas susceptible genotypes with two such alleles might have only eight.[27]

6.4c. As a result, we can calculate these HWE weights for the DRB1*1501 allele directly from observed data. For example, using the independent Canadian and UCSF #2 samples, both of which include <u>observed</u> cases and <u>observed</u> controls (Table 3), and in conjunction with and Eqs. (**31**, **33** & **36**), we estimate that:

For Canada:

$$w_p = (0.329 / 0.128)^2 = 6.59$$ # Assuming cases & controls are in HWE

$$w_q = (0.671 / 0.872)^2 = 0.59$$

$$w_{pq} = (w_p)^{1/2} (w_q)^{1/2} = 1.98$$

For UCSF #2:

$$w_p = (0.269 / 0.104)^2 = 6.69$$ # Using the actual data for cases & controls

$$w_q = (0.731 / 0.896)^2 = 0.67$$

$$w_{pq} = (w_p)^{1/2} (w_q)^{1/2} = 2.01$$

Averaging these two experiences yields:

(37) $$w_p = P(G \mid 2HB+) * P(MS \mid 2HB+, G) = (6.64 / 0.63)(w_q) = (10.5)(w_q)$$

Using Eqs. (**25**) & (**34**), this yields:

$$w_p = P(G \mid 2HB+) = (10.5)(w_q)$$

so that: $(w_p) / (w_q) = P(G \mid 2HB+) / P(G \mid HLA–) = 10.5$

Therefore, despite a very strong selection pressure, the large majority of DRB1*1501 genotype selection seems to occur when moving from the general population to the susceptible (G) population (the OR_1 step) and very little selection seems to occur when moving from the set (G) to the set (MS, G) – i.e., during the (OR_2) step. Moreover, the fact that the initial set (general population) and final set (MS, G) are at HWE, almost certainly, means that the intermediate set (G) is also at HWE.

6.4d. In addition, for each of these samples, for men and women (considered separately), the observed proportions of cases in the different HLA-categories are very near to those expected at HWE (Tables 3 & 4). Despite this, however, men consistently have a lower odds ratios for MS in all HLA+ categories, a smaller proportion of (2HB+, MS) patients, and a lower probability for P(HLA+ | MS) compared to women (Tables 2 & 5). For example, undertaking the same analysis as in Prop. (6.4c) yields:

For women: Canada:

$w_p = (0.367 / 0.128)^2 = 8.22$ # Assumes cases & controls in HWE

$w_q = (0.633 / 0.872)^2 = 0.53$

$w_{pq} = (w_p)^{½}(w_q)^{½} = 2.08$

UCSF #2

$w_p = (0.290 / 0.104)^2 = 7.79$ # Actual data for cases & controls

$w_q = (0.710 / 0.896)^2 = 0.63$

$w_{pq} = (w_p)^{½}(w_q)^{½} = 2.21$

For men: Canada:

$w_p = (0.307 / 0.128)^2 = 6.59$ # Assumes cases & controls in HWE

$w_q = (0.693 / 0.872)^2 = 0.63$

$w_{pq} = (w_p)^{½}(w_q)^{½} = 1.91$

UCSF #2

$w_p = (0.214 / 0.104)^2 = 4.43$ # Actual data for cases & controls

$w_q = (0.786 / 0.896)^2 = 0.77$

$w_{pq} = (w_p)^{½}(w_q)^{½} = 1.81$

Thus, the observed differences between men and women with MS indicate that men (compared to women) have a smaller MAF for the DRB1*1501 allele in an MS population, which is reflected in the consistent observation from Table 2 that:

(38) $$P(HLA+ \mid MS, F, G) > P(HLA+ \mid MS, M, G)$$

To evaluate the possible bases for this observation we will consider the following relationships:

(39) $$P(MS, F \mid G) = P(F \mid G)*P(MS \mid F, G)$$
$$= \{P(MS \mid F, G) / P(MS \mid M, G)\}*\{P(F \mid G) / P(M \mid G)\}*P(MS, M \mid G)$$

(40) also: $$P(HLA+ \mid MS, F, G) = P(MS, F, HLA+ \mid G) / P(MS, F \mid G)$$

(41) and: $$P(HLA+ \mid MS, M, G) = P(MS, M, HLA+ \mid G) / P(MS, M \mid G)$$

(42) and: $$P(MS, M, HLA+ \mid G) = P(M \mid G)*P(HLA+ \mid M, G)*P(MS \mid M, G, HLA+)$$

Consequently, dividing Eq. (**40**) by Eq. (**41**) yields:

(43) $$P(HLA+ \mid MS, F, G) / P(HLA+ \mid MS, M, G)$$
$$= \{P(MS, M \mid G) / P(MS, F \mid G)\}*\{P(MS, F, HLA+ \mid G) / P(MS, M, HLA+ \mid G)\}$$

Breaking down the RHS of Eq. (**43**) into its two component parts and substituting into these equations the relationships derived from Eqs. (**39**–**42**) yields:

(44) First: $$P(MS, M \mid G) / P(MS, F \mid G) = \{P(M \mid G) / P(F \mid G)\}*\{P(MS \mid M, G) / P(MS \mid F, G)\}$$

(45) Second: $$P(MS, F, HLA+ \mid G) / P(MS, M, HLA+ \mid G) =$$
$$\{(P(F \mid G) / P(M \mid G)\}*$$
$$\{P(HLA+ \mid F, G) / P(HLA+ \mid M, G)\}*$$
$$\{P(MS \mid F, G, HLA+) / P(MS \mid M, G, HLA+)\}$$

Then, multiplying Eqs (**44** & **45**) and substituting back into Eq. (**43**), yields:

$$P(HLA+ \mid MS, F, G) / P(HLA+ \mid MS, M, G) = \{P(HLA+ \mid F, G) / P(HLA+ \mid M, G)\} * \{P(MS \mid F, G, HLA+) / P(MS \mid M, G, HLA+)\} / \{P(MS \mid F, G) / P(MS \mid M, G)\}$$

Thus, as noted earlier, there are again only two possible mechanisms to explain the relationship of Eq. (**38**). The first is a MAF effect or:

1. $P(HLA+ \mid F, G) > P(HLA+ \mid M, G)$

and the second is a penetrance effect or:

2. $P(MS \mid F, G, HLA+) / P(MS \mid M, G, HLA+) > P(MS \mid F, G) / P(MS \mid M, G)$

Of these, the conclusions of Props. (6.3a – 6.3d) clearly favor mechanism (1), so that the principle basis for Eq. (**38**) can most easily be ascribed to the fact that men (compared to women) have a smaller MAF for the DRB1*1501 allele in the susceptible population (i.e., in the subset G).

Appendix S1; Section E

Refining the Parameter Estimates

Propositions

Proposition 7 .... p. 1

Proposition 8 .... p. 5

Large Red Rectangles above represent hyperlinks to main parts of Section E

Small Red Boxes within Document represent hyperlinks within Appendix S1.

Small Green Boxes within Appendix S1 are hyperlinks back to Main Text

Navigate Back to Main Menu

Proposition 7: **1.** **a.** $0.067 \leq P(MS \mid G) = \mathbf{z} \leq 0.089$

b. $0.016 \leq P(G) \leq 0.022$

c. $0.030 \leq P(MS \mid M, G) \leq 0.040$; and: $0.096 \leq P(MS \mid F, G) \leq 0.191$

2. $P(G3 \mid G) \approx 0$

New Definitions and Relationships for Proposition 7:

1. (G0), (G3) = Mutually exclusive sets of genetically-susceptible individuals who either depend upon (G0) or don't depend upon (G3) environmental events to produce MS. (G0) + (G3) = (G)

2. $P(MS, E \mid G3) = P(MS \mid E, G3)*P(E \mid G3) = P(MS \mid G3)*P(E)$ # See Section B

Assumption:

A8. $(G3) \subset (G1)$; or, equivalently: $P(G1 \mid G3) = 1$

Proof of Proposition 7.1:

7.1a From # Props. (2.1 & 5.2c) and Table 6, it follows that:

$$P(MS \mid M, G) = \mathbf{z_s} \leq P(MS \mid M, G, IG_{MS}) = P(MS \mid M, IG_{MS}) / g_2 \leq 0.036 / 0.90 = 0.040$$

and: $$P(MS \mid F, G) = \mathbf{z_t} \leq P(MS \mid F, G, IG_{MS}) = P(MS \mid F, IG_{MS}) / g_1 \leq 0.183 / 0.96 = 0.191$$

(1) In addition: $P(MS \mid G) = \mathbf{z} = P(M \mid G)*P(MS \mid M, G) + P(F \mid G)*P(MS \mid F, G)$

From Eq. (**13**) of Prop. (6.2d), predicted ranges provide the following boundary conditions:

(2) at the lower-bound: $P(MS \mid F, G) = \mathbf{z_t} = (2.3)*P(MS \mid G, M)$; and: $P(M \mid G) = 0.51$

(3) at the upper-bound: $P(MS \mid F, G) = \mathbf{z_t} = (5.4)*P(MS \mid G, M)$; and: $P(M \mid G) = 0.72$

Substituting these boundary conditions back into Eq. (**1**) yields the boundaries:

(4) at the lower-bound: $\mathbf{z} = (0.51)*(0.040) + (0.49)*(2.3)*(0.040) = 0.065$

(5) and at the upper-bound: $\mathbf{z} = (0.72)*(0.040) + (0.28)*(5.4)*(0.040) = 0.089$

However, the Eq. (**4**) lower boundary for (**z**) is inconsistent with the earlier conclusion that:

$\mathbf{z} \geq 0.067$ # From Eq. (**7**); Prop. (4.2b); Section C

To resolve this discrepancy, we will define (a_1) such that: $P(MS \mid G, F) / P(MS \mid G, M) = a_1$

To make these two analyses "coherent", requires the lower boundary-estimate to be:

(6) $\mathbf{z} = \{1 - P(F \mid G)\}*(0.040) + P(F \mid G)*(a_1)*(0.040) = 0.067$

(7) where: $\{P(F \mid G) / [1 - P(F \mid G)]\} = (0.69 / 0.31) / (a_1)$ # Prop. (6.2d)

Solving Eqs. (**6** & **7**) for (a_1) & P(F|G) yields: $a_1 = 2.4$; and: P(F|G) = 0.48

so that the lower-boundary is: $\mathbf{z} = (0.52)*(0.040) + (0.48)*(2.4)*(0.040) = 0.067$

Thus, using these new boundaries make our other estimates "coherent" requires that:

(8) $0.067 \leq \mathbf{z} \leq 0.089$ # Eq. (**5**) & Prop. (4.2b)

(9) 0.28 ≤ P(F|G) ≤ 0.48 ; and: 2.4 ≤ P(MS|G, F) / P(MS|G, M) ≤ 5.4 # Prop. (6.2b)

(10) Also, combining Eqs. (**8** & **9**) with the estimates from Props. (6.2a) & (6.3b), yield:

0.010 ≤ P(G|F) ≤ 0.021 ; and: 0.023 ≤ P(G|M) ≤ 0.032

and: 0.044 ≤ P(G|HLA+) ≤ 0.049 ; and: 0.012 ≤ P(G|HLA–) ≤ 0.014 # $\mathbf{z_t} \approx \mathbf{z_s} \approx \mathbf{z}$

7.1b. In addition, the range-estimate for (**z**) given by Eq. (**8**), also requires other range-estimates to be adjusted to make them "coherent" with each other. Thus, because:

$(g)*P(MS) / \mathbf{z}_{max} \leq P(G) \leq 2*(1.86)*\{P(MS) / P(MS | MZ_{MS})\}$ # Eq. (**13**); Prop. (4.2c)

(11) Therefore: $(0.94)(0.0015 / 0.089) = 0.016 \leq P(G) \leq 2*(1.86)*\{0.0015 / 0.25) = 0.022$

Also, because from Prop. (4.2b): $\sigma_{zi}^2 = (\mathbf{b'} - \mathbf{z})*(\mathbf{z})$

(12) So that: $0.0040 = (0.134 - 0.089)*(0.089) \leq \sigma_{zi}^2 \leq (0.143 - 0.067)*(0.067) = 0.0051$

and, therefore: $0.063 \leq \sigma_{zi} \leq 0.071$

7.1c. Using the new "coherent" ranges of Eqs. (**8**–**11**), together with Eq. (**3**), yields:

(13) $(0.72)(\mathbf{z_s}) + (0.28)(5.4)(\mathbf{z_s}) \geq 0.067$; or: $\mathbf{z_s} \geq 0.030$

Also, from Eq. (**9**) of Prop. (6.2b): $\mathbf{z_s} \leq 0.040$

(14) Combining these estimates yields: $0.030 \leq P(MS | M, G) = \mathbf{z_s} \leq 0.040$

This also leads to the lower-boundary condition that:

(15) $(0.52)(0.04) + (0.48)(\mathbf{z_t}) \geq 0.067$; or: $\mathbf{z_t} \geq 0.096$

Also, from Eq. (**8**) of Prop. (6.2b): $\mathbf{z_t} \leq 0.191$

(16) Combining these estimates yields: $0.096 \leq P(MS | F, G) = \mathbf{z_t} \leq 0.191$

Proof of Proposition 7.2:

7.2a. Because "purely genetic" MS is defined to be independent of the environment (see also Section B), it's penetrance is expected to very high (i.e., near unity) and, thus, we anticipate both that:

(17) $P(MS \mid G3) \approx 1$; and also that: $(G3) \subset (G1)$ # Assumption (A8)

If these Eq. (**17**) conditions were not to be met, it would raise the question of what factors determined the lower penetrance in (G3). If these factors were potentially identifiable and non-hereditary, then they would constitute environmental events and, thus, these genotypes would be in (G0); not in (G3). Although a stochastic mechanism might lower the penetrance somewhat, such a mechanism seems unlikely to reduce the penetrance of "purely genetic" MS markedly. Using these Eq. (**17**) conditions, we will first consider the most "extreme" circumstance, in which we assume that:

(18) $P(G3 \mid G) = P(G1 \mid G) = p$; $P(MS \mid G3) = \mathbf{x} \approx 1$; and: $P(MS \mid G2) = \mathbf{y} \approx 0$

where the variances of the of the ($\mathbf{x_i}$) and ($\mathbf{y_i}$) terms (σ_{xi}^2 and σ_{yi}^2 ; respectively) are assumed to be zero.

(19) In this circumstance: $0.081 \geq \mathbf{z} = p\mathbf{x} + (1-p)\mathbf{y} = p\mathbf{x} = p$ # Prop. (3.1a) & Eq. (**8**)

(20) Thus, under these conditions: $p \leq 0.081$

Even if we assume that the Eq. (**17**) conditions are satisfied by any: $\mathbf{x} > 0.8$

Then, Eq. (**19**) still yields: $p < 0.101$

It is noteworthy, however, that these extreme conditions are clearly contrary to <u>observed</u> epidemiological facts. Thus, under these particular extreme conditions we would also expect that:

$$P(G3 \mid G, MS) = q = q' = (p\mathbf{x}) / \{p\mathbf{x} + (1-p)\mathbf{y}\} = (p\mathbf{x}) / (p\mathbf{x}) = 1$$

Because: $P(G3 \mid G, MS) = P(G3 \mid G, IG_{MS})$ # Assumptions (A1) & (A3)

Therefore, in this circumstance, we would further anticipate that:

(21) $P(MS \mid G, IG_{MS}) = \mathbf{b'} = q\mathbf{x} + (1-q)\mathbf{y} = q\mathbf{x} \approx 1$ # Prop. (3.2a)

Consequently, the fact that: $0.134 \leq \mathbf{b'} \leq 0.143$ # Prop. (5.2b)

Indicates that the Eq. (**18**) conditions (even at: $\mathbf{x} > 0.8$) are very far removed from the actual data.

7.2b. Next we will consider an alternative set of "more plausible" extreme conditions. By Props. (2.1 & 2.2), any variance in the penetrance value within the (G3) or (G2) subset, will lead to the enrichment of more penetrant genotypes when moving either from (G) to the set (G, MS) or from (G, IG_{MS}) to the set (G, MS, IG_{MS}). Therefore, in the new "extreme" condition, we will assume that all of the enrichment that takes place is due to the difference in penetrance between the (G3) and (G2) subsets and, thus, where the variances of the of the ($\mathbf{x_i}$) and ($\mathbf{y_i}$) terms (σ_{xi}^2 and σ_{yi}^2) are still assumed to be zero. Thus, using these definitions, these modified "extreme" conditions then become:

(22) $P(G3 \mid G) = P(G1 \mid G) = p$; $P(MS \mid G3) = \mathbf{x} \approx 1$; and: $P(MS \mid G2) = \mathbf{y}$

(23) where: $\sigma_{xi}^2 = \sigma_{yi}^2 = 0$; so that: $q = q'$

(24) $\mathbf{b'} = q\mathbf{x} + (1-q)\mathbf{y} = \{p\mathbf{x}^2 + (1-p)\mathbf{y}^2\} / \mathbf{z}$ # Prop. (3.2c)

$\mathbf{z} = p\mathbf{x} + (1-p)\mathbf{y}$ # Prop. (3.1a)

(25) With rearrangement this yields: $\mathbf{y} = (\mathbf{z} - p\mathbf{x}) / (1-p)$

Substituting Eq. (**25**) into Eq. (**24**), together with conditions from Eqs. (**22** & **23**), yields:

$$\mathbf{zb'} = p\mathbf{x}^2 + (\mathbf{z} - p\mathbf{x})^2 / (1-p)$$

or: $$\mathbf{z}(1-p)\mathbf{b'} = p\mathbf{x}^2 - p^2\mathbf{x}^2 + \mathbf{z}^2 - 2p\mathbf{xz} + p^2\mathbf{x}^2 = p\mathbf{x}^2 + \mathbf{z}^2 - 2p\mathbf{xz}$$

(26) With rearrangement, this becomes: $p = (\mathbf{zb'} - \mathbf{z}^2) / (\mathbf{x}^2 - 2\mathbf{xz} + \mathbf{zb'})$

Therefore, using the limits set for (**z**) and (**b'**) by Eq. (**8**) & Prop. (5.2b):

Eq. (**26**) can be solved at: $\mathbf{x} = 1$; yielding: $p = P(G3 \mid G) \leq 0.006$

Eq. (**26**) can be solved at: $\mathbf{x} > 0.8$; yielding: $p = P(G3 \mid G) < 0.010$

Moreover, because the conditions that: $P(G3 \mid G) = P(G1 \mid G) = p$; and: $\sigma_{yi}^2 = 0$

seem too extreme for the actual distribution, and because less extreme assumptions lead to smaller estimates, these derived upper limits for the ranges of P(G3) are, almost certainly, too large.

Therefore, it must be the case that: $P(G3 \mid G) \approx 0$

And, consequently, for all practical purposes, "purely genetic" MS does not exist.

Proposition 8: **1.** **a.** P(S–│G) ≈ P(HLA–│MS) / P(HLA–) = 0.59

b. 0.41 < P(S+│G) ≤ 0.415

Thus: P(S–, S+) ≤ 0.007

<u>New Definitions for Proposition 8:</u> (see Section B for the definition of a susceptible genetic combination)

1. (S+) = the set of individuals who possess a combination of susceptibility alleles, which includes the DRB1*1501 allele, that, by itself, is sufficient to make the person susceptible to MS.
2. (S–) = the set of individuals who possess a combination of susceptibility alleles, not including the DRB1*1501 allele, that, by itself, is sufficient to make the person susceptible to MS.
3. A person is in both sets (S+) and (S–) if, in addition to the combination that makes them a member of the set (S–), they also possess another combination that make them a member of (S+).

<u>Assumption:</u>

A9. P(HLA+│S–) ≈ P(HLA+) # (HLA+) status is independent of (S–) status

<u>Proof of Proposition 8.1</u>

8.1a. From Props. (1.7 & 6.3b): P(MS│G, HLA+) ≈ P(MS│G, HLA–) ≈ P(MS│G)

(1) and, by extension: P(MS│G, S+) ≈ P(MS│G, S–) ≈ P(MS│G, HLA–, S–) ≈ P(MS│G)

Also, because: P(S–│MS, G, HLA–) = 1 # (MS, G, HLA–) ⊂ (S–)

(2) then: P(HLA–, S–│MS, G) = P(HLA–│MS, G)*P(S–│MS, G, HLA–) = P(HLA–│MS, G)

Also, from Eq. (**1**):

(3) P(HLA–, S–│MS, G) = P(HLA–, S–│G)*P(G)*P(MS│G, HLA–, S–) / P(MS, G) ≈ P(HLA–, S–│G)

(4) and: P(HLA–, S–│G) = P(S–│G)*P(HLA–│G, S–) = P(S–│G)*P(HLA–│S–) # (S–) ⊂ (G)

or equivalently, from Eqs. (**2**–**4**):

(5) P(HLA–, S–│MS, G) = P(HLA–, S–│G) = P(HLA–│MS, G) = P(S–│G)*P(HLA–│S–)

Thus, any person who belongs to the set (S–│G) has only a P(HLA–│S–) chance of also being (HLA–).

Compared to P(HLA–), the presence of other susceptibility alleles or genes at the DRB1 locus will

make P(HLA– | S–) larger and the presence of protective alleles or genes will make P(HLA– | S–) smaller. Nevertheless, these other alleles/genes are low in frequency and small in contribution compared to the DRB1*1501 allele.[26] In addition, with approximately 50–200 susceptibility loci and only 11–18 necessary for susceptibility,[27] it seems likely that most of (S–) will consist of combinations not including the DRB1 locus.

Therefore, we will assume that: $P(HLA+ | S–) \approx P(HLA+) = 0.24$ # Assumption (A9)

However, because: $P(S–) = P(HLA+, S–) + P(HLA–, S–) \approx \{P(HLA+) + P(HLA– | S–)\}*P(S–)$

Therefore, Assumption (A9) also implies that: $P(HLA– | S–) \approx P(HLA–) = 0.76$

(6) Also, because: $P(HLA– | MS, G) = P(G | MS)*P(G, HLA– | MS) \approx P(HLA– | MS)$ # Prop 5.2b: $g \approx 1$

Therefore, based on Eqs. (**5** & **6**), and on Assumption (A9):

(7) $P(S– | G) \approx P(HLA– | MS) / P(HLA–) = (0.45 / 0.76) = 0.59$

Without Assumption (A9): $0.45 \leq P(S– | G) \leq 0.59$

And: $P(S–) = P(S–, G) = P(S– | G)*P(G) \leq (0.59)(0.022) = 0.013$ # (S–) ⊂ (G) & Prop. (4.2)

(8) So that: $0.996 > 1 - P(S–) \geq 0.987$ # NB: this doesn't require Assumption (A9)

8.1b. Because by definition, the different susceptibility loci are pair-wise independent, therefore, if (S–) consists of genetic combinations not including a susceptible state at the DRB1 locus, then:

$$P(S+, S–) = P(S+)*P(S–)$$

By contrast, because membership in (S+) implies the presence of at least 1 of the DRB1 alleles is 1501, therefore, if membership in (S–) is due to a "non-1501" susceptible state at the DRB1 locus, then:

$$P(S+, S–) = P(S+)*P(S– | S+) \; ; \quad \text{and:} \quad P(S– | S+) < P(S–)$$

Consequently, in any case:

(9) $P(G) = P(S+) + P(S–) - P(S+, S–) \geq P(S+) + P(S–) - P(S+)*P(S–)$

thus: $P(G) - P(S–) \geq P(S+)*\{1 - P(S–)\}$

so that: $P(S+) \leq [P(G) - P(S–)] / [1 - P(S–)]$

(10) or: $P(S+ | G) \leq [1 - P(S– | G)] / [1 - P(S–)]$ # Dividing both sides by P(G)

Using Eq. (**10**) and making Assumption (A9) yields:

(11) $P(S+ \mid G) \approx (0.41) / [1 - P(S-)]$

Therefore, from Eqs. (**8** & **11**), in the most likely case (i.e., making Assumption A9):

$0.41 < P(S+ \mid G) \leq 0.415$ # When: $P(S- \mid G) = 0.59$

However, without making Assumption (A9), at the other extreme, this would become:

$0.45 < P(S- \mid G) \leq 0.457$ # When: $P(S+ \mid G) = 0.55$

Therefore, in any case: $P(S+, S-) \leq 0.007$

Appendix S1; Section F

Response to Environmental Events

1. Environmental Considerations …………………………….….…. p.1

2. Environmental Responses ……………………………………...… p.2

3. Gender-Specific Differences in Hazard-Rate.................…….…… p.5

4. Gender-Specific Differences in Exposure.....................……..…… p.6

Large Red Rectangles above represent hyperlinks to main parts of Section F

Small Red Boxes within Document represent hyperlinks within Appendix S1.

Small Green Boxes within Appendix S1 are hyperlinks back to Main Text

Navigate Back to Main Menu

Assumptions:

A10. Because: $P(MS, G) \geq (0.94)*P(MS)$ # Prop. (5.2b)

therefore: $P(MS, G) \approx P(MS)$

We assume also that: $P(MS, E) \approx P(MS)$; and, consequently: $P(MS, G, E) \approx P(MS)$

A11. The hazard-rate (at different exposures) for developing MS in susceptible men and women is proportional

Definitions:

1. Time-Period–1 = (1941–1945) ; Time-Period–2 = (1976–1980)

– these are indicated in the text by subscripts (1) and (2)

e.g., $P(MS_1)$ and (Zw_1) represent P(MS) and (Zw) during Time-period–1

2. Zm, Zw = probability of developing MS in susceptible men $\{P(MS, E \mid G, M)\}$ and women $\{P(MS, E \mid G, F)\}$.

By Assumption (A10): $P(MS, E \mid G, M) = P(MS \mid G, M)$; and: $P(MS, E \mid G, F) = P(MS \mid G, F)$

2. C = the proportionality constant for disease prevalence – such that: $C = P(MS_1) / P(MS_2)$

3. u , x = Actual (u) and transformed (x) exposure-levels (all necessary factors) of the susceptible population

$x_2 - x_1 = 1$; Exposure-difference between the 2nd (x_2) and 1st (x_1) time-period is defined as "1 unit"

4. h(u) , g(u) = hazard-functions for developing MS in susceptible men {h(u)} and women {g(u)}

r = the proportionality constant for hazard – such that: $g(u) = (r)\cdot h(u)$

5. λ_m , λ_w = Exposure-threshold necessary to produce disease in susceptible men (λ_m) and women (λ_w)

$\lambda = \lambda_w - \lambda_m$ = the difference in exposure-threshold between susceptible women and men

6. **c** , **d** = the maximum probability of MS in genetically susceptible men (**c**) and women (**d**).

i.e., $\mathbf{c} = P(MS \mid G, E, M)$; and: $\mathbf{d} = P(MS \mid G, E, F)$

7. $P(F_1)$, $P(F_2)$ represent (and are interchangeable with) $P(F \mid MS_1)$ and $P(F \mid MS_2)$ respectively

$P(M_1)$, $P(M_2)$ represent (and are interchangeable with):

$P(M \mid MS_1) = 1 - P(F_1)$; and: $P(M \mid MS_2) = 1 - P(F_2)$; respectively

Environmental Considerations

From Prop. (6.2), it is apparent that the greater prevalence of MS in women is due to:

$$P(MS, E \mid F, G) > P(MS, E \mid M, G)$$

This could be due to women being more likely to experience a sufficient environmental exposure than men, to women having a different physiological response to a similar exposure compared to men, to

women having a greater probability of developing MS once the necessary environmental and genetic events have come together, or it could be due to some combination of these factors. Regardless of the reason, however, women and men require separate consideration so that:

(1) for women: $P(G, F)*P(MS, E \mid G, F) = P(MS, F) = P(MS)*P(F \mid MS)$ # Assumption (A10)

(2) for men: $P(G, M)*P(MS, E \mid G, M) = P(MS, M) = P(MS)*P(M \mid MS)$ # Assumption (A10)

Because the genetics of MS in Canada are unlikely to have changed substantially between the two time-periods (i.e., 35 years, or 1-2 generations)[15], the {P(G), P(G | F), and P(G | M)} terms are assumed to be constant over this interval. In this case, the constant (C), representing the change in the disease prevalence: $P(MS_1) = (C)*P(MS_2)$; reflects the change in environmental exposures over time.

Thus: $P(MS_1)*P(F \mid MS_1) = (C)*P(MS_2)*P(F \mid MS_1) = (C)*P(MS_2)*P(F_1)$ # Definition (7)

From Eq. (**1**) $P(MS_1, E \mid G, F) = P(MS_1)*P(F \mid MS_1) / P(G, F) = P(F_1)(C)*P(MS_2) / P(G, F)$

In Canada, the sex-ratio in MS patients {i.e., P(F | MS) / P(M | MS)} has increased from 2.2 in Time-Period–1 (i.e., 1941-1945) to become 3.2 in Time-Period–2 (i.e., 1976-1980).[15]

Consequently:

$$P(MS, E \mid G, F)_2 = Zw_2 = P(F_2)*P(MS_2) / P(G, F)$$

(3) $$P(MS, E \mid G, F)_1 = Zw_1 = P(F_1)(C)*P(MS_2) / P(G, F) = \{P(F_1)/P(F_2)\}C(Zw_2)$$

(4) and, similarly: $P(MS, E \mid G, M)_1 = Zm_1 = P(M_1)(C)*P(MS_2) / P(G, M) = \{P(M_1)/P(M_2)\}C(Zm_2)$

Environmental Responses

From standard Survival Analysis methods, we define the cumulative survival function {S(u)}, the cumulative failure function, {F(u)}, and the hazard-functions and for men {h(u)} and for women {g(u)}.

From Assumption (A11): $g(u) / h(u) = r$

Also, defining {H(u)} as the definite integral of the hazard-function {h(u)} from a (u) level of exposure to a (0) level, we can transform (u) units of exposure into (x) units such that [x = H(u)]. Thus, for men:

(5) $$\ln [S(u)] = -\int_0^u h(u)du = -\int_0^x dx = -x$$

Because we have assumed proportional hazard, therefore, for women:

(6) $$\ln[S(u)] = -\int_0^x (r)dx = -(r)x$$

Taking the anti-log of both sides of Eqs. (**5** & **6**) yields:

$S(u) = e^{-rx}$ and, thus: $F(u) = (1 - e^{-rx})$ # By definition: $(r = 1)$ for men

Also, we can define (as 1 exposure-unit) the difference in exposure between any two time-periods

(7) (x_1) and (x_2), such that: $x_2 - x_1 = 1$

This definition transforms the exposure units from (u) to (x) and yields an apparently constant hazard-rate for both men and women, even though (x) may not increment the actual exposure linearly.

Thus, the cumulative probability of failure (i.e., of developing MS in susceptible persons), in the circumstance where every susceptible person fails given sufficient exposure (E), is described by:

$F(u) = P(MS, E \mid G) = P(MS \mid E, G)*P(E \mid G) = P(E \mid G)$ # when: $P(MS \mid E, G) = 1$

(8) Therefore: $F(u) = 1 - S(u) = (1 - e^{-rx}) = P(E \mid G, F)$ for women in this circumstance

(9) and: $F(u) = 1 - S(u) = (1 - e^{-x}) = P(E \mid G, M)$ for men in this circumstance

However, unlike true survival analysis (where everyone dies given enough time), the probability of developing MS may not increase to 100% as the level of environmental exposure increases (see Section B). Also, men and women may not approach the same limiting value for this probability. Finally, the level of environmental exposure at which the development of MS become possible (i.e., the threshold) does not need to occur at zero and the threshold does not need to be the same for men and women. Consequently, Eqs. (**8** & **9**) need to be written differently such that:

(10) $P(MS, E \mid G, F) = Zw = \mathbf{d}\{1 - e^{-r(x - \lambda m - \lambda)}\}$ for women

(11) and: $P(MS, E \mid G, M) = Zm = \mathbf{c}\{1 - e^{-(x - \lambda m)}\}$ for men

The terms {**c** $= P(MS \mid G, E, M)$ and (**d** $= P(MS \mid G, E, F)$} are positive constants that represent the conditional probability that susceptible men and women will develop MS given a maximum level of sufficient environmental exposure {i.e., where: $P(E \mid G, M) = 1$; and: $P(E \mid G, F) = 1$}. If (**c**) and (**d**) are equal, then men and women approach the same limiting probability of developing MS. If (**c**) and (**d**) are

both 1.0, then, as for true survival, everyone ultimately fails. If the threshold in women (λ_w) is greater than that in men (λ_m), then the difference in threshold (λ) will be positive. Because, Assumption (A11) leads to exponential response curves, any two points determines each curve uniquely.

Thus, from Eqs. (**3**, **4**, **7**, **10**, & **11**), and the range-estimates developed in Prop. (7.1c):

(12) $$0.096 \le Zw_2 = \mathbf{d}^*\{1 - e^{-r(x1 + 1 - \lambda m - \lambda)}\} \le 0.191$$

(13) $$0.030 \le Zm_2 = \mathbf{c}^*\{1 - e^{-(x1 + 1 - \lambda m)}\} \le 0.040$$

(14) $$0.087C \le Zw_1 = \mathbf{d}^*\{1 - e^{-r(x1 - \lambda m - \lambda)}\} \le 0.172C$$

(15) $$0.039C \le Zm_1 = \mathbf{c}^*\{1 - e^{-(x1 - \lambda m)}\} \le 0.053C$$

Although the prevalence of MS is increasing, it seems unlikely that it could have more than quadrupled in Canada over a 35 year interval.[4, 15] Consequently: $C > 0.25$

Eq. (**12**) can be rearranged to yield: $(Zw_2 - \mathbf{d}) / \mathbf{d} = -(e^{-r(x1 - \lambda m - \lambda)})e^{-r}$

Similarly, Eq. (**14**) can be rearranged to yield: $(Zw_1 - \mathbf{d}) / \mathbf{d} = -(e^{-r(x1 - \lambda m - \lambda)})$

(16) And, therefore, dividing these yields: $(Zw_2 - \mathbf{d}) / (Zw_1 - \mathbf{d}) = e^{-r}$

(17) Similarly, rearranging Eqs. (**13** & **15**) yields: $(Zm_2 - \mathbf{c}) / (Zm_1 - \mathbf{c}) = e^{-1}$

(18) Combining Eqs. (**3** & **16**) yields: $\mathbf{d} = Zw_2\{1 - (F_1 / F_2)Ce^{-r}\} / (1 - e^{-r})$

(19) Combining Eqs. (**4** & **17**) yields: $\mathbf{c} = Zm_2\{1 - (M_1 / M_2)Ce^{-1}\} / (1 - e^{-1})$

(20) Also: $Zm_2 < \mathbf{c} = [Zm_2 - (Zm_1)e^{-1}] / [1 - e^{-1}]$ # Eq. (**17**)

and: $[Zm_2 / (1 - e^{-1})] - Zm_2 > (M_1 / M_2)(Zm_2)Ce^{-1} / (1 - e^{-1})$ # Eqs. (**4**), (**19**) & (**20**)

Therefore, based only on the observed change in the sex-ratio:

(21) $$C < \{[1 / (1 - e^{-1})] - 1\} / \{(M_1 / M_2)e^{-1} / (1 - e^{-1})\} = 0.76$$

When: $r = 1$; Eqs. (**12**–**15**) can be rearranged to yield:

$$e^{\lambda} = [\mathbf{c}/\mathbf{d}][(Zw_2 - \mathbf{d}) / (Zm_2 - \mathbf{c})] = [\mathbf{c}/\mathbf{d}][(Zw_1 - \mathbf{d}) / (Zm_1 - \mathbf{c})]$$

(22) so that: $$\lambda = \ln\{[\mathbf{c}/\mathbf{d}][(Zw_2 - \mathbf{d}) / (Zm_2 - \mathbf{c})]\} = \ln\{[\mathbf{c}/\mathbf{d}][(Zw_1 - \mathbf{d}) / (Zm_1 - \mathbf{c})]\}$$

(23) Using an estimate of: $0.25 \leq C \leq 0.75$ # based on Eq. (**21**)

Assuming ($r \approx 1$), together with Eqs. (**12–15**, **18**, **19**, & **22**), yields the estimates of:

(24) $0.030 \leq \mathbf{c} \leq 0.056$; $0.114 \leq \mathbf{d} \leq 0.277$

(25) $0.100 \leq \lambda \leq 2.87$; $2.5 \leq \mathbf{d/c} \leq 7.5$

From Eqs. (**12** & **14**), clearly, (**d**) is independent of (r) for all ($r > 0$). Thus, in Eq. (**14**), the second point (Zw_1), defining the exponential curve, is expressed only in terms of (C); not (r). The same is true for the parameter (**c**), as can be appreciated from Eq. (**19**).

Thus, the estimates for (**c**), (**d**), and (**d**/**c**) depend only upon (C) and the observed sex-ratio change. By contrast, the estimate for (λ) depends upon (C), (r), and the sex-ratio change.[4] These relationships, described by Eqs. (**10** – **15** & **24** – **25**), are depicted graphically in Figure 1.

Also from Eqs. (**3** & **16**): $r = -\ln\{(Zw_2 - \mathbf{d}) / ([P(F_1)C(Zw_2) / P(F_2)] - \mathbf{d})\}$

(26) Using the range-estimates from Eqs. (**12**, **23**, & **24**) yields: $0.54 \leq r \leq 1.6$

Gender-Specific Differences in Hazard-Rate

For susceptible women, we define three terms (x_1^{app}), (λ_w^{app}), and (λ^{app}) such that:

$$x_1^{app} = (r)x_1 \ ; \quad x_1^{app} - \lambda_w = x_1 - \lambda_w^{app} \ ; \quad \text{and:} \quad \lambda^{app} = \lambda_w^{app} - \lambda_m$$

From these definitions, it follows that:

$$x_1^{app} - x_1 = (r-1)x_1 = \lambda_w - \lambda_w^{app}$$

(27) and: $\lambda^{app} = (x_1 - x_1^{app}) + \lambda_w - \lambda_m = (1-r)x_1 + \lambda$

The transformation of (λ_w) to (λ_w^{app}) effectively creates (at the exposure x_1) an apparent circumstance, in which ($r = 1$). Consequently, we can use the lower bound of ($\lambda \geq 0.10$) from Eqs. (**22** & **24**) to express the apparent difference in threshold (λ^{app}) between men and women, such that:

$$\lambda^{app} = \ln\{[\mathbf{c/d}][(Zw_1 - \mathbf{d}) / (Zm_1 - \mathbf{c})]\} \geq 0.10$$

in which case: $\lambda^{app} = (1-r)x_1 + \lambda \geq 0.10$ # From Eq. (**27**)

Therefore: $\lambda \geq 0.10$; for all ($r \geq 1$) # By definition: $x_1 > 0$

Consequently, assuming a proportional hazard for men and women, susceptible men (compared to susceptible women) must have a lower threshold, a greater hazard-rate, or both (see Figure 1).

Gender-Specific Differences in Exposure

If there are three environmental events (E_A, E_B, and E_C) necessary to produce MS,[4] each of which is both equally likely and conditionally independent (with respect to gender and susceptibility), then, for women:

$$P(MS, E \mid G, F) = P(MS \mid G, E, F)*P(E \mid G, F) = (\mathbf{d})*P(E_A, E_B, E_C \mid G, F) = (\mathbf{d})*\{P(E_A \mid G, F)\}^3$$

and for men:

$$P(MS, E \mid G, M) = P(MS \mid G, E, M)*P(E \mid G, M) = (\mathbf{c})*P(E_A, E_B, E_C \mid G, M) = (\mathbf{c})*\{P(E_A \mid G, M)\}^3$$

Because, by Assumption (A10), and considering Time-Period–2, then:

$$P(MS, E \mid G, M)_2 = P(MS \mid G, M)_2 = Zm_2$$

and: $$P(MS, E \mid G, F)_2 = P(MS \mid G, F)_2 = Zw_2$$

Therefore, using the range-estimates from Eqs. (**12**, **13**, & **24**) yields, for women:

$$0.690 = (0.191) / (0.277) \leq \{P(E_A \mid G, F)_2\}^3 = Zw_2 / \mathbf{d} \leq 1$$ # Ratio smallest for high Zw_2

(**28**) or: $$0.88 \leq P(E_A \mid G, F)_2 \leq 1$$

and, for men:

$$0.714 = (0.040) / (0.056) \leq \{P(E_A \mid G, M)_2\}^3 = Zm_2 / \mathbf{c} \leq 1$$ # Ratio smallest for high Zm_2

(**29**) or: $$0.89 \leq P(E_A \mid G, M)_2 \leq 1$$

Even dropping the assumption of three, equally likely, conditionally independent events, a sufficient environmental exposure (whatever this entails) must be experienced by more than 69% of the

(30) susceptible population. Thus, from the above, we conclude that: $0.690 \leq P(E \mid G) \leq 1$

(31) If envirnmental experience is independent of susceptibility, then: $0.690 \leq P(E) \leq 1$

Consequently, at present, both genders seem to experience, very commonly, each of the necessary environmental events involved in MS pathogenesis, (i.e., these are population-wide events).

Of course, from Eq. (**24**), for both men and women: $P(MS \mid G, E) << 1$

Thus, it must be that certain genetic backgrounds are only (or more) responsive to certain environmental experiences. For example, if all genotypes required the (E_A) environmental event (e.g., vitamin D deficiency)[4] but some genotypes required a longer duration or greater intensity of exposure to produce MS than others, then this might help to explain the low penetrance ranges for the parameters (**c**) and (**d**) incicated in Eq. (**24**).
